# Airway Microbiota and Pathogen Abundance in Age-Stratified Cystic Fibrosis Patients

**DOI:** 10.1371/journal.pone.0011044

**Published:** 2010-06-23

**Authors:** Michael J. Cox, Martin Allgaier, Byron Taylor, Marshall S. Baek, Yvonne J. Huang, Rebecca A. Daly, Ulas Karaoz, Gary L. Andersen, Ronald Brown, Kei E. Fujimura, Brian Wu, Diem Tran, Jonathan Koff, Mary Ellen Kleinhenz, Dennis Nielson, Eoin L. Brodie, Susan V. Lynch

**Affiliations:** 1 Department of Medicine, University of California San Francisco, San Francisco, California, United States of America; 2 Department of Anesthesia and Perioperative Care, University of California San Francisco, San Francisco, California, United States of America; 3 Ecology Department, Lawrence Berkeley National Laboratory, Berkeley, California, United States of America; 4 Department of Plant and Microbial Biology University of California, Berkeley, California, United States of America; 5 Adult Cystic Fibrosis Program, University of California San Francisco, San Francisco, California, United States of America; 6 Pediatric Cystic Fibrosis Program, University of California San Francisco, San Francisco, California, United States of America; Columbia University, United States of America

## Abstract

Bacterial communities in the airways of cystic fibrosis (CF) patients are, as in other ecological niches, influenced by autogenic and allogenic factors. However, our understanding of microbial colonization in younger versus older CF airways and the association with pulmonary function is rudimentary at best. Using a phylogenetic microarray, we examine the airway microbiota in age stratified CF patients ranging from neonates (9 months) to adults (72 years). From a cohort of clinically stable patients, we demonstrate that older CF patients who exhibit poorer pulmonary function possess more uneven, phylogenetically-clustered airway communities, compared to younger patients. Using longitudinal samples collected form a subset of these patients a pattern of initial bacterial community diversification was observed in younger patients compared with a progressive loss of diversity over time in older patients. We describe in detail the distinct bacterial community profiles associated with young and old CF patients with a particular focus on the differences between respective “early” and “late” colonizing organisms. Finally we assess the influence of Cystic Fibrosis Transmembrane Regulator (CFTR) mutation on bacterial abundance and identify genotype-specific communities involving members of the Pseudomonadaceae, Xanthomonadaceae, Moraxellaceae and Enterobacteriaceae amongst others. Data presented here provides insights into the CF airway microbiota, including initial diversification events in younger patients and establishment of specialized communities of pathogens associated with poor pulmonary function in older patient populations.

## Introduction

Chronic lung function decline, punctuated by recurrent airway exacerbations is characteristic of CF pulmonary disease and contributes substantially to patient morbidity and mortality [1]. Several culture-independent studies examining bacterial diversity in a variety of human niches have demonstrated that shifts in bacterial community composition underlie states of host health or disease [2], [3], [4], [5], [6], [7], [8]. The advent of high-throughput technologies to examine microbial community composition permits high-resolution profiling of organisms that are beneficial or detrimental to the health of the human super-organism [9]. Recent studies focusing on CF have demonstrated that bacterial assemblages, that include a number of pathogens, exist in both adult and pediatric airways [8], [10], [11], [12], [13], [14], [15], [16]. These assemblages undoubtedly contribute to the long-term lung function decline experienced by CF patients, but the depth of bacterial diversity and the relationship between airway microbiota and chronic pulmonary disease observed in patients from birth through adulthood, is currently unknown.

To perform an in-depth characterization of age-based bacterial community composition of CF airways, independent deep-throat swab and expectorated sputum samples from 51 pediatric and adult patients respectively, were collected at the pediatric and adult CF clinics at University of California, San Francisco. Analysis was performed using the 16S rRNA PhyloChip, a high-density, phylogenetic microarray, capable of identifying approximately 8,500 bacterial taxa in parallel [17], [18], [19].

## Materials and Methods

### Ethics Statement

The Committee on Human Research at University of California San Francisco (UCSF) approved all study protocols, and all patients or surrogates provided written, informed consent.

### Sample collection

Airway samples from clinically stable adult and pediatric CF patients, defined as having no change in pulmonary function for ≥2 months prior to sample collection were used for this study. Adult sputum samples (n = 37) were collected following routine spirometry at the UCSF Adult Cystic Fibrosis Centre clinic. Samples were mixed with approximately twice the volume of RNAlater (Ambion) prior to nucleic acid extraction within 4 hours of collection. Pediatric deep-throat swab samples (n = 26) were collected at the UCSF Pediatric Cystic Fibrosis Centre clinic from all patients (aged up to 18 years), submerged in a sterile tube containing 3 ml of RNALater prior to nucleic extraction within 4 hours of collection. Deep-throat swabs were used as an alternative to sputum since the majority of pediatric patients, particularly the younger individuals are unable to produce sputum. The primary dataset for this study comprised single, independent samples from 51 patients. To determine the temporal changes in bacterial diversity within patients of different age groups, a subset of 13 of these patients from whom we had also collected a subsequent stable sample were analyzed. Patient demographics are presented in Table S4.

### Sample processing

Optimized DNA extraction protocols developed in-house using CF sputum samples and representative Gram positive and Gram negative bacteria were used for this study. Adult samples were centrifuged for 10 minutes at 12,000 g and RNALater removed prior to total genomic DNA extraction from 300 µl of sputum (containing sputum plugs) using the Wizard Genomic DNA extraction kit according to the manufacturer's instructions (Promega, CA). Deep-throat swabs were added to lysis buffer from the AllPrep DNA/RNA extraction kit (Qiagen, CA) in a lysing matrix B tube (Qbiogene, CA) and lysed by bead-beating using a FastPrep system (Qbiogene, CA) for 30 seconds at 5.5 m sec^−1^. Supernatant was then transferred to the DNA column of the AllPrep kit and nucleic acids extracted according to the manufacturer's instructions. Universal primers 27F (5′-AGAGTTTGAT CCTGGCTCAG-3′) and 1492R (5′-GGTTACCTTGTTACGACTT-3′) [20] were used to amplify the 16S rRNA gene using 12 PCR reactions per sample run across a gradient of annealing temperatures (48–58°C) to maximize diversity recovered. PCR reactions contained 0.02 Uµl^−1^ Takara Ex Taq DNA Polymerase (Takara Bio Inc Japan), 1× Takara buffer, 0.8 mM Takara dNTP mixture, 0.4 mg ml^−1^ bovine serum albumin (BSA) and 1.0 µM of each primer. PCR conditions were 1 cycle of 3 min at 95°C followed by 25 cycles of 95°C for 30 s, the gradient annealing temperature for 30 s, 72°C for 2 min and a final extension at 72°C for 10 min. A total of 100 ng of extracted DNA from adult sputum or pediatric deep-throat swab samples was used per PCR reaction. Amplified products from all 12 annealing temperatures were pooled, gel-purified and processed for PhyloChip analysis as previously reported [17], except that 250 ng of each amplicon was hybridized. As a negative control, nucleic acid was extracted from sterile swabs and assayed for 16S rRNA product as described above. No PCR product was detected.

### Microarray analysis

A two-step normalization procedure was adopted to correct the fluorescence intensities for amplicon target quantification variation and in microarray technical variation. For analysis of bacterial community composition we used the PhyloChip, a 16S rRNA custom microarray developed at Lawrence Berkeley National Laboratory and synthesized by Affymetrix Inc. (Santa Clara, CA, USA). The array has 506,944 probes arranged in 712 rows and columns representing approximately 8,500 bacterial taxa, with the capability of detecting bacteria comprising at least 0.01% of a population [21]. Each chip has additional probes that serve as the following controls: (1) prokaryotic and eukaryotic metabolic genes (added prior to fragmentation) to control for variations in fragmentation, biotinylation, hybridization, washing, staining, and scanning and (2) pre-labeled oligonucleotides added to the hybridization mix to account for variation in hybridization, washing, staining, and scanning. In addition, to minimize cross-hybridization, at least eleven probe pairs (positive match and mis-match oligonucleotides) are used to interrogate each taxon at at least two discriminatory loci on the 16S rRNA gene. Combined PCRs and control amplicons were fragmented to 50–200 bp using DNase I (0.02 U mg^−1^ DNA; Invitrogen, USA) and One-Phor-All buffer (NJ, USA). Biotin labeling was performed using terminal deoxynucleotide transferase and ddUTP as per the manufacturer's instructions. The reactions were denatured at 99°C for 5 min and hybridized to the PhyloChip for 16 hours at 48°C at 60 rpm. The arrays were subsequently washed, stained and scanned as in [22]. Scanning of the arrays was performed using a GeneArray Scanner (Affymetrix, CA, USA) and the intensity of all the probes was treated as previously reported [17], [19]. Positive probe pairs met two criteria: (1) the fluorescence of the perfectly matched probe was at least 1.3 times greater than the intensity of the control (mismatch probe); (2) the value of the difference between perfectly matched probe and mismatch probe intensities was at least 130 times greater than the squared noise value. The value of the positive fraction (pf) was calculated for each probe set as the number of positive probe pairs divided by the total number of probe pairs in a probe set.

Data sets were conservatively filtered, with taxa determined as present if pf ≥0.9 (90% of probes in a probe set for an individual taxon) were positive. Changes in probe-set fluorescence intensity are equivalent to changes in taxon relative abundance between samples. Fluorescence intensity for every taxon determined to be present in at least one sample was log transformed prior to analysis using packages in the R statistical environment [23]. FastUnifrac (Hamady *et al*, in review [24]) with branch length weighting by taxon relative abundance (normalized log-transformed fluorescence intensity) was used to generate a phylogenetic distance matrix from the PhyloChip data.

### Quantitative PCR Validation

Quantitative PCR (Q-PCR) was performed on a subset of samples with sufficient remaining DNA to validate the presence and relative abundance of *Pseudomonas* spp., *Staphylococcus* spp., and *Haemophilus* spp. using primers designed on the Greengenes 16S rRNA gene alignment [22] using Arb [25] (*Pseudomonas*: 9056aF 5′-CCGCATACGTCCTGAGGGA GAAAGT-3′, 9056aR 5′-TCTCAGACCAGTTACGGATCGTCGC-3′; *Staphylococcus*: SaurF2 5′-AACCCTTAAGCTTAGTTGCCATC-3′, SaurR2 5′-TTGACCTCGCGGTTTC GCTG-3′; *Haemophilus*: HinF 5′-AATGGCGTATACAGAGGGAAG-3′, HinR 5′- CAATCCGGACTTAGACGTACT-3′). A total of 20 ng of DNA per reaction was used in triplicate, 25 µl Q-PCR reactions at an annealing temperature of 56°C with the Quantitect SYBR Green QPCR kit (Qiagen, MD) according to the manufacturer's instructions. Pearson's correlation analysis of inverse cycle threshold values against array fluorescence intensities was used to confirm relative abundance of the three organisms and concordance between the two independent molecular methods.

In addition to Q-PCR validation, to confirm that specific members of the community were truly present, a pair of primers designed to detect *Veillonella parvula* (based on PhyloChip probes; VDF5′-CGTAATCAA CCTGCCCTTCAGAGG -3′, VDR 5′-TTTCTGGCTTCC GAAGAAGAGGAAC-3′) was used to amplify a PCR product which was then submitted for bi-directional sequencing. Sequence identity was assigned by comparing reads to GenBank using NCBI's BLAST (http://blast.ncbi.nlm.nih.gov/).

### Data analysis

From the FastUnifrac distance matrix the amount of variance that each clinical variable *e.g.* sample collection method, age *etc.* contributed to the dataset was calculated using the *adonis* function of the *vegan* package in R [26]. Nearest-taxon index (NTI) and Net-relatedness index (NRI) [27], [28] measures of the phylogenetic-relatedness of communities, were calculated using the *picante* package in R [29]. NRI, is a standardized measure of the mean pairwise phylogenetic distance of taxa in a sample, relative to a phylogeny of an appropriate species pool, and quantifies overall clustering of taxa on a tree. NTI, is a standardized measure of the phylogenetic distance to the nearest taxon for each taxon in the sample and quantifies the extent of terminal clustering, independent of deep level clustering. A neighbor-joining with nearest-neighbor interchange phylogenetic tree of representative 16S rRNA sequences downloaded, pre-aligned from the Greengenes database [22] was constructed using FastTree [30]. FastTree is a neighbor-joining with nearest neighbor interchange phylogenetic inference method using alignment profiles rather than distance matrices. The topology of an initial neighbor-joining tree is improved by rounds of nearest neighbor interchanges, which recompute the profiles of each internal node. Splits in the tree that result from this process are then tested by local bootstrapping to see how well they are supported. This approach has the advantage over alternative methods, of speed whilst approximating the accuracy of character-based methods such as maximum-likelihood, Bayesian analysis, or parsimony. This was used, together with taxon richness, to calculate the mean phylogenetic distance (MPD) and mean nearest phylogenetic taxon distance (MNTD) using the phylogeny shuffle null model for each sample. MPD and MNTD values were used, as previously described [27], to calculate NRI and NTI values respectively for each sample. Inverse Simpson's diversity index [31], Pielou's evenness [32] and community richness were calculated using the *vegan* package in R. To accurately reflect community richness for this calculation, individual taxa were deemed to have an abundance of 0 if they did not meet the pf ≥0.9 criterion. Temporal change in community diversity was calculated by subtracting the calculated diversity index of the second sample from the initial sample collected from individual patients and normalized by dividing the result by the number of days between sample collections to provide the change in diversity over time.

Linear regression analysis was performed using calculated metrics of community composition and patient variables e.g. age, CFTR mutation etc. in Statplus (Analystsoft, Vancouver, Canada). Correlation analysis of each individual taxon abundance against patient age or CFTR mutation was performed using the *multtest* package [33] available as part of the Bioconductor suite of analysis programs [34]. P-values were adjusted for false discovery using the Benjamini-Hochberg procedure [35] or q-value method [36] where applicable. A phylogenetic tree of taxa that significantly positively or negatively correlated with age was constructed using FastTree as described above. Samples were assigned to age bins of 5 years (all bins contained ≥3 samples) and taxon fluorescence values in each bin were averaged and scaled relative to the highest value in the overall dataset. The tree illustrating these changes was imported to the Interactive Tree of Life (http://itol.embl.de/; [37]) and annotated. Taxa that were identified by Bellerophon [38] running on the Greengenes database as chimeric or those taxa represented by sequences of less than 600bp were not reported.

## Results

### Relationship between Pulmonary Function and Airway Bacterial Diversity

Sample type (deep throat swab or sputum) explained only 7% of community variance (p = 0.001), with age group explaining 33% (p = 0.001), suggesting that the method of sample collection did not substantially impact community compositional differences, although differences in the site of sample collection likely explains this low level of variance observed. Between 78 and 1012 taxa were detected in the samples and 1,837 different taxa (defined as species or strains that share≥97% 16S rRNA sequence identity) were detected in the entire cohort, which represents substantially greater bacterial richness than previously reported using other culture-independent methods [10], [11]. This is likely due to the ability of the PhyloChip to detect members that represent as little as 0.01% of the community in parallel with highly abundant taxa [18]. This aspect of array analysis is particularly advantageous when analyzing CF airway samples that tend to be dominated by a small number of highly abundant species [10]. However due to the potential for cross-hybridization at the taxon-level, the majority of our subsequent analyses was performed at higher levels of classification. These taxa belonged to 43 phyla with the majority belonging to the phyla Actinobacteria, Bacteroidetes, Firmicutes and Proteobacteria with smaller numbers belonging to the Acidobacteria, Planctomycetes and Spirochaetes (a complete list of all taxa detected is provided in Supplementary Table S1). Known CF pathogens, routinely cultured from CF sputum, were detected in the samples. *S. aureus* could be detected in 65% of samples (61% of adult samples, 72% of pediatric) and *P. aeruginosa* in 73% of samples (91% of adult samples, 39% of pediatric). A subset of 14 adult CF samples was used to examine the abundance of three organisms by Q-PCR. Correlation analysis of Q-PCR data with PhyloChip fluorescence intensity demonstrated good concordance for *P. aeruginosa* (otu_9056; r = 0.76; p≤0.001), *S. aureus* (otu_3258; r = 0.86; p≤0.0001) and *H. influenzae* (otu_8555; r = 0.56; p = 0.002). Although multiple safeguards are in place to minimize cross-hybridization, the PhyloChip, like all other culture-independent profiling technologies is susceptible to false positives. Therefore, targeted PCR amplification using primers designed on probes for OTU 940, whose representative species is *Veillonella parvula* was used to amplify a PCR product which was then sequenced. Sequence analysis demonstrated that the sequence exhibited 99% homology with *Veillonella parvula* across 631 nt.

**Figure 1:**
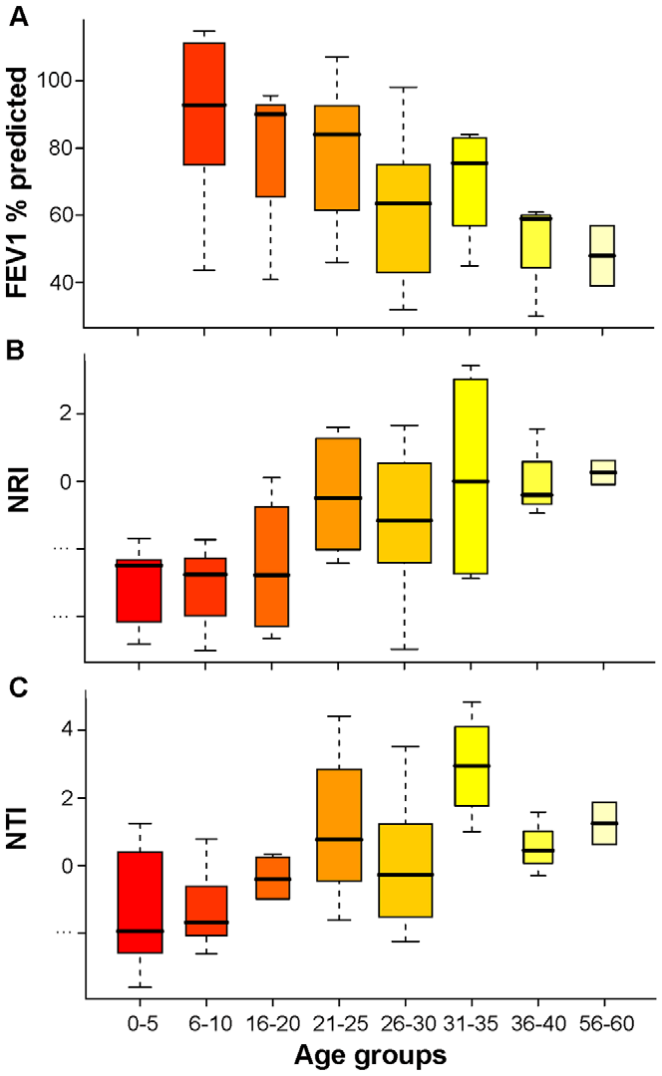
Patient age is related to pulmonary function and aspects of community relatedness. Boxplots with age bins containing three or more samples are displayed; Relationships between patient age and (A) pulmonary function (B) Net Relatedness Index and (C) Nearest Taxon Index are illustrated. As pulmonary function declines, increasing phylogenetic relatedness at both the deeper levels and terminal branches of the phylogenetic tree respectively is evident.

To determine whether a linear relationship existed between patient age and pulmonary function (forced expiration volume in one second [FEV_1_% predicted]), we tested for correlation between these variables, for samples where FEV_1_ measurements were available (n = 44). Despite the absence of standardized airway function testing for the very young pediatric patients (age ≤5, n = 7) the data confirmed that older patients CF patients exhibited significantly lower pulmonary function (r = −0.48; p<0.0003; Fig. 1A). To determine whether age-related pulmonary function was associated with aspects of the airway bacterial community structure or composition, we tested for correlations between patient age (n = 51) and taxonomic richness (number of bacterial taxa detected), community evenness (relative distribution of taxa within communities), taxonomic diversity (metric based on both richness and evenness) and phylogenetic relatedness; Net Relatedness Index (NRI) and Nearest Taxon Index (NTI), two indices that measure the degree of phylogenetic clustering or dispersion within communities [39]. These analyses revealed that older CF patients possessed airway bacterial communities that were less rich (r = −0.35, p<0.001), less even (r = −0.38, p<0.0006) and had significantly lower bacterial diversity (r = −0.33, p<0.004; Table 1). In addition, bacteria in the airway communities of older patients were more related to each other (NTI, r = 0.47, p<0.0001; NRI, r = 0.35, p<0.0001; Table 1; Fig. 1B and C) with the NTI and NRI indicating significant phylogenetic clustering of the taxa in these samples at both the terminal branches (taxon level) and at higher phylogenetic levels (family, class etc.). These data demonstrate that compared to younger CF patients, older individuals exhibit reduced airway bacterial diversity and assemblages of more closely-related organisms which occurs in parallel with age-related pulmonary function decline, implicating this structural shift in community composition in the pathophysiology of late stage CF airway disease. This has not previously been demonstrated in an age-stratified cohort spanning neonates to adults.

**Table 1:** Relationship between CF patient age and metrics of bacterial community composition.

*Analysis*	*All clinically stable*	*Homo-ΔF508*	*Hetero-ΔF508*	*Non-ΔF508*
Age vs Richness	−0.35^a^ p^b^<0.001	0.53 p<0.02	0.007 p<0.98	0.36 p<0.25
Age vs Evenness	−0.38 p<0.0006	0.08 p<0.75	0.32 p<0.12	0.35 p<0.27
Age vs Diversity	−0.33 p<0.004	0.53 p<0.02	0.0005 p<0.99	0.35 p<0.27
Age vs NRI	0.47 p<0.0001	0.37 p<0.13	0.63 p<0.005	0.62 p<0.03
Age vs NTI	0.52 p<0.0001	0.56 p<0.01	0.26 p<0.31	0.74 p<0.006

a R values.

b P values <0.05 are considered significant.

### Airway Bacterial Diversity and CFTR Genotype

Our cohort consisted of a variety of CFTR genotypes, permitting the opportunity to examine the relationship between mutation and community composition. Patients were classified in 3 groups: homozygous-ΔF508 (n = 19), heterozygous-ΔF508 (n = 19) or non-ΔF508 mutation (n = 13). To determine which aspects of community composition were most related to mutation-specific loss of pulmonary function, regression analyses against community metrics and bacterial taxon abundance were performed. Significant negative correlations between patient age and bacterial diversity, richness and NTI (Table 1) were identified for the homozygous-ΔF508 group. Compared with younger patients, community restructuring in older homozygous-ΔF508 patients was primarily due to loss of multiple members of the Mycobacteriaceae, Staphylococcaceae, Enterobacteriaceae, Acholeplasmataceae, Pasteurellaceae and Chlamydiaceae; older individuals were primarily associated with higher abundance of members of the Pseudomonadaceae (Supplementary Table S2). Compared to younger heterozygous-ΔF508 and non-ΔF508 patients, older individuals with these genotypes did not exhibit significant reductions in taxonomic richness, evenness or diversity” (Table 1); however, for both groups, these patients did exhibit increased NTI and NRI of their airway communities (Table 1). Similar to the homozygous patients, the older heterozygous-ΔF508 patients also exhibited increased abundance of members of the Pseudomonadaceae and Xanthomonadaceae; in addition, they also exhibited significant increases in the Moraxellaceae, and Sphingomonadaceae. In contrast to ΔF508 patients, non-ΔF508 subjects did not exhibit an age-based positive correlation with the Pseudomonadaceae or with any other bacterial family. Instead younger patients were strongly associated with multiple members of the Enterobacteriaceae, Campylobacteraceae and Helicobacteraceae (Supplementary Table S2). These families appear to be specifically associated with the non-ΔF508 group, as they did not feature prominently in heterozygous-ΔF508 or homozygous-ΔF508 airways.

### Temporal Diversity Changes

The temporal change in bacterial diversity of a subset of 13 patients ranging from 9 months to 43 years for whom a second, subsequent clinically stable airway sample was available was examined. All pediatric patients examined up to 11 years of age (n = 10) exhibited a net increase in bacterial diversity over time. In contrast a net decrease in community diversity was observed for older patients (n = 3; Fig. S1), suggesting that CF patients exhibit an initial expansion of airway diversity (colonization and diversification period) over the first decade of life, after which a progressive decline in diversity and development of increasingly phylogenetically-related consortia occurs (establishment of competitively dominant species). However, while provocative, given the small number of patients and samples analyzed for this section of the study, this data should be interpreted cautiously. An expanded study with substantially more temporal samples coupled with extensive medical histories for these patients is necessary to confirm that this observation holds true in larger populations over more protracted time-frames and if so, to determine the factors that drive this phenomenon e.g. frequency of antimicrobial administration.

**Figure 2:**
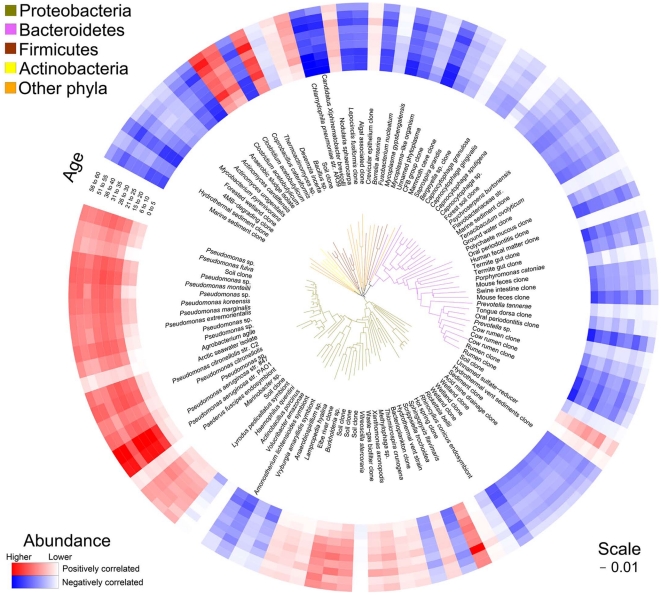
Phylogenetic tree displaying relationship between patient age and taxon abundance. Taxa exhibiting a significant increase (red) or decrease (blue) in relative abundance with increasing CF patient age are illustrated. Scale bar indicates 0.01 nucleotide substitutions per base.

These shifts in community structure and diversity observed in older CF patients may contribute to the phenomenon of age-specific airway pathogen abundance in CF patients [40], [41]. To examine these relationships in more detail, correlations between the abundance of all known CF airway pathogens and patient age were determined to identify CF pulmonary disease pathogens associated with pediatric and adult CF patients respectively. Of the known CF pathogens, only *Haemophilus influenzae* (p = 0.02, r = −0.31), *Stenotrophomonas maltophilia* (p = 0.02, r = 0.31) and *Pseudomonas aeruginosa* (p = 0.001, r = 0.42) exhibited significant correlations with age (Table 2). *P. aeruginosa* and *S. maltophilia* abundance was greatest in older CF patients (Fig. 2), who exhibited lower bacterial diversity. In contrast, *H. influenzae* exhibited the opposite trend with a peak in abundance in younger patients when community diversity was greatest (Fig. 2), observations, which are supported by previous culture-based reports [41], [42], [43].

**Table 2:** Correlation between pathogen abundance and increasing CF patient age.

*Pathogen*	*R*	*p-value*
Haemophilus influenzae	−0.31	0.02
Pseudomonas aeruginosa	0.42	0.001
Stenotrophomonas maltophilia (Xanthomonas axonopodis)	0.31	0.02

Shaded pathogens exhibit a significant correlation.

An advantage of the array is the ability to detect taxa not normally isolated by conventional culture. Thus the PhyloChip data was analyzed to identify other bacterial species that exhibited age-related changes in abundance, in an attempt to more broadly define those organisms associated with pediatric and adult CF airways. Following false discovery adjustment, a total of 113 taxa exhibited a significant correlation with age; 45 were positive correlations, 68 negative (a complete list is provided in Supplementary Table S3). Of the 45 taxa significantly positively correlated with age, almost half (22 taxa) were members of the Pseudomonadaceae (Fig. 2). This confirmed our previous observation that older CF patients possess airway communities that are less diverse and more phylogenetically related. Whether this phenomenon is a result of the Pseudomonadaceae actively defining a niche with less diversity or is due to their exploitation of an existing low diversity niche is unknown.

Many of the bacteria identified in this analysis have not yet been phenotyped, nor their potential for pathogenesis assessed. However, a number of known pathogens were more prevalent in younger CF patients, including members of the Mycobacteriaceae [44] and obligate intracellular members of the Chlamydiaceae [45], [46] and the Mycoplasmataceae [47] (Supplementary Table S3). In contrast, known or potential pathogens associated with older CF patients included members of the Burkholderiaceae and Thermoactinomycetaceae [48], [49]. Age-based relationships with anaerobic species were also examined since their relevance to CF airway disease is a subject of much recent research and discussion [50], [51]. Of the strict anaerobic bacteria that had a significant correlation with age, the majority exhibited a negative relationship and were detected in higher abundance in younger patients (Supplementary Table S3). However, a very small number of Clostridia and sulfate-reducing species exhibited an increase in relative abundance in older CF patients.

## Discussion

In this study, CF patient airway colonization was examined using a culture-independent phylogenetic microarray and samples from a cohort of patients defined as clinically stable (no change in pulmonary function for ≥2 months prior to sample collection), aged between 9 months and 72 years (Table S4). For the purpose of this study it was important to consider only clinically stable time points to determine the longer-term evolution of the CF airway microbiota and avoid the pronounced short-term impact of pulmonary exacerbation and antimicrobial therapy. Deep throat swabs were used to sample pediatric patients in this study, this was to ensure inclusion of neonatal and younger pediatric patients who do not produce sputum. Expectorated sputum samples (following routine spirometry) were collected for adult patients. Despite differences in sampling between the age groups, the mode of sampling did not seem to drive the bacterial community composition. One caveat of airway studies is the necessity to collect the samples through the oral cavity and hence the potential for sample contamination. Both deep throat swab and sputum samples are routinely used for clinical laboratory culture to guide antimicrobial treatment regimes for CF patients. In addition, Rogers and colleagues have previously demonstrated that distinct bacterial communities exists in oral and sputum CF samples [14], also supporting the hypothesis that oral contamination minimally impacts the assessment of airway samples from CF patients.

A total of 158 bacterial families were detected in these samples, the majority of taxa detected in these families have not been previously associated with cystic fibrosis. There are several potential explanations for this including that the PhyloChip is a highly sensitive molecular assay, capable of detecting organisms that are present at levels of only 0.01% of the total community. Validation by sequence analysis of *Veillonella parvula* confirms its reported presence in this niche by the array, suggesting that multiple organisms may co-habit this niche. Indeed, other culture-independent T-RFLP-based studies (estimated by the authors as having a sensitivity of 1% of the population), clone library sequencing and temporal temperature gradient gel electrophoresis (TTGE) have also detected a number of species that had not previously been associated with CF airways [10], [11], [15], [16]. Even with the limitations of culture-based methods, previous studies have also demonstrated the presence of uncharacteristic species in CF airways using this approach [52]. More recently a sequence-based study of a CF patient airway sample revealed the presence of multiple unusual species not previously reported in this niche such as *Dolosigranulosum pigrum, Kocuria rosea, Granulicatella* spp. and *Bergeyella* spp. amongst others [53]. All of the reported species detected in this sequence-based study were also detected by PhyloChip in several of the patients in our study. This in addition to our sequence-based validation, collectively suggest that a multitude of bacterial species do indeed exist in CF airway samples and underscore the need to perform truly deep sequencing to overcome the issue of the dominant species present and detect the “rare biosphere” present.

The impact of sequence depth on interpretation of results was recently demonstrated by Qin et al [54], in a large-scale sequence-based human microbiome study of 124 European individuals. Increasing the sequence depth coverage for samples from two individuals (from ∼4 Gb to >8.5 Gb) resulted in an increase in the number of strains common to these two individuals by 25% [54]. This demonstrates that human host microbiota are intrinsically diverse and that our current view of these assemblages is only curtailed by the limited depth of sequencing that has been used to interrogate them. Though we recognize that the array-based technology used in this study is potentially subject to cross-hybridization at the taxon-level, it nonetheless provides a standardized tool for high-resolution profiling of samples, permitting detection of low abundance species in dominated communities and relative changes in community composition that can be related to clinical measurements and features of the disease.

**Figure 3:**
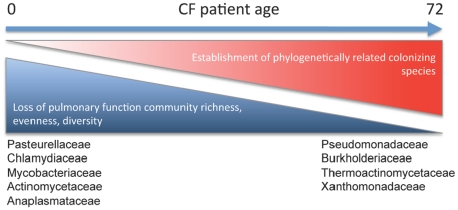
Bacterial community structure and composition associated with CF patient age. Compared to pediatric communities, adult CF patient airway communities exhibit lower bacterial diversity and are more uneven. Increased diversity in younger airways is correlated with a large number of known pathogenic families. Loss of diversity in older airways is strongly correlated with loss of pulmonary function and emergence of competitively dominant species such as members of the Pseudomonadacece, Burkholderiaceae and Xanthomonadaceae. For simplicity, the initial increase in diversity exhibited by younger CF patients is not illustrated.

In natural systems, colonizing populations are observed to develop progressively, typically leading to increased biomass, productivity and diversity [55], [56], [57], [58]. This has recently been exemplified in the human gastrointestinal tract; initial colonization by pioneer aerobic species is followed by facultative anaerobes and finally strict anaerobes, concomitant with an increase in bacterial biomass and productivity characteristic of the stable adult microbiota [59]. Though this is a cross-sectional study, data presented here provides insights into the progression of CF airway colonization. Evidence for initial diversification in younger patients, decreases in diversity in older patients and the presence of specialized communities of phylogenetically related species (Fig. 3) associated with poor pulmonary function in these older patient suggest that CF airway microbiota may also follow the rules of community assembly previously reported at other host niches. This form of colonization involving an initial rapid rise in species diversity as successive invasions occur, followed by species replacement as the community develops is common, and has previously been reported for other ecosystems and at higher trophic levels [60], [61], [62]. Once established, severe antimicrobial-based perturbation of microbial communities has been shown to lead to long-term changes in community composition and a loss of diversity in animal models [63]. Subsets of older CF patients exhibit diminished improvement in lung function in response to antimicrobial administration compared with younger CF patients [64], who also typically exhibit better pulmonary function. This suggests that the more phylogenetically-related microbial assemblages detected in older CF patients may be more resistant to these antimicrobials and contribute to poorer airway function, while the more diverse pediatric microbial assemblage is more sensitive to antimicrobial perturbation and linked to improved pulmonary function. More recently a study has demonstrated a “like begets like” phenomenon, in that colonization of a specific niche by particular keystone species results in “invasion” of that community by other phylogenetically related species [65]. It appears from our data that a similar scenario exists in older CF patients whose airway microbiota exhibits phylogenetically related members and is largely composed of Pseudomonadaceae. That multiple members of the Pseudomonadaceae may co-exist in CF airways is not unprecedented, Harris and colleagues have previously demonstrated with relatively shallow sequencing depth, the presence of other *Pseudomonas* species other than *P. aeruginosa,* in 50% of their CF patient airways [10], suggesting that multiple members of the Pseudomonadaecaeae may co-exist in this niche.

It seems counter-intuitive that a greater diversity of organisms (in younger CF patients) would be associated with better lung function, however several studies have demonstrated that dramatic changes in community structure through loss of diversity as reported here, are increasingly being associated with chronic inflammatory diseases, pathogen outgrowth and poor clinical outcome [3], [66], [67], [68], [69]. It appears therefore, that bacterial community structure and composition represents an important factor in defining the functionality of the microbial assemblage and host health status. However analysis of CF cohorts is difficult and confounded by the fact that a number of the older patients in this study have less severe disease, which may be associated with their microbial community composition or different treatment regimens. In addition, there is also the possibility that the findings reported here are due to other factors that track with age such as antibiotic use, chest physical therapy, adherence, nutrition or other factors. Nonetheless, this study demonstrates that distinct microbial assemblages are associated with CF patient age and that community composition is correlated with CF genotype and aspects of pulmonary disease in this patient population.

This study also provided information on the relationship between CFTR mutation and the airway microbiota. It has previously been reported that CFTR mutation changes the airway microenvironment [70], [71], [72], which would presumably influence the microbial community that establishes in this niche. A strong relationship between ΔF508 CFTR mutation and absence of multiple members of the Mycobacteriaceae, Staphylococcaceae, Enterobacteriaceae amongst others with a concomitant rise in abundance of members of the Pseudomonadaceae was identified in older CF patients with this genotype. This suggests that mutations rendering the CFTR non-functional are associated with a loss of airway bacterial (including pathogen) diversity and outgrowth of a small group of phylogenetically-related species as these patients age. Whether this is directly related to the severity of the mutation and the creation of a distinct niche due to lack of functional CFTR or due to the treatments necessary to manage these patients (or a combination of both) is unclear. However, patients with heterozygous ΔF508 or non-ΔF508 mutations also exhibited distinct pathogen profiles. Older heterozygous ΔF508CF patient airways were associated with Moraxellaceaea and Sphingobacteriaceae, two bacterial families that have recently been associated with chronic obstructive pulmonary disease [73] and invariant Natural Killer T cell induction in asthmatic mice [74]. While non-ΔF508 patients were associated with a relatively less severe pathogen profile. This study, albeit small, demonstrates that homozygous-ΔF508 mutation is associated with the most substantial change in airway community structure and phylogeny in older patients. Furthermore, particular CFTR mutations, which are known to influence the airway environment [70], [71], [72], are associated with distinct pathogen profiles, a finding that may explain the range of severity in pulmonary symptoms commonly observed with various CFTR genotypes, and has implications for patient-tailored care [75].

It is important to note that detailed functional analyses of longitudinal CF airway samples are necessary to comprehensively determine the impact of treatment on the airway microbiota. Given our data, it appears that older CF patients possess a stable core of pathogenic organisms which presumably are selected for over time due to repeated antimicrobial pressure. With the increasing lifespan of CF patients due to successful disease management, understanding the long-term impact of antimicrobials on the CF microbiota may lead to further improvements in treatment strategies and life expectancy. Novel approaches that involve manipulation of microbial consortia, rather than destruction of the community structure may offer an alternative therapeutic approach for patient-tailored management of chronic airway disease. This concept is gaining increasing support [8], [63] and there is evidence for its efficacy, although the mechanism underlying these benefits remains unclear. A recent pilot study of pediatric CF patients supplemented with a probiotic *Lactobacillus* species or placebo demonstrated that in addition to reduced gastrointestinal inflammation, patients who received the probiotic species exhibited a significant reduction in hospitalizations for pulmonary exacerbation [76]. Certainly with the advent of newborn screening programs and sophisticated culture-independent tools to comprehensively monitor patient airways, the opportunity to intervene at a very early age and alter the course of airway microbial colonization to improve patient outcome is unprecedented.

The CF microbiota is a complex community, but the use of molecular ecological approaches permits the progression of CF airway disease to be comprehensively explored. This work provides the foundation for an improved understanding of polymicrobial CF airway colonization, the relationship between community structure, composition and lung function in CF patients and the influence of CFTR mutation on the airway microbiota. Antibiotic administration, the acquisition of organisms from the internal and external environments, and changes associated with patient age (increased lung surface area, hormonal changes) represent key influences on this ecosystem. Understanding the mechanistic relationship between community dynamics, pathogen abundance and behavior, and the host immune response is crucial to further extending the lifespan of this patient population.

## Supporting Information

Table S1 Taxa identified by 16S rRNA PhyloChip in the airways of CF patients.
(2.39 MB DOC)

Table S2 Correlation of taxon abundance and CFTR mutation.
(0.23 MB XLS)

Table S3 Correlation of taxon abundance with patient age.
(0.05 MB XLS)

Table S4 Patient demographics.
(0.09 MB DOC)

Figure S1 Change in bacterial community diversity over time. Change in diversity, normalized to length of time between sample collection points was calculated for CF patients ranging in age from 9 months to 43 years old and illustrates an initial net increase in diversity (per day) in younger patients in comparison with a decrease in diversity in older patients.
(0.18 MB TIF)
