# Supplementary material for: Airway Microbiota and Pathogen Abundance in Age-Stratified Cystic Fibrosis Patients
Source: PLoS One. 2010 Jun 23;5(6):e11044. doi: 10.1371/journal.pone.0011044 (PMC2890402; doi:10.1371/journal.pone.0011044)
Supplement: Table S1 — Taxa identified by 16S rRNA PhyloChip in the airways of CF patients. (2.39 MB DOC) [file pone.0011044.s001.doc]

| **Domain** | **Phylum** | **Class** | **Order** | **Family** | **S-F^a^** | **TID^b^** | **Accession #** | **Representative species^c^** |
| --- | --- | --- | --- | --- | --- | --- | --- | --- |
| Bacteria | OP10 | Unclassified | Unclassified | Unclassified | sf_4 | 728 | AY214187.1 | benzene-contaminated groundwater clone ZZ12AC2 |
| Bacteria | Nitrospira | Nitrospira | Nitrospirales | Nitrospiraceae | sf_3 | 240 | AF543501.1 | acid mine drainage clone AW4 |
| Bacteria | Nitrospira | Nitrospira | Nitrospirales | Nitrospiraceae | sf_2 | 544 | AF524004.1 | forested wetland clone FW5 |
| Bacteria | Verrucomicrobia | Verrucomicrobiae | Verrucomicrobiales | Verrucomicrobia subdivision 5 | sf_1 | 111 | AF507896.1 | Mono Lake clone ML635J-35 |
| Bacteria | Verrucomicrobia | Verrucomicrobiae | Verrucomicrobiales | Verrucomicrobiaceae | sf_7 | 29 | AB073978.1 | Fucophilus fucoidanolyticus str. SI-1234 |
| Bacteria | Synergistes | Unclassified | Unclassified | Unclassified | sf_3 | 248 | U81706.2 | anaerobic digestor clone vadinCA02 |
| Bacteria | Termite group 1 | Unclassified | Unclassified | Unclassified | sf_2 | 437 | AB089050.2 | termite gut homogenate clone Rs-D43 group |
| Bacteria | Termite group 1 | Unclassified | Unclassified | Unclassified | sf_2 | 722 | AB089051.1 | termite gut homogenate clone Rs-H93 group |
| Bacteria | Acidobacteria | Acidobacteria-7 | Unclassified | Unclassified | sf_1 | 588 | AJ009461.1 | TCB-transforming consortium clone SJA-36 |
| Bacteria | Acidobacteria | Acidobacteria | Acidobacteriales | Acidobacteriaceae | sf_14 | 176 | AJ519387.1 | uranium mining waste pile clone JG37-AG-112 sp. |
| Bacteria | Acidobacteria | Acidobacteria | Acidobacteriales | Acidobacteriaceae | sf_14 | 274 | AJ519379.1 | uranium mining waste pile clone JG37-AG-73 sp. |
| Bacteria | Acidobacteria | Acidobacteria | Acidobacteriales | Acidobacteriaceae | sf_14 | 582 | AY102321.1 | heavy metal-contaminated soil clone a13114 |
| Bacteria | Acidobacteria | Acidobacteria | Acidobacteriales | Acidobacteriaceae | sf_14 | 994 | AJ519389.1 | uranium mining waste pile clone JG37-AG-117 sp. |
| Bacteria | Bacteroidetes | Bacteroidetes | Bacteroidales | Porphyromonadaceae | sf_1 | 5557 | AY008308.1 | Bacteroides cf. forsythus oral clone BU063 forsythus |
| Bacteria | Bacteroidetes | Bacteroidetes | Bacteroidales | Porphyromonadaceae | sf_1 | 5652 | AB088943.1 | termite gut homogenate clone Rs-D29 bacterium |
| Bacteria | Bacteroidetes | Bacteroidetes | Bacteroidales | Porphyromonadaceae | sf_1 | 6214 | AB035460.1 | Tannerella forsythensis str. ATCC43037 |
| Bacteria | Bacteroidetes | Bacteroidetes | Bacteroidales | Prevotellaceae | sf_1 | 5768 | AF385564.1 | crevicular epithelial cells clone BU035 |
| Bacteria | Bacteroidetes | Bacteroidetes | Bacteroidales | Bacteroidaceae | sf_12 | 5837 | AF132263.1 | adult human fecal matter clone adhufec355 |
| Bacteria | Bacteroidetes | Bacteroidetes | Bacteroidales | Prevotellaceae | sf_1 | 5705 |  | |
| Bacteria | Bacteroidetes | Bacteroidetes | Bacteroidales | Prevotellaceae | sf_1 | 5756 | AY689226.1 | Prevotella intermedia str. ChDC KB53 |
| Bacteria | Bacteroidetes | Bacteroidetes | Bacteroidales | Prevotellaceae | sf_1 | 6207 | AY244912.1 | cow rumen clone BF34 |
| Bacteria | Bacteroidetes | Bacteroidetes | Bacteroidales | Unclassified | sf_15 | 5257 | AY188307.1 | marine? clone KD3-67 |
| Bacteria | Bacteroidetes | Sphingobacteria | Sphingobacteriales | Unclassified | sf_4 | 6030 | AF445648.1 | travertine hot spring clone SM1A07 |
| Bacteria | Chloroflexi | Anaerolineae | Chloroflexi-1b | Unclassified | sf_1 | 1071 | AJ412677.1 | denitrifying reactor clone 131 |
| Bacteria | Chloroflexi | Anaerolineae | Unclassified | Unclassified | sf_9 | 94 | AJ306740.1 | DCP-dechlorinating consortium clone SHA-2 |
| Bacteria | Planctomycetes | Planctomycetacia | WPS-1 | Unclassified | sf_1 | 4895 | AY093482.1 | deep marine sediment clone MB-C2-147 |
| Bacteria | Planctomycetes | Planctomycetacia | WPS-1 | Unclassified | sf_1 | 4897 | AY093476.1 | deep marine sediment clone MB-C2-105 |
| Bacteria | Cyanobacteria | Unclassified | Unclassified | Unclassified | sf_5 | 5131 | AB045961.1 | Oscillatoria princeps str. NIVA CYA 150 |
| Bacteria | Cyanobacteria | Cyanobacteria | Oscillatoriales | Unclassified | sf_1 | 5172 | AB058249.1 | Cyanobacterium sp. str. MBIC10216 |
| Bacteria | Cyanobacteria | Cyanobacteria | Chloroplasts | Chloroplasts | sf_5 | 4984 | U70721.1 | Cape Hatteras picoplankton clone OM164 |
| Bacteria | Cyanobacteria | Cyanobacteria | Chloroplasts | Chloroplasts | sf_5 | 5039 | U70723.1 | Cape Hatteras picoplankton clone OM270 |
| Bacteria | Cyanobacteria | Cyanobacteria | Chloroplasts | Chloroplasts | sf_5 | 5166 |  | |
| Bacteria | Cyanobacteria | Cyanobacteria | Chloroplasts | Chloroplasts | sf_5 | 5112 | X52985.1 | Cyanidium caldarium str. 14-1-1 |
| Bacteria | Unclassified | Unclassified | Unclassified | Unclassified | sf_160 | 6339 |  | |
| Bacteria | Acidobacteria | Acidobacteria | Acidobacteriales | Acidobacteriaceae | sf_14 | 6407 |  | |
| Bacteria | Acidobacteria | Acidobacteria | Acidobacteriales | Acidobacteriaceae | sf_14 | 6405 | AJ534632.1 | soil sample uranium mining waste pile near town Johanngeorgenstadt clone JG36-TzT-31 bacterium |
| Bacteria | Spirochaetes | Spirochaetes | Spirochaetales | Spirochaetaceae | sf_1 | 6547 | AF507855.1 | Mono Lake at depth 23 m station 6 July 2000 clone ML623J-23 bacterium |
| Bacteria | Spirochaetes | Spirochaetes | Spirochaetales | Spirochaetaceae | sf_1 | 6573 | AB084955.1 | termite gut clone NkS7 |
| Bacteria | Proteobacteria | Alphaproteobacteria | Acetobacterales | Roseococcaceae | sf_1 | 6783 | AY150048.1 | Roseomonas genomospecies 4 strain isolate str. ATCC 49959 4 |
| Bacteria | Proteobacteria | Alphaproteobacteria | Acetobacterales | Acetobacteraceae | sf_1 | 7600 | AJ001632.1 | Acetobacter pomorum str. LTH2458 |
| Bacteria | Proteobacteria | Alphaproteobacteria | Verorhodospirilla | Unclassified | sf_1 | 7109 | AY189753.1 | diesel-polluted Bohai Gulf isolate str. M-5 M-5 |
| Bacteria | Proteobacteria | Alphaproteobacteria | Bradyrhizobiales | Beijerinck/Rhodoplan/Methylocyst | sf_3 | 7401 | AF359545.1 | Scrippsiella trochoidea NEPCC 15 |
| Bacteria | Proteobacteria | Alphaproteobacteria | Azospirillales | Azospirillaceae | sf_1 | 6959 | AF523824.1 | Rhodocista pekingensis str. 3-p |
| Bacteria | Proteobacteria | Alphaproteobacteria | Azospirillales | Azospirillaceae | sf_1 | 7171 | Z29623.1 | Azospirillum species |
| Bacteria | Proteobacteria | Alphaproteobacteria | Azospirillales | Azospirillaceae | sf_1 | 7539 | AJ401217.1 | Rhodocista sp. AR2107 |
| Bacteria | Proteobacteria | Alphaproteobacteria | Rhizobiales | Unclassified | sf_1 | 7339 |  | |
| Bacteria | Proteobacteria | Alphaproteobacteria | Bradyrhizobiales | Beijerinck/Rhodoplan/Methylocyst | sf_3 | 7219 | M95665.1 | Methylosinus sporium |
| Bacteria | Proteobacteria | Alphaproteobacteria | Bradyrhizobiales | Beijerinck/Rhodoplan/Methylocyst | sf_3 | 7640 | AF150804.1 | Methylosinus trichosporium |
| Bacteria | Proteobacteria | Alphaproteobacteria | Bradyrhizobiales | Beijerinck/Rhodoplan/Methylocyst | sf_3 | 7153 | AJ563928.1 | Methylocella tundrae str. Y1 |
| Bacteria | Proteobacteria | Alphaproteobacteria | Bradyrhizobiales | Bradyrhizobiaceae | sf_1 | 7403 | AB099659.1 | Oligotropha carboxidovorans str. S23 |
| Bacteria | Proteobacteria | Alphaproteobacteria | Bradyrhizobiales | Bradyrhizobiaceae | sf_1 | 6927 | L11663.1 | Nitrobacter hamburgensis str. X14 |
| Bacteria | Proteobacteria | Alphaproteobacteria | Bradyrhizobiales | Bradyrhizobiaceae | sf_1 | 6941 | AB087719.1 | Rhodopseudomonas rhenobacensis str. Klemme Rb |
| Bacteria | Proteobacteria | Alphaproteobacteria | Bradyrhizobiales | Bradyrhizobiaceae | sf_1 | 6867 | AY102327.1 | heavy metal-contaminated soil clone a13131 |
| Bacteria | Proteobacteria | Alphaproteobacteria | Bradyrhizobiales | Bradyrhizobiaceae | sf_1 | 7044 | U87765.1 | Afipia genosp. 2 str. G4438 |
| Bacteria | Proteobacteria | Alphaproteobacteria | Bradyrhizobiales | Bradyrhizobiaceae | sf_1 | 6917 | D11345.1 | Bradyrhizobium japonicum str. IAM 12608 |
| Bacteria | Proteobacteria | Alphaproteobacteria | Rhizobiales | Unclassified | sf_1 | 7039 |  | |
| Bacteria | Proteobacteria | Alphaproteobacteria | Rhizobiales | Rhizobiaceae | sf_1 | 6683 | D14516.1 | Sinorhizobium fredii str. ATCC35423 |
| Bacteria | Proteobacteria | Alphaproteobacteria | Rhizobiales | Rhizobiaceae | sf_1 | 6770 | X67234.2 | Rhizobium tropici str. LMG 9517 |
| Bacteria | Proteobacteria | Alphaproteobacteria | Unclassified | Unclassified | sf_6 | 7229 | AF529343.1 | PCE-contaminated site clone CLi8 |
| Bacteria | Proteobacteria | Alphaproteobacteria | Caulobacterales | Caulobacteraceae | sf_1 | 7257 | AJ459874.1 | larval intestine clone D |
| Bacteria | Proteobacteria | Alphaproteobacteria | Rhodobacterales | Rhodobacteraceae | sf_1 | 7453 | AJ534205.1 | Sulfitobacter sp. BIO-11 |
| Bacteria | Proteobacteria | Alphaproteobacteria | Consistiales | SAR11 | sf_2 | 7043 | AF353223.1 | marine clone Arctic95D-8 |
| Bacteria | Proteobacteria | Alphaproteobacteria | Sphingomonadales | Sphingomonadaceae | sf_1 | 7532 | AF115500.1 | Caulobacter sp. str. WCP2020S |
| Bacteria | Proteobacteria | Alphaproteobacteria | Sphingomonadales | Sphingomonadaceae | sf_1 | 6690 | AY048657.1 | Porphyrobacter tepidarius str. OK5APO |
| Bacteria | Proteobacteria | Betaproteobacteria | Neisseriales | Neisseriaceae | sf_1 | 7885 | M22517.1 | Kingella kingae str. ATCC 23330 |
| Bacteria | Proteobacteria | Betaproteobacteria | Nitrosomonadales | Nitrosomonadaceae | sf_1 | 7805 | AY123800.1 | Nitrosospira briensis str. Nsp10 |
| Bacteria | Proteobacteria | Betaproteobacteria | Nitrosomonadales | Nitrosomonadaceae | sf_1 | 7858 |  | |
| Bacteria | Proteobacteria | Betaproteobacteria | Nitrosomonadales | Nitrosomonadaceae | sf_1 | 7976 | AY123798.1 | Nitrosomonas sp. str. Nm86 |
| Bacteria | Proteobacteria | Betaproteobacteria | Nitrosomonadales | Nitrosomonadaceae | sf_1 | 7989 | AY123811.1 | Nitrosomonas sp. str. Nm59 |
| Bacteria | Proteobacteria | Betaproteobacteria | Burkholderiales | Comamonadaceae | sf_1 | 7718 | AY291119.1 | Lampropedia hyalina str. DSM 15336 |
| Bacteria | Proteobacteria | Betaproteobacteria | Burkholderiales | Comamonadaceae | sf_1 | 8021 | AF078765.1 | Acidovorax facilis str. CCUG 2113 |
| Bacteria | Proteobacteria | Betaproteobacteria | Burkholderiales | Comamonadaceae | sf_1 | 8031 | AB021328.1 | strain isolate str. rJ10 |
| Bacteria | Proteobacteria | Betaproteobacteria | Burkholderiales | Comamonadaceae | sf_1 | 8046 | Y18616.1 | Acidovorax defluvii str. BSB411 |
| Bacteria | Proteobacteria | Betaproteobacteria | Burkholderiales | Comamonadaceae | sf_1 | 7871 |  | |
| Bacteria | Proteobacteria | Betaproteobacteria | Burkholderiales | Comamonadaceae | sf_1 | 7943 | AF035052.1 | Aquabacterium parvum str. B6 |
| Bacteria | Proteobacteria | Betaproteobacteria | Burkholderiales | Burkholderiaceae | sf_1 | 7918 | U16140.1 | Burkholderia sp str. GSOY |
| Bacteria | Proteobacteria | Gammaproteobacteria | Unclassified | Unclassified | sf_3 | 8874 | AF420367.1 | hydrothermal sediment clone AF420367 |
| Bacteria | Proteobacteria | Gammaproteobacteria | Unclassified | Unclassified | sf_3 | 9394 |  | |
| Bacteria | Proteobacteria | Gammaproteobacteria | Oceanospirillales | Unclassified | sf_3 | 8961 | M99446.1 | Calyptogena magnifica symbiont |
| Bacteria | Proteobacteria | Gammaproteobacteria | SUP05 | Unclassified | sf_1 | 8965 | M99445.1 | Bathymodiolus thermophilus gill symbiont |
| Bacteria | Proteobacteria | Gammaproteobacteria | Xanthomonadales | Xanthomonadaceae | sf_3 | 8682 | AB021391.1 | Pseudomonas boreopolis str. ATCC 33662T |
| Bacteria | Proteobacteria | Gammaproteobacteria | Legionellales | Legionellaceae | sf_1 | 9617 | X73406.1 | Legionella feeleii subsp. sgp 2 str. ATCC 35849 |
| Bacteria | Proteobacteria | Gammaproteobacteria | Unclassified | Unclassified | sf_3 | 8668 |  | |
| Bacteria | Proteobacteria | Gammaproteobacteria | GAO cluster | Unclassified | sf_1 | 8980 | AY098909.1 | activated sludge clone SBRL2_19 |
| Bacteria | Proteobacteria | Gammaproteobacteria | Methylococcales | Methylococcaceae | sf_1 | 8645 | AF307138.1 | Methylomicrobium buryatense str. 5B |
| Bacteria | Proteobacteria | Gammaproteobacteria | Methylococcales | Unclassified | sf_1 | 9182 | AF304197.1 | Methylobacter marinus str. A45 |
| Bacteria | Proteobacteria | Gammaproteobacteria | Unclassified | Unclassified | sf_3 | 9269 |  | |
| Bacteria | Proteobacteria | Gammaproteobacteria | Oceanospirillales | Halomonadaceae | sf_1 | 8317 | AF211861.1 | Chromohalobacter canadensis str. DSM6769 |
| Bacteria | Proteobacteria | Gammaproteobacteria | Alteromonadales | Alteromonadaceae | sf_1 | 9035 | AB124836.1 | Microbulbifer sp. str. JAMB-A94 |
| Bacteria | Proteobacteria | Gammaproteobacteria | Alteromonadales | Alteromonadaceae | sf_1 | 8348 | AF468388.1 | Arctic sea ice ARK10038 |
| Bacteria | Proteobacteria | Gammaproteobacteria | Pseudomonadales | Moraxellaceae | sf_3 | 9359 | AB101444.1 | Acinetobacter junii str. S33 |
| Bacteria | Proteobacteria | Gammaproteobacteria | Pseudomonadales | Pseudomonadaceae | sf_1 | 8635 |  | |
| Bacteria | Proteobacteria | Gammaproteobacteria | Unclassified | Unclassified | sf_3 | 8883 |  | |
| Bacteria | Proteobacteria | Gammaproteobacteria | Alteromonadales | Alteromonadaceae | sf_1 | 8503 | AF468401.1 | Arctic sea ice ARK10244 |
| Bacteria | Proteobacteria | Gammaproteobacteria | Alteromonadales | Alteromonadaceae | sf_1 | 8167 | AF359542.1 | Alexandrium tamarense PCC 173a |
| Bacteria | Proteobacteria | Gammaproteobacteria | Alteromonadales | Alteromonadaceae | sf_1 | 9230 | AY165576.1 | Antarctic pack ice Lasarev Sea Southern Ocean clone ANTXI/4_14-62 sea |
| Bacteria | Proteobacteria | Gammaproteobacteria | Alteromonadales | Shewanellaceae | sf_1 | 8581 | AB008796.1 | Shewanella benthica str. DB21MT-2 |
| Bacteria | Proteobacteria | Gammaproteobacteria | Alteromonadales | Alteromonadaceae | sf_1 | 9011 |  | |
| Bacteria | Proteobacteria | Gammaproteobacteria | Alteromonadales | Alteromonadaceae | sf_1 | 8916 | U91545.1 | Shewanella algae str. 43940 |
| Bacteria | Proteobacteria | Gammaproteobacteria | Vibrionales | Vibrionaceae | sf_1 | 9174 |  | |
| Bacteria | Proteobacteria | Gammaproteobacteria | Enterobacteriales | Enterobacteriaceae | sf_1 | 8758 | U80201.1 | Pectobacterium cypripedii str. ATCC 29267 |
| Bacteria | Proteobacteria | Gammaproteobacteria | Enterobacteriales | Enterobacteriaceae | sf_1 | 8801 | X92550.1 | Candidatus camponotii |
| Bacteria | Proteobacteria | Gammaproteobacteria | Enterobacteriales | Enterobacteriaceae | sf_1 | 8505 | AJ233406.1 | Buttiauxella warmboldiae str. DSM 9404 |
| Bacteria | Proteobacteria | Gammaproteobacteria | Enterobacteriales | Enterobacteriaceae | sf_1 | 8251 | AF214640.1 | Nitrogen-fixing isolate str. CANF3 |
| Bacteria | Proteobacteria | Gammaproteobacteria | Enterobacteriales | Enterobacteriaceae | sf_1 | 8712 | AJ233434.1 | Serratia proteamaculans str. DSM 4543 |
| Bacteria | Proteobacteria | Gammaproteobacteria | Enterobacteriales | Enterobacteriaceae | sf_1 | 9417 | AJ233429.1 | Serratia fonticola str. DSM 4576 |
| Bacteria | Proteobacteria | Deltaproteobacteria | Syntrophobacterales | Syntrophobacteraceae | sf_1 | 10184 | AF482435.1 | granular sludge clone R1p32 |
| Bacteria | Proteobacteria | Deltaproteobacteria | Syntrophobacterales | Syntrophobacteraceae | sf_1 | 10294 | AF170417.1 | Desulfacinum hydrothermale str. MT-96 |
| Bacteria | Proteobacteria | Deltaproteobacteria | Desulfobacterales | Desulfobulbaceae | sf_1 | 9705 | AJ535237.1 | marine sediment above hydrate ridge clone Hyd01-n proteobacterium |
| Bacteria | Proteobacteria | Epsilonproteobacteria | Campylobacterales | Campylobacteraceae | sf_3 | 10474 | AB013833.1 | NB1-l clone NB1-l |
| Bacteria | Fusobacteria | Fusobacteria | Fusobacterales | Fusobacteriaceae | sf_3 | 1057 | AY029805.1 | Leptotrichia trevisanii str. LB06 |
| Bacteria | Fusobacteria | Fusobacteria | Fusobacterales | Fusobacteriaceae | sf_3 | 367 | AY078425.1 | Leptotrichia amnionii str. AMN-1 |
| Bacteria | Gemmatimonadetes | Unclassified | Unclassified | Unclassified | sf_5 | 1149 | AF432618.1 | lodgepole pine rhizosphere soil clone C17.11WL |
| Bacteria | Actinobacteria | Actinobacteria | Coriobacteriales | Coriobacteriaceae | sf_1 | 2021 | AB089070.1 | termite gut homogenate clone Rs-D41 bacterium |
| Bacteria | Actinobacteria | Actinobacteria | Actinomycetales | Streptomycetaceae | sf_1 | 1690 | X79325.1 | Streptomyces galbus str. DSM40480 |
| Bacteria | Actinobacteria | Actinobacteria | Actinomycetales | Thermomonosporaceae | sf_1 | 1406 | X97890.1 | Actinomadura kijaniata str. DSM 43764T |
| Bacteria | Actinobacteria | Actinobacteria | Actinomycetales | Thermomonosporaceae | sf_1 | 1669 | AF223348.1 | Pseudonocardiaceae str. PA123 |
| Bacteria | Actinobacteria | Actinobacteria | Actinomycetales | Thermomonosporaceae | sf_1 | 2043 | AY035998.1 | Actinomadura latina str. DSM 43382 |
| Bacteria | Actinobacteria | Actinobacteria | Actinomycetales | Streptosporangiaceae | sf_1 | 1189 | AF191734.1 | Streptosporangium subroseum str. 7113 |
| Bacteria | Actinobacteria | Actinobacteria | Actinomycetales | Microbacteriaceae | sf_1 | 1345 | AJ781047.1 | Leucobacter aridicollis str. L9 |
| Bacteria | Actinobacteria | Actinobacteria | Actinomycetales | Microbacteriaceae | sf_1 | 1513 | AF263565.1 | Microbacterium gubbeenense str. DPC 5284 |
| Bacteria | Actinobacteria | Actinobacteria | Actinomycetales | Dermatophilaceae | sf_1 | 1216 | AY218605.1 | penguin droppings sediments clone KD3-138 |
| Bacteria | Actinobacteria | Actinobacteria | Actinomycetales | Actinomycetaceae | sf_1 | 1883 | AJ404889.1 | Actinomyces funkei str. CCUG 42773 |
| Bacteria | Actinobacteria | Actinobacteria | Actinomycetales | Propionibacteriaceae | sf_1 | 2002 | AB108484.1 | Propionibacterium acnes #2929 |
| Bacteria | Actinobacteria | Actinobacteria | Actinomycetales | Nocardioidaceae | sf_1 | 1854 | AY166703.1 | Aeromicrobium marinum str. T2 |
| Bacteria | Actinobacteria | Actinobacteria | Actinomycetales | Micromonosporaceae | sf_1 | 1876 | D14645.1 | Couchioplanes subsp. caeruleus str. IFO13939 |
| Bacteria | Actinobacteria | Actinobacteria | Actinomycetales | Actinosynnemataceae | sf_1 | 1463 | AF114815.1 | Saccharothrix texasensis str. NRRL B-16107T |
| Bacteria | Actinobacteria | Actinobacteria | Actinomycetales | Pseudonocardiaceae | sf_1 | 1119 | AJ508241.1 | Amycolatopsis tolypomycina str. DSM 44544 |
| Bacteria | Actinobacteria | Actinobacteria | Actinomycetales | Mycobacteriaceae | sf_1 | 1637 | AF190800.1 | Mycobacterium austroafricanum str. IFP2173 |
| Bacteria | Actinobacteria | Actinobacteria | Actinomycetales | Corynebacteriaceae | sf_1 | 1338 | X84258.1 | Corynebacterium pseudodiphtheriticum str. NCTC 11136 |
| Bacteria | OP9/JS1 | JS1 | Unclassified | Unclassified | sf_1 | 2380 | AY197406.1 | Guaymas Basin hydrothermal vent sediments clone B03R012 |
| Bacteria | Chloroflexi | Anaerolineae | Unclassified | Unclassified | sf_9 | 2539 | AY218649.1 | penguin droppings sediments clone KD4-96 |
| Bacteria | Firmicutes | Clostridia | Clostridiales | Clostridiaceae | sf_12 | 132 | AB089019.1 | termite gut homogenate clone Rs-J36 bacterium |
| Bacteria | Firmicutes | Clostridia | Clostridiales | Clostridiaceae | sf_12 | 206 | AB089017.1 | termite gut homogenate clone Rs-P50 bacterium |
| Bacteria | Firmicutes | Clostridia | Clostridiales | Lachnospiraceae | sf_5 | 2808 | AJ508452.1 | Clostridium bolteae str. 16351 |
| Bacteria | Firmicutes | Clostridia | Clostridiales | Lachnospiraceae | sf_5 | 2928 | AF349417.1 | pig feces clone |
| Bacteria | Firmicutes | Clostridia | Clostridiales | Lachnospiraceae | sf_5 | 2971 | X87152.1 | Johnsonella ignava str. ATCC 51276 |
| Bacteria | Firmicutes | Clostridia | Clostridiales | Clostridiaceae | sf_12 | 3004 | AB023973.1 | Clostridium hylemonae str. TN-272 |
| Bacteria | Firmicutes | Clostridia | Clostridiales | Lachnospiraceae | sf_5 | 2815 | AF399956.1 | Shuttleworthia satelles str. VPI D143K-13 |
| Bacteria | Firmicutes | Clostridia | Clostridiales | Lachnospiraceae | sf_5 | 3033 | AJ408968.1 | human colonic clone HuCA15 |
| Bacteria | Firmicutes | Catabacter | Unclassified | Unclassified | sf_4 | 3023 | AY197417.1 | Guaymas Basin hydrothermal vent sediments clone B04R016 |
| Bacteria | TM7 | TM7-3 | Unclassified | Unclassified | sf_1 | 2917 | AF507686.1 | arid soil clone C026 |
| Bacteria | Firmicutes | Clostridia | Clostridiales | Peptostreptococcaceae | sf_5 | 668 | AY221992.1 | Sedimentibacter sp. str. BRS2 |
| Bacteria | Firmicutes | Clostridia | Clostridiales | Peptostreptococcaceae | sf_5 | 17 | AF538859.1 | Peptostreptococcus sp. oral clone P4PA_156 P4 oral |
| Bacteria | Firmicutes | Clostridia | Clostridiales | Peptostreptococcaceae | sf_5 | 919 | AF542234.1 | Anaerococcus tetradius str. CCUG 46590 |
| Bacteria | Firmicutes | Bacilli | Bacillales | Paenibacillaceae | sf_1 | 3651 | AJ011323.1 | Paenibacillus borealis KK20 |
| Bacteria | Firmicutes | Bacilli | Bacillales | Paenibacillaceae | sf_1 | 319 | Y14579.1 | Ammoniphilus oxalaticus str. RAOx-FF |
| Bacteria | Firmicutes | Bacilli | Bacillales | Paenibacillaceae | sf_1 | 625 | Y14580.1 | Ammoniphilus oxalivorans str. RAOx-FS |
| Bacteria | Firmicutes | Bacilli | Bacillales | Halobacillaceae | sf_1 | 3702 | AJ496807.1 | Amphibacillus xylanus str. DSM 6626 |
| Bacteria | Firmicutes | Bacilli | Bacillales | Halobacillaceae | sf_1 | 3758 | AY121435.1 | Amphibacillus sp. str. YIM-kkny10 |
| Bacteria | Firmicutes | Bacilli | Bacillales | Bacillaceae | sf_1 | 3375 | AF423230.1 | soil clone 1448-1 |
| Bacteria | Firmicutes | Bacilli | Bacillales | Staphylococcaceae | sf_1 | 3585 |  | |
| Bacteria | Firmicutes | Bacilli | Bacillales | Staphylococcaceae | sf_1 | 3724 | Y13365.1 | Gemella bergeri str. 617-93 |
| Bacteria | Firmicutes | Bacilli | Bacillales | Bacillaceae | sf_1 | 3784 | AF423297.1 | soil clone 889-1 |
| Bacteria | Firmicutes | Bacilli | Bacillales | Bacillaceae | sf_1 | 3482 | AB116126.1 | garbage compost isolate str. M32 |
| Bacteria | Firmicutes | Bacilli | Bacillales | Bacillaceae | sf_1 | 3542 | AJ277984.1 | Bacillus psychrodurans str. DSM 11713 68E3 |
| Bacteria | Firmicutes | Bacilli | Lactobacillales | Aerococcaceae | sf_1 | 3615 | AJ278341.2 | Aerococcus urinaehominis str. CCUG 42038b |
| Bacteria | Firmicutes | Bacilli | Lactobacillales | Leuconostocaceae | sf_1 | 3311 | M23035.1 | Leuconostoc mesenteroides |
| Bacteria | Firmicutes | Bacilli | Lactobacillales | Lactobacillaceae | sf_1 | 3604 |  | |
| Bacteria | Firmicutes | Bacilli | Lactobacillales | Lactobacillaceae | sf_1 | 3798 | AY373589.1 | Lactobacillus fermentum str. MD-9 |
| Bacteria | Firmicutes | Bacilli | Lactobacillales | Lactobacillaceae | sf_1 | 3281 | AF371490.1 | swine intestine clone p-2984-9F2 |
| Bacteria | Firmicutes | Bacilli | Lactobacillales | Lactobacillaceae | sf_1 | 3874 | AB018212.1 | Pediococcus acidilactici str. E 7 |
| Bacteria | Firmicutes | Bacilli | Lactobacillales | Aerococcaceae | sf_1 | 3682 | X70907.1 | Dolosigranulum pigrum str. NCFB 2975 |
| Bacteria | Firmicutes | Bacilli | Lactobacillales | Aerococcaceae | sf_1 | 3504 | AB083413.1 | Marinilactibacillus psychrotolerans str. O21 |
| Bacteria | Firmicutes | Bacilli | Lactobacillales | Carnobacteriaceae | sf_1 | 3812 |  | |
| Bacteria | Firmicutes | Bacilli | Lactobacillales | Streptococcaceae | sf_1 | 3846 | AB002516.1 | Streptococcus equi subsp. zooepidemicus str. ATCC 43079 |
| Bacteria | Firmicutes | Mollicutes | Acholeplasmatales | Acholeplasmataceae | sf_1 | 4092 | AY083605.1 | Australia isolate str. BVGY |
| Bacteria | Firmicutes | Mollicutes | Mycoplasmatales | Mycoplasmataceae | sf_1 | 4102 | AF493543.1 | Mycoplasma zalophus str. 4296C |
| Bacteria | DSS1 | Unclassified | Unclassified | Unclassified | sf_1 | 4405 | AF323768.2 | benzoate-degrading consortium clone BA143 |
| Bacteria | Firmicutes | Clostridia | Clostridiales | Lachnospiraceae | sf_5 | 4155 | AB089039.1 | termite gut homogenate clone Rs-K92 bacterium |
| Bacteria | Firmicutes | Clostridia | Clostridiales | Clostridiaceae | sf_12 | 4267 | AB100485.1 | termite gut clone Rs-114 |
| Bacteria | Firmicutes | Clostridia | Clostridiales | Clostridiaceae | sf_12 | 4497 | AB089033.1 | termite gut homogenate clone Rs-N16 bacterium |
| Bacteria | OD1 | OP11-5 | Unclassified | Unclassified | sf_1 | 515 |  | |
| Bacteria | Nitrospira | Nitrospira | Nitrospirales | Nitrospiraceae | sf_1 | 864 | Y14644.1 | nitrifying sludge clone GC86 |
| Bacteria | Acidobacteria | Acidobacteria | Acidobacteriales | Acidobacteriaceae | sf_14 | 208 | AJ519665.1 | uranium mill tailings soil sample clone GuBH2-AD-9 sp. |
| Bacteria | Bacteroidetes | Bacteroidetes | Bacteroidales | Porphyromonadaceae | sf_1 | 5800 | AY253728.1 | Porphyromonas endodontalis str. ATCC 35406 |
| Bacteria | Bacteroidetes | Bacteroidetes | Bacteroidales | Prevotellaceae | sf_1 | 6109 |  | |
| Bacteria | Bacteroidetes | Bacteroidetes | Bacteroidales | Prevotellaceae | sf_1 | 5720 | AF481227.1 | Prevotella sp. str. E9_34 |
| Bacteria | Bacteroidetes | Bacteroidetes | Bacteroidales | Prevotellaceae | sf_1 | 5929 | AJ011683.1 | Prevotella albensis str. M384 |
| Bacteria | Bacteroidetes | Flavobacteria | Flavobacteriales | Flavobacteriaceae | sf_1 | 5645 | AF366270.1 | derived supragingival plaque on tooth surfaces clone DS022 sp. |
| Bacteria | Bacteroidetes | Sphingobacteria | Sphingobacteriales | Sphingobacteriaceae | sf_1 | 5459 | AJ551152.1 | Pedobacter sp. An13 |
| Bacteria | Chlorobi | Unclassified | Unclassified | Unclassified | sf_1 | 5928 | AJ441242.1 | hydrothermal vent polychaete mucous clone P. palm A 12 |
| Bacteria | Chloroflexi | Anaerolineae | Unclassified | Unclassified | sf_1 | 266 |  | |
| Bacteria | Cyanobacteria | Cyanobacteria | Thermosynechococcus | Unclassified | sf_1 | 5012 | AF448081.1 | Synechococcus sp. str. PCC 6312 |
| Bacteria | Cyanobacteria | Cyanobacteria | Chroococcales | Unclassified | sf_1 | 5219 | AF448074.1 | Synechococcus sp. str. UH7 |
| Bacteria | Unclassified | Unclassified | Unclassified | Unclassified | sf_148 | 5022 | AF454327.1 | plastid clone ML310M-37 |
| Bacteria | Spirochaetes | Spirochaetes | Spirochaetales | Spirochaetaceae | sf_1 | 6477 | AJ458945.1 | Mixotricha paradoxa is flagellate hindgut Mastotermes darwiniensis clone mp3 of |
| Bacteria | Proteobacteria | Alphaproteobacteria | Bradyrhizobiales | Beijerinck/Rhodoplan/Methylocyst | sf_3 | 7239 | AF358664.1 | Thalassospira lucentensis |
| Bacteria | Proteobacteria | Alphaproteobacteria | Unclassified | Unclassified | sf_6 | 6918 | AJ581616.1 | soil near uranium mill tailings clone KCM-C-45 |
| Bacteria | Proteobacteria | Alphaproteobacteria | Unclassified | Unclassified | sf_6 | 7647 |  | |
| Bacteria | Proteobacteria | Alphaproteobacteria | Bradyrhizobiales | Beijerinck/Rhodoplan/Methylocyst | sf_3 | 6651 | AB119196.1 | Beijerinckia indica |
| Bacteria | Proteobacteria | Alphaproteobacteria | Rhizobiales | Unclassified | sf_1 | 6756 |  | |
| Bacteria | Proteobacteria | Alphaproteobacteria | Rhizobiales | Hyphomicrobiaceae | sf_1 | 7392 |  | |
| Bacteria | Proteobacteria | Alphaproteobacteria | Bradyrhizobiales | Bradyrhizobiaceae | sf_1 | 7316 |  | |
| Bacteria | Proteobacteria | Alphaproteobacteria | Bradyrhizobiales | Bradyrhizobiaceae | sf_1 | 7333 | U87768.1 | Afipia genosp. 4 str. G3644 |
| Bacteria | Proteobacteria | Alphaproteobacteria | Bradyrhizobiales | Bradyrhizobiaceae | sf_1 | 7087 | AF530468.1 | Bradyrhizobium japonicum HA1 |
| Bacteria | Proteobacteria | Alphaproteobacteria | Bradyrhizobiales | Bradyrhizobiaceae | sf_1 | 7398 | AF208514.1 | Bradyrhizobium japonicum str. USDA 38 |
| Bacteria | Proteobacteria | Alphaproteobacteria | Bradyrhizobiales | Bradyrhizobiaceae | sf_1 | 6636 | U35000.3 | Bradyrhizobium elkanii str. USDA 76 |
| Bacteria | Proteobacteria | Alphaproteobacteria | Bradyrhizobiales | Bradyrhizobiaceae | sf_1 | 7126 | AJ534670.1 | ground water deep-well injection disposal site radioactive wastes Tomsk-7 clone S15A-MN96 proteobacterium |
| Bacteria | Proteobacteria | Alphaproteobacteria | Bradyrhizobiales | Bradyrhizobiaceae | sf_1 | 7477 | AY904744.1 | Bradyrhizobium elkanii str. SEMIA 6028 |
| Bacteria | Proteobacteria | Alphaproteobacteria | Bradyrhizobiales | Bradyrhizobiaceae | sf_1 | 7522 | AF509906.1 | Bradyrhizobium sp. str. KKI14 |
| Bacteria | Proteobacteria | Alphaproteobacteria | Bradyrhizobiales | Bradyrhizobiaceae | sf_1 | 6878 | AF530467.1 | Bradyrhizobium japonicum SD5 |
| Bacteria | Proteobacteria | Alphaproteobacteria | Rhizobiales | Phyllobacteriaceae | sf_1 | 7216 | D88524.1 | Ahrensia kielensis str. IAM12618 |
| Bacteria | Proteobacteria | Alphaproteobacteria | Rhizobiales | Rhizobiaceae | sf_1 | 6972 | AY040360.1 | Ensifer adhaerens str. LMG 20582 |
| Bacteria | Proteobacteria | Alphaproteobacteria | Rhizobiales | Rhizobiaceae | sf_1 | 6974 | AF441730.1 | India: Himalayas Kaza Spiti Valley Cold Desert isolate str. Kaza-35 Kaza-35 |
| Bacteria | Proteobacteria | Alphaproteobacteria | Rhizobiales | Rhizobiaceae | sf_1 | 7457 |  | |
| Bacteria | Proteobacteria | Alphaproteobacteria | Rhizobiales | Rhizobiaceae | sf_1 | 6964 | AE008265.2 | Agrobacterium tumefaciens str. C58 Cereon |
| Bacteria | Proteobacteria | Alphaproteobacteria | Sphingomonadales | Sphingomonadaceae | sf_1 | 7215 | AF445712.1 | travertine hot spring clone SM2B06 |
| Bacteria | Proteobacteria | Alphaproteobacteria | Unclassified | Unclassified | sf_6 | 6987 |  | |
| Bacteria | Proteobacteria | Betaproteobacteria | Neisseriales | Neisseriaceae | sf_1 | 7701 | L06174.1 | Vitreoscilla stercoraria |
| Bacteria | Proteobacteria | Betaproteobacteria | Methylophilales | Methylophilaceae | sf_1 | 7892 | AB193725.1 | Methylophilus leisingeri str. DSM 6813 |
| Bacteria | Proteobacteria | Unclassified | Unclassified | Unclassified | sf_17 | 7819 |  | |
| Bacteria | Proteobacteria | Betaproteobacteria | Nitrosomonadales | Nitrosomonadaceae | sf_1 | 7931 | L35509.1 | Nitrosospira multiformis |
| Bacteria | Proteobacteria | Betaproteobacteria | Rhodocyclales | Rhodocyclaceae | sf_1 | 8127 | AJ505852.1 | Zoogloea resiniphila str. PIV-3A2y |
| Bacteria | Proteobacteria | Betaproteobacteria | Rhodocyclales | Rhodocyclaceae | sf_1 | 8131 |  | |
| Bacteria | WS5 | Unclassified | Unclassified | Unclassified | sf_2 | 8119 | AF419661.1 | Guaymas Basin hydrothermal sediment clone a2b013 |
| Bacteria | Proteobacteria | Betaproteobacteria | Burkholderiales | Comamonadaceae | sf_1 | 8139 | AY899912.1 | Delftia tsuruhatensis str. AD9 |
| Bacteria | Proteobacteria | Betaproteobacteria | Burkholderiales | Comamonadaceae | sf_1 | 7884 | AJ421915.2 | Germany:Elbe River clone Elb37 |
| Bacteria | Proteobacteria | Betaproteobacteria | Burkholderiales | Burkholderiaceae | sf_1 | 7959 | AF476088.1 | Maconellicoccus australiensis symbiont |
| Bacteria | Proteobacteria | Betaproteobacteria | Burkholderiales | Burkholderiaceae | sf_1 | 8011 | U96941.1 | Burkholderia graminis str. AUS35 |
| Bacteria | Proteobacteria | Betaproteobacteria | Burkholderiales | Burkholderiaceae | sf_1 | 7870 | U91839.1 | Burkholderia pseudomallei str. 1026b |
| Bacteria | Proteobacteria | Betaproteobacteria | Burkholderiales | Burkholderiaceae | sf_1 | 7837 | AF311971.1 | Burkholderia cepacia LS2.4 |
| Bacteria | Proteobacteria | Betaproteobacteria | Burkholderiales | Burkholderiaceae | sf_1 | 8044 | AJ544072.1 | Burkholderia anthina str. BHS1 |
| Bacteria | Proteobacteria | Betaproteobacteria | Burkholderiales | Oxalobacteraceae | sf_1 | 8158 | AB006750.1 | 2-HNA producing isolate MC13289 |
| Bacteria | Proteobacteria | Gammaproteobacteria | Chromatiales | Chromatiaceae | sf_1 | 9571 | M96395.1 | Nitrosococcus oceanus |
| Bacteria | Proteobacteria | Gammaproteobacteria | Chromatiales | Chromatiaceae | sf_1 | 8527 |  | |
| Bacteria | Proteobacteria | Gammaproteobacteria | Cardiobacteriales | Cardiobacteriaceae | sf_1 | 9453 | AF506987.1 | Cardiobacterium valvarum str. MDA3079 |
| Bacteria | Proteobacteria | Gammaproteobacteria | Xanthomonadales | Xanthomonadaceae | sf_3 | 8392 | AY218573.1 | penguin droppings sediments clone KD2-14 |
| Bacteria | Proteobacteria | Gammaproteobacteria | Xanthomonadales | Xanthomonadaceae | sf_3 | 9569 |  | |
| Bacteria | Proteobacteria | Gammaproteobacteria | Xanthomonadales | Xanthomonadaceae | sf_3 | 8680 | AB008508.1 | ultramicrobacterium str. DY01 |
| Bacteria | Proteobacteria | Gammaproteobacteria | Legionellales | Legionellaceae | sf_1 | 8836 | CR628336.1 | Legionella pneumophila str. Paris |
| Bacteria | Proteobacteria | Gammaproteobacteria | Methylococcales | Methylococcaceae | sf_1 | 8821 | AF152597.1 | Methylobacter psychrophilus str. Z-0021 |
| Bacteria | Proteobacteria | Gammaproteobacteria | Pseudomonadales | Moraxellaceae | sf_3 | 8878 | A27627.1 | Moraxella catarrhalis |
| Bacteria | Proteobacteria | Gammaproteobacteria | Unclassified | Unclassified | sf_3 | 9044 | AF420370.1 | hydrothermal sediment clone AF420370 |
| Bacteria | Proteobacteria | Gammaproteobacteria | Alteromonadales | Alteromonadaceae | sf_1 | 8695 | AF468296.1 | Arctic pack ice; northern Fram Strait; 80 31.1 N; 01 deg 59.7 min E clone ARKIA-34 |
| Bacteria | Proteobacteria | Gammaproteobacteria | Alteromonadales | Alteromonadaceae | sf_1 | 9067 | AF006669.1 | Shewanella algae str. ACM 4733 |
| Bacteria | Proteobacteria | Gammaproteobacteria | Aeromonadales | Aeromonadaceae | sf_1 | 8621 | AF427150.1 | Aeromonas sp. PAR2A |
| Bacteria | Proteobacteria | Gammaproteobacteria | Pasteurellales | Pasteurellaceae | sf_1 | 9587 | AF268958.1 | Actinobacillus porcinus str. H1498 |
| Bacteria | Proteobacteria | Gammaproteobacteria | Enterobacteriales | Enterobacteriaceae | sf_6 | 8783 | AF075271.2 | Alterococcus agarolyticus str. ADT3; CCRC17102 |
| Bacteria | Proteobacteria | Gammaproteobacteria | Enterobacteriales | Enterobacteriaceae | sf_1 | 8886 | AF170176.1 | Salmonella typhimurium LT2 str. SGSC1412 |
| Bacteria | Proteobacteria | Gammaproteobacteria | Enterobacteriales | Enterobacteriaceae | sf_1 | 8603 | AF476106.1 | Melanococcus albizziae symbiont |
| Bacteria | Proteobacteria | Gammaproteobacteria | Enterobacteriales | Enterobacteriaceae | sf_1 | 9420 |  | |
| Bacteria | Proteobacteria | Gammaproteobacteria | Enterobacteriales | Enterobacteriaceae | sf_1 | 8934 | AF373188.1 | Pectobacterium subsp. carotovorum str. E155 subsp. |
| Bacteria | Proteobacteria | Gammaproteobacteria | Enterobacteriales | Enterobacteriaceae | sf_1 | 8528 | Y17665.1 | Enterobacter cloacae Nr. 3 |
| Bacteria | Proteobacteria | Gammaproteobacteria | Enterobacteriales | Enterobacteriaceae | sf_1 | 8530 | AJ489826.1 | Enterobacteriaceae CF01Ent-1 |
| Bacteria | Proteobacteria | Deltaproteobacteria | Desulfovibrionales | Desulfohalobiaceae | sf_1 | 10069 |  | |
| Bacteria | Proteobacteria | Deltaproteobacteria | Bdellovibrionales | Bdellovibrionaceae | sf_1 | 10010 | AJ518802.1 | uranium mining waste pile clone JG37-AG-139 proteobacterium |
| Bacteria | Proteobacteria | Deltaproteobacteria | Unclassified | Unclassified | sf_9 | 9993 |  | |
| Bacteria | Proteobacteria | Deltaproteobacteria | Desulfobacterales | Desulfobacteraceae | sf_5 | 10239 |  | |
| Bacteria | Proteobacteria | Epsilonproteobacteria | Campylobacterales | Helicobacteraceae | sf_3 | 10614 | AJ431216.1 | strain isolate str. BHI80-49 |
| Bacteria | Proteobacteria | Epsilonproteobacteria | Campylobacterales | Campylobacteraceae | sf_3 | 10523 | AF449239.1 | Riftia pachyptila's tube clone R103-B70 |
| Bacteria | Proteobacteria | Epsilonproteobacteria | Campylobacterales | Helicobacteraceae | sf_3 | 10507 | AB089111.2 | termite gut homogenate clone Rs-M59 proteobacterium |
| Bacteria | Actinobacteria | Actinobacteria | Actinomycetales | Microbacteriaceae | sf_1 | 1921 | AJ292036.1 | Firmicutes isolate str. d8 |
| Bacteria | Actinobacteria | Actinobacteria | Actinomycetales | Unclassified | sf_3 | 1583 | AB089079.1 | termite gut homogenate clone Rs-F20 bacterium |
| Bacteria | Actinobacteria | Actinobacteria | Bifidobacteriales | Bifidobacteriaceae | sf_1 | 1987 | AF287758.1 | human subgingival plaque clone CX010 |
| Bacteria | Actinobacteria | Actinobacteria | Actinomycetales | Mycobacteriaceae | sf_1 | 1180 | AJ308603.1 | Mycobacterium palustre str. E846 |
| Bacteria | Aquificae | Aquificae | Aquificales | Unclassified | sf_1 | 2364 | AY263403.1 | Thermovibrio ammoniificans str. HB-1 |
| Bacteria | Firmicutes | Clostridia | Clostridiales | Peptococc/Acidaminococc | sf_11 | 392 | AF481210.1 | oral endodontic infection clone MCE7_134 |
| Bacteria | Firmicutes | Clostridia | Clostridiales | Peptococc/Acidaminococc | sf_11 | 771 | X82500.1 | Dialister pneumosintes str. ATCC 33048 |
| Bacteria | Firmicutes | Desulfotomaculum | Unclassified | Unclassified | sf_1 | 894 | AF351222.1 | coal tar waste-contaminated groundwater clone 36-22 G+C |
| Bacteria | Firmicutes | Clostridia | Clostridiales | Peptostreptococcaceae | sf_5 | 2988 | AB100461.1 | termite gut clone Rs-050 |
| Bacteria | Firmicutes | Clostridia | Clostridiales | Peptostreptococcaceae | sf_5 | 3200 | Z36273.1 | infirmum str. W1471 |
| Bacteria | Firmicutes | Clostridia | Clostridiales | Peptostreptococcaceae | sf_5 | 140 | AY134899.1 | oral periodontitis clone EX153 |
| Bacteria | Firmicutes | Clostridia | Clostridiales | Peptostreptococcaceae | sf_5 | 436 | AF550609.1 | Peptostreptococcaceae bacterium 19gly3 |
| Bacteria | Firmicutes | Bacilli | Bacillales | Paenibacillaceae | sf_1 | 3595 | AY257871.1 | Paenibacillus sp. str. MB 2039 |
| Bacteria | Firmicutes | Bacilli | Bacillales | Bacillaceae | sf_1 | 3730 | AF541965.1 | Bacillus baekryungensis str. SW-93 |
| Bacteria | Firmicutes | Bacilli | Bacillales | Sporolactobacillaceae | sf_1 | 3879 | X92161.1 | Lake Elmenteita isolate WE4 |
| Bacteria | Firmicutes | Bacilli | Bacillales | Staphylococcaceae | sf_1 | 3865 | AY119687.1 | Macrococcus lamae str. CCM 4815 |
| Bacteria | Firmicutes | Bacilli | Lactobacillales | Lactobacillaceae | sf_1 | 3600 | AJ242969.1 | Lactobacillus crispatus str. NCTC 4 |
| Bacteria | Firmicutes | Bacilli | Lactobacillales | Lactobacillaceae | sf_1 | 3821 | M23928.1 | Lactobacillus casei |
| Bacteria | Firmicutes | Bacilli | Lactobacillales | Lactobacillaceae | sf_1 | 3842 | Y19168.1 | Lactobacillus perolens str. L534 |
| Bacteria | Firmicutes | Bacilli | Lactobacillales | Streptococcaceae | sf_1 | 3699 | AE014207.1 | Streptococcus agalactiae str. 2603V/R |
| Bacteria | Firmicutes | Bacilli | Lactobacillales | Streptococcaceae | sf_1 | 3796 | U87828.1 | aortic heart valve patient with endocarditis clone v3 |
| Bacteria | Firmicutes | Mollicutes | Mycoplasmatales | Mycoplasmataceae | sf_1 | 4014 | NC_002771.1 | Mycoplasma pulmonis str. UAB CTIP |
| Bacteria | Firmicutes | Clostridia | Clostridiales | Lachnospiraceae | sf_5 | 4366 | AJ488078.1 | chlorobenzene-degrading consortium clone IB-18 |
| Bacteria | Verrucomicrobia | Verrucomicrobiae | Verrucomicrobiales | Verrucomicrobiaceae | sf_1 | 1024 | AY244959.1 | rumen clone BS5 |
| Bacteria | Unclassified | Unclassified | Unclassified | Unclassified | sf_160 | 267 |  | |
| Bacteria | Bacteroidetes | Bacteroidetes | Bacteroidales | Prevotellaceae | sf_1 | 5426 | AF385509.1 | tongue dorsa clone DO014 |
| Bacteria | Bacteroidetes | Flavobacteria | Flavobacteriales | Flavobacteriaceae | sf_1 | 5969 | U41350.1 | Capnocytophaga ochracea str. ATCC 27872 T |
| Bacteria | Bacteroidetes | Unclassified | Unclassified | Unclassified | sf_4 | 5637 |  | |
| Bacteria | Planctomycetes | Planctomycetacia | Planctomycetales | Planctomycetaceae | sf_3 | 4831 | AJ231190.1 | Planctomyces brasiliensis |
| Bacteria | Chlamydiae | Chlamydiae | Chlamydiales | Simkaniaceae | sf_1 | 4702 | AY223862.1 | Candidatus Rhabdochlamydia porcellionis clone RKPsHep porcellionis |
| Bacteria | Cyanobacteria | Cyanobacteria | Nostocales | Unclassified | sf_1 | 5174 | AF516731.1 | Cylindrospermopsis raciborskii str. 23B |
| Bacteria | Acidobacteria | Acidobacteria-4 | Unclassified | Unclassified | sf_1 | 6363 | Z95711.1 | soil clone 32-11 |
| Bacteria | Proteobacteria | Alphaproteobacteria | Bradyrhizobiales | Bradyrhizobiaceae | sf_1 | 6768 | M59068.1 | Rhodopseudomonas palustris str. GH |
| Bacteria | Proteobacteria | Alphaproteobacteria | Bradyrhizobiales | Bradyrhizobiaceae | sf_1 | 6799 | D12700.1 | Rhodopseudomonas palustris str. ATCC 17001 |
| Bacteria | Proteobacteria | Alphaproteobacteria | Rhodobacterales | Rhodobacteraceae | sf_1 | 6652 | AF353235.1 | marine clone Arctic96A-1 |
| Bacteria | Proteobacteria | Alphaproteobacteria | Consistiales | Caedibacteraceae | sf_3 | 7010 | AJ428412.1 | periodontal pocket clone 10B6 |
| Bacteria | Proteobacteria | Alphaproteobacteria | Sphingomonadales | Unclassified | sf_1 | 6653 | AY785128.1 | Kaistobacter koreensis str. PB229 |
| Bacteria | Proteobacteria | Betaproteobacteria | Burkholderiales | Comamonadaceae | sf_1 | 7704 | AF289169.1 | freshwater clone PRD01b009B |
| Bacteria | Proteobacteria | Gammaproteobacteria | aquatic clone group | Unclassified | sf_1 | 8839 | AJ400349.1 | f cytometric sorted marine sample subpopulation 3 clone ZD0408 bacterium |
| Bacteria | Proteobacteria | Gammaproteobacteria | Thiotrichales | Piscirickettsiaceae | sf_3 | 9027 | AF064545.1 | Thiomicrospira crunogena str. XCL-2 |
| Bacteria | Proteobacteria | Betaproteobacteria | Hydrogenophilales | Hydrogenophilaceae | sf_2 | 8756 | X97534.1 | Halothiobacillus sp. |
| Bacteria | Proteobacteria | Gammaproteobacteria | Methylococcales | Methylococcaceae | sf_1 | 9065 | AY094499.1 | extracted chamber connected to Ocean Drilling Program site 892b clone 1-27 proteobacterium |
| Bacteria | Proteobacteria | Gammaproteobacteria | Oceanospirillales | Halomonadaceae | sf_1 | 8598 | X92417.1 | Halomonas desiderata str. FB2 |
| Bacteria | Proteobacteria | Gammaproteobacteria | Pseudomonadales | Pseudomonadaceae | sf_1 | 8487 |  | |
| Bacteria | Proteobacteria | Gammaproteobacteria | Alteromonadales | Alteromonadaceae | sf_1 | 8368 | AJ507251.1 | Pseudoalteromonas mariniglutinosa str. KMM 3635 |
| Bacteria | Proteobacteria | Gammaproteobacteria | Alteromonadales | Alteromonadaceae | sf_1 | 9364 | U85855.1 | Pseudoalteromonas prydzensis str. MB8-11 |
| Bacteria | Proteobacteria | Gammaproteobacteria | Alteromonadales | Shewanellaceae | sf_1 | 9081 | AB059264.1 | Shewanella sp. str. MTW-1 |
| Bacteria | Proteobacteria | Gammaproteobacteria | Vibrionales | Vibrionaceae | sf_1 | 8999 | AY292944.1 | Photobacterium leiognathi str. LN101 |
| Bacteria | Proteobacteria | Gammaproteobacteria | Pasteurellales | Pasteurellaceae | sf_1 | 8308 | M75039.1 | Haemophilus actinomycetemcomitans |
| Bacteria | Proteobacteria | Gammaproteobacteria | Enterobacteriales | Enterobacteriaceae | sf_1 | 8742 | AF084835.1 | USA:New York isolate str. KN4 |
| Bacteria | Proteobacteria | Gammaproteobacteria | Enterobacteriales | Enterobacteriaceae | sf_1 | 9358 | U92194.1 | Salmonella subsp. enterica serovar Waycross str. Swy1 subsp. |
| Bacteria | Proteobacteria | Gammaproteobacteria | Enterobacteriales | Enterobacteriaceae | sf_1 | 9496 |  | |
| Bacteria | Proteobacteria | Gammaproteobacteria | Enterobacteriales | Enterobacteriaceae | sf_1 | 8379 | AF289542.1 | Erwinia amylovora EA G-5 |
| Bacteria | Proteobacteria | Gammaproteobacteria | Enterobacteriales | Enterobacteriaceae | sf_1 | 9142 | AJ233410.1 | Erwinia amylovora str. DSM 30165 |
| Bacteria | Proteobacteria | Gammaproteobacteria | Enterobacteriales | Enterobacteriaceae | sf_1 | 9363 | AF025365.1 | Citrobacter freundii str. CDC 621-64 |
| Bacteria | Proteobacteria | Gammaproteobacteria | Enterobacteriales | Enterobacteriaceae | sf_1 | 8693 | AF130912.1 | Pantoea agglomerans str. A40 |
| Bacteria | Proteobacteria | Gammaproteobacteria | Enterobacteriales | Enterobacteriaceae | sf_1 | 9302 | AF373198.1 | Pantoea subsp. stewartii str. GSPB 2626 |
| Bacteria | Proteobacteria | Gammaproteobacteria | Enterobacteriales | Enterobacteriaceae | sf_1 | 8936 | AF543283.1 | Klebsiella oxytoca str. ChDC OS31 |
| Bacteria | Proteobacteria | Gammaproteobacteria | Enterobacteriales | Enterobacteriaceae | sf_1 | 9060 | AJ853891.1 | Enterobacter ludwigii str. EN-119 = DSMZ 16688 |
| Bacteria | Proteobacteria | Gammaproteobacteria | Enterobacteriales | Enterobacteriaceae | sf_1 | 9274 | AJ550468.1 | Enterobacter sp. CC1 |
| Bacteria | Proteobacteria | Gammaproteobacteria | Enterobacteriales | Enterobacteriaceae | sf_1 | 9361 | AB004747.1 | Enterobacter intermedius str. JCM1238 |
| Bacteria | Proteobacteria | Gammaproteobacteria | Enterobacteriales | Enterobacteriaceae | sf_1 | 9390 | Z96077.1 | Enterobacter nimipressuralis str. LMG 10245-T |
| Bacteria | Proteobacteria | Gammaproteobacteria | Enterobacteriales | Enterobacteriaceae | sf_1 | 8529 | AF181574.1 | Raoultella planticola 7 |
| Bacteria | Proteobacteria | Gammaproteobacteria | Enterobacteriales | Enterobacteriaceae | sf_1 | 8627 | AF476099.1 | Australicoccus grevilleae symbiont |
| Bacteria | Proteobacteria | Gammaproteobacteria | Enterobacteriales | Enterobacteriaceae | sf_1 | 8890 | X93216.1 | Raoultella planticola str. DR3 |
| Bacteria | Proteobacteria | Gammaproteobacteria | Enterobacteriales | Enterobacteriaceae | sf_1 | 8362 | AF453251.1 | Klebsiella pneumoniae str. ASR1 |
| Bacteria | Proteobacteria | Gammaproteobacteria | Enterobacteriales | Enterobacteriaceae | sf_1 | 8510 | AJ233420.1 | Klebsiella pneumoniae str. DSM 30104 |
| Bacteria | Proteobacteria | Gammaproteobacteria | Enterobacteriales | Enterobacteriaceae | sf_1 | 8892 | U93263.1 | Aranicola proteolyticus |
| Bacteria | Proteobacteria | Deltaproteobacteria | Desulfovibrionales | Desulfovibrionaceae | sf_1 | 10189 |  | |
| Bacteria | Proteobacteria | Deltaproteobacteria | Myxococcales | Polyangiaceae | sf_3 | 9671 | AF420357.1 | hydrothermal sediment clone AF420357 |
| Bacteria | Proteobacteria | Deltaproteobacteria | Unclassified | Unclassified | sf_9 | 9738 | AF354149.1 | marine methane seep clone 1513 |
| Bacteria | Proteobacteria | Deltaproteobacteria | Desulfobacterales | Desulfobacteraceae | sf_5 | 10267 | AJ535249.1 | marine sediment above hydrate ridge clone Hyd89-61 proteobacterium |
| Bacteria | Proteobacteria | Deltaproteobacteria | Desulfobacterales | Desulfobacteraceae | sf_5 | 10136 | AY197382.1 | Guaymas Basin hydrothermal vent sediments clone B01R011 |
| Bacteria | Proteobacteria | Epsilonproteobacteria | Campylobacterales | Helicobacteraceae | sf_3 | 10534 | U01329.1 | Helicobacter pylori |
| Bacteria | Proteobacteria | Epsilonproteobacteria | Campylobacterales | Helicobacteraceae | sf_3 | 10572 | AF057163.1 | Helicobacter sp |
| Bacteria | Fusobacteria | Fusobacteria | Fusobacterales | Fusobacteriaceae | sf_3 | 35 | AF385571.1 | crevicular epithelial cells clone EI013 |
| Bacteria | Actinobacteria | Actinobacteria | Actinomycetales | Unclassified | sf_3 | 1822 | AY145533.1 | dilution (10e-7) brackish section Weser estuary isolate str. GP-5 GP-5 |
| Bacteria | Actinobacteria | Actinobacteria | Actinomycetales | Unclassified | sf_3 | 1348 | AY250882.1 | lichen-dominated Antarctic cryptoendolithic community clone FBP406 |
| Bacteria | Actinobacteria | Actinobacteria | Actinomycetales | Streptomycetaceae | sf_1 | 1274 | AB006154.1 | Trichotomospora caesia str. IFO14562 |
| Bacteria | Actinobacteria | Actinobacteria | Actinomycetales | Microbacteriaceae | sf_1 | 1098 |  | |
| Bacteria | Actinobacteria | Actinobacteria | Actinomycetales | Cellulomonadaceae | sf_1 | 1923 | AJ229245.1 | Actinobacteria str. VeCb6 |
| Bacteria | Actinobacteria | Actinobacteria | Actinomycetales | Brevibacteriaceae | sf_1 | 1745 | X83813.1 | Brevibacterium iodinum str. DSM 2062T |
| Bacteria | Actinobacteria | Actinobacteria | Actinomycetales | Dermabacteraceae | sf_1 | 1504 | AJ415380.1 | Brachybacterium sacelli str. LMG 20338 |
| Bacteria | Actinobacteria | Actinobacteria | Actinomycetales | Micromonosporaceae | sf_1 | 1542 | AJ277575.1 | Actinoplanes garbadinensis str. IMSNU 20040 |
| Bacteria | Actinobacteria | Actinobacteria | Actinomycetales | Mycobacteriaceae | sf_1 | 1435 | AJ276890.1 | Mycobacterium cf. triplex 'isolate 23' 23 |
| Bacteria | Actinobacteria | Actinobacteria | Actinomycetales | Mycobacteriaceae | sf_1 | 1860 | AF251565.1 | Mycobacterium marinum |
| Bacteria | Actinobacteria | Actinobacteria | Actinomycetales | Corynebacteriaceae | sf_1 | 1192 | AJ439344.1 | Corynebacterium segmentosum str. CIP107068 (CCUG37878) |
| Bacteria | Firmicutes | Clostridia | Clostridiales | Peptococc/Acidaminococc | sf_11 | 126 | AY134907.1 | oral periodontitis clone FY011 |
| Bacteria | Firmicutes | Clostridia | Clostridiales | Peptococc/Acidaminococc | sf_11 | 562 | AF481224.1 | oral endodontic infection clone MCE10_265 |
| Bacteria | Firmicutes | Clostridia | Clostridiales | Peptococc/Acidaminococc | sf_11 | 800 | AJ289179.1 | corneal ulcer clone E1-K16 |
| Bacteria | Firmicutes | Clostridia | Clostridiales | Unclassified | sf_17 | 2683 |  | |
| Bacteria | Firmicutes | Clostridia | Clostridiales | Clostridiaceae | sf_12 | 2736 | AY137848.1 | Alkaliphilus metalliredigenes str. QYMF |
| Bacteria | TM7 | Unclassified | Unclassified | Unclassified | sf_1 | 3025 |  | |
| Bacteria | TM7 | TM7-3 | Unclassified | Unclassified | sf_1 | 3151 | AF513102.1 | activated sludge foam clone 71 |
| Bacteria | Firmicutes | Clostridia | Clostridiales | Peptostreptococcaceae | sf_5 | 616 | AF542230.1 | Peptoniphilus lacrimalis str. CCUG 31350 |
| Bacteria | Firmicutes | Bacilli | Bacillales | Thermoactinomycetaceae | sf_1 | 3539 | AF138738.1 | Laceyella sacchari str. KCTC 9789 () |
| Bacteria | Firmicutes | Bacilli | Bacillales | Bacillaceae | sf_1 | 3509 | AY043085.1 | Bacillus benzoevorans |
| Bacteria | Firmicutes | Bacilli | Bacillales | Bacillaceae | sf_1 | 429 | X60616.1 | Bacillus firmus str. NCIMB 9366 |
| Bacteria | Firmicutes | Bacilli | Bacillales | Bacillaceae | sf_1 | 3491 | AF326361.1 | Bacillus sp. str. MB-12 |
| Bacteria | Firmicutes | Bacilli | Bacillales | Bacillaceae | sf_1 | 3627 | AF411341.1 | Bacillus licheniformis str. SK-1 |
| Bacteria | Firmicutes | Bacilli | Bacillales | Bacillaceae | sf_1 | 3687 | AY167816.1 | Filibacter limicola str. SAFN-012 |
| Bacteria | Firmicutes | Bacilli | Lactobacillales | Leuconostocaceae | sf_1 | 3573 | AF360736.1 | Leuconostoc ficulneum str. FS-1 |
| Bacteria | Firmicutes | Bacilli | Lactobacillales | Unclassified | sf_1 | 3434 | AB089063.1 | termite gut homogenate clone Rs-C68 sp. |
| Bacteria | Firmicutes | Mollicutes | Acholeplasmatales | Acholeplasmataceae | sf_1 | 4084 | X76430.1 | Vaccinium phytopathogen isolate VAC |
| Bacteria | Firmicutes | Mollicutes | Anaeroplasmatales | Erysipelotrichaceae | sf_3 | 144 | L34616.1 | Eubacterium cylindroides |
| Bacteria | Firmicutes | Clostridia | Clostridiales | Clostridiaceae | sf_12 | 4173 | AB089020.1 | termite gut homogenate clone Rs-D81 bacterium |
| Bacteria | Firmicutes | Clostridia | Clostridiales | Clostridiaceae | sf_12 | 4406 | AB088977.1 | termite gut homogenate clone Rs-J39 bacterium |
| Bacteria | Firmicutes | Clostridia | Clostridiales | Lachnospiraceae | sf_5 | 4164 | AF376226.1 | ckncm322-B3-7 clone |
| Bacteria | Firmicutes | Anaerobranca | Unclassified | Unclassified | sf_1 | 4304 | AF507889.1 | Mono Lake at depth 35m station 6 July 2000 clone ML635J-34 G+C |
| Bacteria | Aquificae | Aquificae | Aquificales | Hydrogenothermaceae | sf_1 | 737 | AF528192.1 | Sulfurihydrogenibium azorense |
| Bacteria | Acidobacteria | Acidobacteria-10 | Unclassified | Unclassified | sf_1 | 516 | AJ532721.1 | uranium mining waste pile clone JG34-KF-153 |
| Bacteria | Acidobacteria | Acidobacteria-9 | Unclassified | Unclassified | sf_1 | 704 | AJ347029.1 | sponge clone TK29 |
| Bacteria | Bacteroidetes | Bacteroidetes | Bacteroidales | Prevotellaceae | sf_1 | 5718 | AF183403.1 | Prevotella tannerae str. 29-1 |
| Bacteria | Bacteroidetes | Bacteroidetes | Bacteroidales | Prevotellaceae | sf_1 | 5331 | AF385519.1 | tongue dorsa clone DO022 |
| Bacteria | Bacteroidetes | Bacteroidetes | Bacteroidales | Prevotellaceae | sf_1 | 5706 | U43698.1 | oral cavity clone 3.3 |
| Bacteria | Bacteroidetes | Flavobacteria | Flavobacteriales | Flavobacteriaceae | sf_1 | 5490 |  | |
| Bacteria | Cyanobacteria | Cyanobacteria | Nostocales | Unclassified | sf_1 | 5028 | AF516732.1 | Cylindrospermopsis raciborskii str. 05E |
| Bacteria | Cyanobacteria | Cyanobacteria | Nostocales | Unclassified | sf_1 | 5175 | AF516729.1 | Cylindrospermopsis raciborskii str. 24C |
| Bacteria | Cyanobacteria | Unclassified | Unclassified | Unclassified | sf_5 | 5188 | AY163573.1 | Acaryochloris marina str. MBIC11017 |
| Bacteria | Proteobacteria | Alphaproteobacteria | Acetobacterales | Roseococcaceae | sf_1 | 7500 | AF407720.1 | Great Artesian Basin clone B35 |
| Bacteria | Proteobacteria | Alphaproteobacteria | Rhizobiales | Rhizobiaceae | sf_1 | 7568 | U47303.1 | Rhizobium etli str. USDA 2667 ATCC 14483 SEMIA 043 |
| Bacteria | Proteobacteria | Alphaproteobacteria | Rhodobacterales | Rhodobacteraceae | sf_1 | 6888 | AF254109.1 | hydrothermal vent strain str. TB66 |
| Bacteria | Proteobacteria | Betaproteobacteria | Neisseriales | Neisseriaceae | sf_1 | 8143 | AY005029.1 | subgingival dental plaque clone AK105 |
| Bacteria | Proteobacteria | Betaproteobacteria | Methylophilales | Methylophilaceae | sf_1 | 8050 | AJ318156.1 | Waste-gas biofilter clone BIki2 |
| Bacteria | Proteobacteria | Betaproteobacteria | Burkholderiales | Comamonadaceae | sf_1 | 7941 | AF407397.1 | MCB-contaminated groundwater-treating reactor clone RB9C10 |
| Bacteria | Proteobacteria | Betaproteobacteria | Burkholderiales | Burkholderiaceae | sf_1 | 8059 | Y17010.1 | Burkholderia caribensis str. MWAP71 |
| Bacteria | Proteobacteria | Gammaproteobacteria | Acidithiobacillales | Acidithiobacillaceae | sf_1 | 8261 | L25709.1 | Solemya reidi symbiont 2 |
| Bacteria | Proteobacteria | Gammaproteobacteria | Symbionts | Unclassified | sf_1 | 9556 | AY129087.1 | Seepiophila jonesi symbiont |
| Bacteria | Proteobacteria | Gammaproteobacteria | Thiotrichales | Piscirickettsiaceae | sf_3 | 9392 | AJ244761.1 | Methylophaga sp. str. V4.ME.29 = MM_2343 |
| Bacteria | Proteobacteria | Gammaproteobacteria | Xanthomonadales | Xanthomonadaceae | sf_3 | 9031 | AF538773.1 | municipal wastewater treatment bioreactor clone LB-P bacterium |
| Bacteria | Proteobacteria | Gammaproteobacteria | Thiotrichales | Francisellaceae | sf_1 | 8949 | AY102612.1 | Caedibacter taeniospiralis |
| Bacteria | Proteobacteria | Gammaproteobacteria | Pseudomonadales | Moraxellaceae | sf_3 | 8574 |  | |
| Bacteria | Proteobacteria | Gammaproteobacteria | Pseudomonadales | Pseudomonadaceae | sf_1 | 8754 | AB076857.1 | Pseudomonas sp. str. P400Y-1 |
| Bacteria | Proteobacteria | Gammaproteobacteria | Alteromonadales | Alteromonadaceae | sf_1 | 8484 | AF513454.1 | Alteromonadaceae isolate str. LA50 |
| Bacteria | Proteobacteria | Gammaproteobacteria | Unclassified | Unclassified | sf_3 | 8959 | AF406527.1 | bacterioplankton clone AEGEAN_133 |
| Bacteria | Proteobacteria | Gammaproteobacteria | Alteromonadales | Shewanellaceae | sf_1 | 8201 | AB094597.1 | Shewanella surugaensis str. c959 |
| Bacteria | Proteobacteria | Gammaproteobacteria | Alteromonadales | Alteromonadaceae | sf_1 | 9222 | X82132.1 | Shewanella hanedai str. CIP 103207T |
| Bacteria | Proteobacteria | Gammaproteobacteria | Shewanella | Unclassified | sf_1 | 9344 | AF005249.1 | Shewanella algae str. ATCC 51192 |
| Bacteria | Proteobacteria | Gammaproteobacteria | Unclassified | Unclassified | sf_3 | 9367 | AF387348.1 | USA: Pacific Ocean seawater Naha Vents Hawaii isolate str. PV-4 |
| Bacteria | Proteobacteria | Gammaproteobacteria | Pasteurellales | Pasteurellaceae | sf_1 | 8830 | AF247712.1 | Actinobacillus sp. str. CCUG 24862 |
| Bacteria | Proteobacteria | Gammaproteobacteria | Enterobacteriales | Enterobacteriaceae | sf_1 | 9135 | AJ487029.1 | intestine Zophobas mori clone |
| Bacteria | Proteobacteria | Gammaproteobacteria | Enterobacteriales | Enterobacteriaceae | sf_1 | 9345 | AF141891.1 | Erwinia amylovora str. BC199(=Ea528) |
| Bacteria | Proteobacteria | Gammaproteobacteria | Enterobacteriales | Enterobacteriaceae | sf_1 | 8554 | AJ627202.1 | Kluyvera ascorbata 69 |
| Bacteria | Proteobacteria | Gammaproteobacteria | Enterobacteriales | Enterobacteriaceae | sf_1 | 8885 | AB089246.1 | Morganella morganii str. AP28 |
| Bacteria | Proteobacteria | Gammaproteobacteria | Enterobacteriales | Enterobacteriaceae | sf_1 | 8282 | AF476101.1 | Antonina pretiosa symbiont |
| Bacteria | Proteobacteria | Gammaproteobacteria | Enterobacteriales | Enterobacteriaceae | sf_1 | 8739 | AJ233427.1 | Serratia entomophila str. DSM 12358 |
| Bacteria | Proteobacteria | Gammaproteobacteria | Enterobacteriales | Enterobacteriaceae | sf_1 | 8225 |  | |
| Bacteria | Proteobacteria | Gammaproteobacteria | Enterobacteriales | Enterobacteriaceae | sf_1 | 8473 | M59155.1 | Hafnia alvei |
| Bacteria | Proteobacteria | Deltaproteobacteria | Desulfovibrionales | Desulfovibrionaceae | sf_1 | 10244 |  | |
| Bacteria | Proteobacteria | Deltaproteobacteria | Myxococcales | Polyangiaceae | sf_3 | 10082 | AJ518792.1 | uranium mining waste pile clone JG37-AG-33 proteobacterium |
| Bacteria | Proteobacteria | Deltaproteobacteria | Syntrophobacterales | Syntrophaceae | sf_3 | 9935 | AF523957.1 | forested wetland clone FW117 |
| Bacteria | Proteobacteria | Deltaproteobacteria | Desulfobacterales | Desulfobacteraceae | sf_5 | 9939 | AF354163.1 | marine methane seep clone 1427 |
| Bacteria | Proteobacteria | Deltaproteobacteria | Desulfobacterales | Desulfobacteraceae | sf_5 | 10083 | AF418175.1 | Desulfobacter curvatus str. DSM 3379 |
| Bacteria | Proteobacteria | Epsilonproteobacteria | Campylobacterales | Unclassified | sf_1 | 10384 | AF367484.1 | deep-sea hydrothermal vent clone VC1.2-cl06 |
| Bacteria | Actinobacteria | Actinobacteria | Acidimicrobiales | Microthrixineae | sf_1 | 1576 | AF454307.1 | actinobacterium clone ML817J-10 |
| Bacteria | Actinobacteria | Actinobacteria | Actinomycetales | Streptomycetaceae | sf_1 | 1639 | X60514.1 | Streptomyces coelicolor str. M145 ssp. A3(2) |
| Bacteria | Actinobacteria | Actinobacteria | Actinomycetales | Streptosporangiaceae | sf_1 | 1587 | AB039960.1 | Nonomuraea subsp. roseoviolacea str. SF 2303 |
| Bacteria | Actinobacteria | Actinobacteria | Actinomycetales | Microbacteriaceae | sf_1 | 1186 | AF505514.1 | Cryocola antiquus str. VKM 103PF |
| Bacteria | Actinobacteria | Actinobacteria | Actinomycetales | Unclassified | sf_3 | 1130 | X94155.2 | Georgenia muralis str. 1A-C |
| Bacteria | Actinobacteria | Actinobacteria | Actinomycetales | Intrasporangiaceae | sf_1 | 1935 |  | |
| Bacteria | Actinobacteria | Actinobacteria | Actinomycetales | Dermabacteraceae | sf_1 | 1677 | X91030.1 | Brachybacterium conglomeratum str. NCIB 9859 |
| Bacteria | Actinobacteria | Actinobacteria | Actinomycetales | Kineosporiaceae | sf_1 | 1893 | AF095336.1 | Kineosporia aurantiaca str. NRLL B-16913 |
| Bacteria | Firmicutes | Clostridia | Clostridiales | Lachnospiraceae | sf_5 | 2849 | X89975.1 | Butyrivibrio fibrisolvens str. NCDO 2435 |
| Bacteria | Firmicutes | Clostridia | Clostridiales | Lachnospiraceae | sf_5 | 2965 | AF481221.1 | oral endodontic infection clone MCE9_173 |
| Bacteria | Natronoanaerobium | Unclassified | Unclassified | Unclassified | sf_1 | 3745 | AF507877.1 | Mono Lake at depth 35m station 6 July 2000 clone ML635J-45 |
| Bacteria | Firmicutes | Bacilli | Bacillales | Bacillaceae | sf_1 | 246 | AJ509006.1 | Bacillus sp. 6160m-C1 |
| Bacteria | Firmicutes | Bacilli | Bacillales | Bacillaceae | sf_1 | 3898 | AB034710.1 | compost clone 4-28 |
| Bacteria | Firmicutes | Bacilli | Lactobacillales | Aerococcaceae | sf_1 | 3782 |  | |
| Bacteria | Firmicutes | Bacilli | Lactobacillales | Lactobacillaceae | sf_1 | 3395 | X76328.1 | Lactobacillus reuteri str. DSM 20016 T |
| Bacteria | Firmicutes | Clostridia | Clostridiales | Clostridiaceae | sf_12 | 4299 | AB100466.1 | termite gut clone Rs-L02 |
| Bacteria | Firmicutes | Clostridia | Clostridiales | Clostridiaceae | sf_12 | 4424 | AB088982.1 | termite gut homogenate clone Rs-M05 bacterium |
| Bacteria | Firmicutes | Clostridia | Clostridiales | Clostridiaceae | sf_12 | 4559 | AY244908.1 | cow rumen clone BF30 |
| Bacteria | Firmicutes | Clostridia | Unclassified | Unclassified | sf_3 | 4280 |  | |
| Bacteria | Verrucomicrobia | Verrucomicrobiae | Verrucomicrobiales | Verrucomicrobiaceae | sf_6 | 613 | AJ441222.1 | hydrothermal vent polychaete mucous clone P. palm C 85 |
| Bacteria | Bacteroidetes | Bacteroidetes | Bacteroidales | Prevotellaceae | sf_1 | 6066 | AY207061.1 | human mouth isolate str. P4P_62 |
| Bacteria | Unclassified | Unclassified | Unclassified | Unclassified | sf_160 | 6337 |  | |
| Bacteria | Spirochaetes | Spirochaetes | Spirochaetales | Spirochaetaceae | sf_1 | 6460 | AB088907.1 | termite gut homogenate clone Rs-B69 sp. |
| Bacteria | Spirochaetes | Spirochaetes | Spirochaetales | Spirochaetaceae | sf_1 | 6623 | AB088865.1 | termite gut homogenate clone Rs-D46 sp. |
| Bacteria | Proteobacteria | Alphaproteobacteria | Rhodobacterales | Rhodobacteraceae | sf_1 | 6701 | AF245632.1 | Roseobacter clone NAC11-3 |
| Bacteria | Proteobacteria | Alphaproteobacteria | Rhodobacterales | Rhodobacteraceae | sf_1 | 6991 | X53853.1 | Rhodobacter sphaeroides str. 2.4.1 |
| Bacteria | Proteobacteria | Alphaproteobacteria | Rhodobacterales | Rhodobacteraceae | sf_1 | 7084 | AF359546.1 | Scrippsiella trochoidea NEPCC 15 |
| Bacteria | Proteobacteria | Alphaproteobacteria | Sphingomonadales | Sphingomonadaceae | sf_1 | 7528 | D16145.1 | Sphingobium yanoikuyae str. GIFU9882 |
| Bacteria | Proteobacteria | Betaproteobacteria | Nitrosomonadales | Nitrosomonadaceae | sf_1 | 7682 | AF363291.1 | Nitrosovibrio sp. str. RY6A |
| Bacteria | Proteobacteria | Betaproteobacteria | Nitrosomonadales | Nitrosomonadaceae | sf_1 | 7796 | AF080256.1 | Nitrosospira sp. str. TYM9 |
| Bacteria | Proteobacteria | Betaproteobacteria | Burkholderiales | Alcaligenaceae | sf_1 | 7764 | AF513937.1 | Alcaligenaceae clone LA1-B29N |
| Bacteria | Proteobacteria | Betaproteobacteria | Burkholderiales | Comamonadaceae | sf_1 | 7919 | AB021339.1 | strain isolate str. rM4 |
| Bacteria | Proteobacteria | Betaproteobacteria | Burkholderiales | Comamonadaceae | sf_1 | 7929 | AF068803.1 | hydrothermal vent clone VC2.1 Bac29 |
| Bacteria | Proteobacteria | Gammaproteobacteria | Chromatiales | Chromatiaceae | sf_1 | 8546 | AJ242772.1 | Thiocapsa litoralis |
| Bacteria | Proteobacteria | Gammaproteobacteria | Xanthomonadales | Xanthomonadaceae | sf_3 | 9332 | AY096032.1 | wetland ecosystem constructed to remediate mine drainage isolate str. WJ2 WJ2 |
| Bacteria | Proteobacteria | Gammaproteobacteria | GAO cluster | Unclassified | sf_1 | 9008 | AF361092.1 | activated sludge clone SBRQ157 |
| Bacteria | Proteobacteria | Gammaproteobacteria | Alteromonadales | Shewanellaceae | sf_1 | 8662 |  | |
| Bacteria | Proteobacteria | Gammaproteobacteria | Unclassified | Unclassified | sf_3 | 9473 | AF468261.1 | Arctic pack ice; northern Fram Strait; 80 31.1 N; 01 deg 59.7 min E clone ARKDMS-58 |
| Bacteria | Proteobacteria | Gammaproteobacteria | Aeromonadales | Aeromonadaceae | sf_1 | 9245 | AB105442.1 | Batch reactor filled with cyanobacterial bloom and lake water clone -8 |
| Bacteria | Proteobacteria | Gammaproteobacteria | Enterobacteriales | Enterobacteriaceae | sf_1 | 8974 | U92197.1 | Salmonella subsp. enterica serovar Agona str. Sa1 subsp. |
| Bacteria | Proteobacteria | Gammaproteobacteria | Enterobacteriales | Unclassified | sf_1 | 8430 | AF029226.1 | Salmonella bongori str. JEO 4162 |
| Bacteria | Proteobacteria | Gammaproteobacteria | Enterobacteriales | Enterobacteriaceae | sf_1 | 8467 | AB061685.1 | Serratia marcescens subsp. sakuensis str. KRED subsp. |
| Bacteria | Proteobacteria | Gammaproteobacteria | Enterobacteriales | Enterobacteriaceae | sf_1 | 8640 |  | |
| Bacteria | Proteobacteria | Deltaproteobacteria | Desulfobacterales | Desulfobulbaceae | sf_1 | 10107 | AJ535243.1 | marine sediment above hydrate ridge clone Hyd24-30 proteobacterium |
| Bacteria | Proteobacteria | Deltaproteobacteria | Desulfobacterales | Desulfobulbaceae | sf_1 | 10332 | AF099059.1 | Psychrophilic sulfate-reducing isolate str. LSv23 bacterium |
| Bacteria | Actinobacteria | Actinobacteria | Actinomycetales | Microbacteriaceae | sf_1 | 1135 | AJ459101.1 | Rhodoglobus vestalii str. LV3 |
| Bacteria | Actinobacteria | Actinobacteria | Actinomycetales | Cellulomonadaceae | sf_1 | 1450 |  | |
| Bacteria | Actinobacteria | Actinobacteria | Actinomycetales | Propionibacteriaceae | sf_1 | 2023 | AJ315953.1 | Propionibacterium propionicum str. DSM 43307T |
| Bacteria | Actinobacteria | Actinobacteria | Actinomycetales | Micromonosporaceae | sf_1 | 1395 | D14642.1 | Catenuloplanes japonicus str. IFO14176 |
| Bacteria | Actinobacteria | Actinobacteria | Actinomycetales | Micromonosporaceae | sf_1 | 1770 | X93194.1 | Dactylosporangium roseum str. DSM 43916 |
| Bacteria | Unclassified | Unclassified | Unclassified | Unclassified | sf_160 | 2488 |  | |
| Bacteria | Firmicutes | Clostridia | Clostridiales | Peptococc/Acidaminococc | sf_11 | 865 | AF287793.1 | Selenomonas sputigena str. ATCC 35185 |
| Bacteria | Firmicutes | Clostridia | Clostridiales | Peptococc/Acidaminococc | sf_11 | 71 | AY218598.1 | penguin droppings sediments clone KD2-97 |
| Bacteria | Firmicutes | Clostridia | Clostridiales | Lachnospiraceae | sf_5 | 3087 | AY178635.1 | Butyrivibrio hungatei str. Su6 |
| Bacteria | Firmicutes | Bacilli | Bacillales | Bacillaceae | sf_1 | 3370 | AB020195.1 | Bacillus sp. str. TGS437 |
| Bacteria | Firmicutes | Bacilli | Bacillales | Bacillaceae | sf_1 | 3385 | AY167806.1 | Bacillus licheniformis str. SAFN-031 |
| Bacteria | Firmicutes | Bacilli | Bacillales | Bacillaceae | sf_1 | 3818 | AF372616.1 | Bacillus licheniformis str. Mo1 |
| Bacteria | Firmicutes | Bacilli | Bacillales | Bacillaceae | sf_1 | 3626 | L14014.1 | Bacillus sphaericus |
| Bacteria | Firmicutes | Clostridia | Clostridiales | Lachnospiraceae | sf_5 | 4301 | AF287779.1 | human subgingival plaque clone F058 |
| Bacteria | Firmicutes | Clostridia | Clostridiales | Clostridiaceae | sf_12 | 4177 | AF241842.1 | Clostridium subterminale DSM 2636 |
| Bacteria | Firmicutes | Clostridia | Clostridiales | Clostridiaceae | sf_12 | 4574 | NC_003030.1 | Clostridium acetobutylicum str. ATCC 824 |
| Bacteria | Verrucomicrobia | Verrucomicrobiae | Verrucomicrobiales | Verrucomicrobiaceae | sf_6 | 203 | AY271254.1 | Akkermansia muciniphila |
| Bacteria | OP8 | Unclassified | Unclassified | Unclassified | sf_3 | 598 | AF419671.1 | Guaymas Basin hydrothermal sediment clone a2b010 |
| Bacteria | Acidobacteria | Acidobacteria-6 | Unclassified | Unclassified | sf_1 | 500 | Z95717.1 | soil clone RB24 |
| Bacteria | Bacteroidetes | Bacteroidetes | Bacteroidales | Unclassified | sf_15 | 5907 | AY244952.1 | cow rumen clone BE36 |
| Bacteria | Bacteroidetes | Bacteroidetes | Bacteroidales | Prevotellaceae | sf_1 | 5893 | AF385515.1 | tongue dorsa clone DO045 |
| Bacteria | Bacteroidetes | Flavobacteria | Flavobacteriales | Flavobacteriaceae | sf_1 | 5434 | AF543293.1 | Capnocytophaga sp. str. ChDC OS43 |
| Bacteria | Bacteroidetes | Sphingobacteria | Sphingobacteriales | Flexibacteraceae | sf_19 | 6261 | AF468338.1 | Arctic sea ice cryoconite clone ARKCRY-50 |
| Bacteria | Cyanobacteria | Cyanobacteria | Pseudanabaena | Unclassified | sf_1 | 5008 | AF448080.1 | Synechococcus sp. str. PCC 7502 |
| Bacteria | Acidobacteria | Acidobacteria | Acidobacteriales | Acidobacteriaceae | sf_14 | 6356 | AF523985.1 | forested wetland clone FW47 |
| Bacteria | Spirochaetes | Spirochaetes | Spirochaetales | Spirochaetaceae | sf_1 | 6557 | AF454308.1 | spirochete clone ML320J-13 |
| Bacteria | Spirochaetes | Spirochaetes | Spirochaetales | Spirochaetaceae | sf_1 | 6502 | AF139203.1 | Treponema denticola str. ATCC35405 |
| Bacteria | Proteobacteria | Alphaproteobacteria | Rhizobiales | Rhizobiaceae | sf_1 | 7051 | D12786.1 | Mycoplana dimorpha str. IAM 13154 |
| Bacteria | Proteobacteria | Alphaproteobacteria | Sphingomonadales | Sphingomonadaceae | sf_1 | 7548 | U87784.1 | Afipia genosp. 13 str. G8991 |
| Bacteria | Proteobacteria | Betaproteobacteria | Neisseriales | Neisseriaceae | sf_1 | 7703 |  | |
| Bacteria | Proteobacteria | Betaproteobacteria | Burkholderiales | Comamonadaceae | sf_1 | 7822 | AY166684.1 | Polaromonas naphthalenivorans str. CJ2 |
| Bacteria | Proteobacteria | Gammaproteobacteria | Chromatiales | Chromatiaceae | sf_1 | 9048 | AJ401209.1 | Allochromatium sp. AT2202 |
| Bacteria | Proteobacteria | Gammaproteobacteria | Unclassified | Unclassified | sf_3 | 8646 |  | |
| Bacteria | OP10 | Unclassified | Unclassified | Unclassified | sf_1 | 8413 | AJ306784.1 | DCP-dechlorinating consortium clone SHA-37 |
| Bacteria | Proteobacteria | Gammaproteobacteria | Enterobacteriales | Enterobacteriaceae | sf_1 | 9337 | U90758.1 | Rahnella genosp. 3 str. DSM 30078 |
| Bacteria | Proteobacteria | Deltaproteobacteria | Desulfobacterales | Desulfobacteraceae | sf_5 | 10268 |  | |
| Bacteria | Fusobacteria | Fusobacteria | Fusobacterales | Fusobacteriaceae | sf_3 | 120 | AF432138.1 | tongue dorsum scrapings clone FP036 |
| Bacteria | Fusobacteria | Fusobacteria | Fusobacterales | Fusobacteriaceae | sf_3 | 387 | AY029802.1 | Leptotrichia wadeii str. LB16 |
| Bacteria | Actinobacteria | Actinobacteria | Coriobacteriales | Coriobacteriaceae | sf_1 | 1258 | AJ534678.1 | ground water deep-well injection disposal site radioactive wastes Tomsk-7 clone S15A-MN25 |
| Bacteria | Actinobacteria | Actinobacteria | Unclassified | Unclassified | sf_1 | 1370 | AF523912.1 | forested wetland clone RCP1-37 |
| Bacteria | Actinobacteria | Actinobacteria | Actinomycetales | Microbacteriaceae | sf_1 | 1758 | AF479358.1 | glacial ice isolate str. SB12K-2-1 |
| Bacteria | Actinobacteria | Actinobacteria | Actinomycetales | Pseudonocardiaceae | sf_1 | 1824 | AJ293756.1 | Amycolatopsis sulphurea str. IMSNU 20060T |
| Bacteria | Actinobacteria | Actinobacteria | Actinomycetales | Mycobacteriaceae | sf_1 | 1239 | AJ271863.1 | Mycobacterium ratisbonense str. SD4 |
| Bacteria | Actinobacteria | Actinobacteria | Actinomycetales | Mycobacteriaceae | sf_1 | 1365 | AY457072.1 | Mycobacterium chelonae str. CIP 104535T |
| Bacteria | Actinobacteria | Actinobacteria | Actinomycetales | Nocardiaceae | sf_1 | 1917 | X80611.1 | Nocardia otitidiscaviarum str. DSM43242 |
| Bacteria | DSS1 | Unclassified | Unclassified | Unclassified | sf_2 | 38 | AJ306783.1 | DCP-dechlorinating consortium clone SHA-109 |
| Bacteria | Firmicutes | Desulfotomaculum | Unclassified | Unclassified | sf_1 | 198 | AB091323.1 | Pelotomaculum sp. str. JT |
| Bacteria | Firmicutes | Clostridia | Clostridiales | Peptostreptococcaceae | sf_5 | 3153 | U13039.1 | infirmum str. W 1471 |
| Bacteria | Firmicutes | Clostridia | Clostridiales | Clostridiaceae | sf_12 | 3019 | AY245527.1 | Tepidibacter formicigenes str. DV1184 |
| Bacteria | Firmicutes | Clostridia | Clostridiales | Clostridiaceae | sf_12 | 2786 | AY007244.1 | Clostridium glycolicum str. CIN5 |
| Bacteria | Firmicutes | Bacilli | Bacillales | Bacillaceae | sf_1 | 3861 | AJ229201.1 | anoxic bulk soil flooded rice microcosm clone BSV46 clone |
| Bacteria | Firmicutes | Clostridia | Clostridiales | Lachnospiraceae | sf_5 | 4501 | AF385563.1 | crevicular epithelial cells clone BU014 |
| Bacteria | Deinococcus-Thermus | Unclassified | Unclassified | Unclassified | sf_1 | 886 |  | |
| Bacteria | Acidobacteria | Acidobacteria-6 | Unclassified | Unclassified | sf_1 | 350 | AY221047.1 | Mammoth cave clone CCM15a |
| Bacteria | Bacteroidetes | Bacteroidetes | Bacteroidales | Porphyromonadaceae | sf_1 | 5664 | AB088937.1 | termite gut homogenate clone Rs-E83 bacterium |
| Bacteria | Bacteroidetes | Bacteroidetes | Bacteroidales | Prevotellaceae | sf_1 | 5437 | AY244919.1 | cow rumen clone BE1 |
| Bacteria | Bacteroidetes | Bacteroidetes | Bacteroidales | Prevotellaceae | sf_1 | 5741 | L16473.1 | Prevotella veroralis |
| Bacteria | Bacteroidetes | Flavobacteria | Flavobacteriales | Flavobacteriaceae | sf_1 | 5367 | AY238333.1 | patient's bronchoalveolar lavage isolate str. MDA2507 sp. |
| Bacteria | Bacteroidetes | Flavobacteria | Flavobacteriales | Flavobacteriaceae | sf_1 | 5509 | AF539756.1 | Cellulophaga sp. str. E14 |
| Bacteria | Bacteroidetes | Sphingobacteria | Sphingobacteriales | Crenotrichaceae | sf_11 | 5334 | AB117715.1 | autotrophic nitrifying biofilm clone NB-11 |
| Bacteria | Chloroflexi | Anaerolineae | Chloroflexi-1a | Unclassified | sf_1 | 159 | AJ278169.1 | anaerobic bioreactor clone SHD-235 |
| Bacteria | Chloroflexi | Anaerolineae | Chloroflexi-1a | Unclassified | sf_1 | 487 | AB109437.1 | thermophilic UASB granular sludge isolate str. IMO-1 bacterium |
| Bacteria | Planctomycetes | Planctomycetacia | Planctomycetales | Pirellulae | sf_3 | 4692 | BX294733.1 | aerobic basin clone CY0ARA026G04 |
| Bacteria | Chlamydiae | Chlamydiae | Chlamydiales | Chlamydiaceae | sf_1 | 4820 | NC_002179.2 | Chlamydophila pneumoniae str. AR39 |
| Bacteria | Cyanobacteria | Cyanobacteria | Nostocales | Unclassified | sf_1 | 5004 | AJ630458.1 | Anabaena augstumalis 'SCHMIDKE JAHNKE/4a' str. SCMIDKE JAHNKE/4a |
| Bacteria | Cyanobacteria | Cyanobacteria | Nostocales | Unclassified | sf_1 | 5047 | AF247573.1 | Anabaena circinalis str. AWQC150A |
| Bacteria | Cyanobacteria | Cyanobacteria | Nostocales | Unclassified | sf_1 | 5072 | AF268023.1 | Nodularia spumigena str. PCC73104 |
| Bacteria | Cyanobacteria | Cyanobacteria | Nostocales | Unclassified | sf_1 | 5191 | AY038036.1 | Cyanospira rippkae str. PCC 9501 |
| Bacteria | Spirochaetes | Spirochaetes | Spirochaetales | Spirochaetaceae | sf_1 | 6575 | AY133079.1 | TCE-contaminated site clone ccslm226 |
| Bacteria | Proteobacteria | Alphaproteobacteria | Rhizobiales | Phyllobacteriaceae | sf_1 | 7381 | AJ011759.1 | Aminobacter aminovorans str. DSM7048T |
| Bacteria | Proteobacteria | Alphaproteobacteria | Consistiales | Unclassified | sf_5 | 6735 | AF510192.1 | Candidatus Pelagibacter ubique str. HTCC1002 |
| Bacteria | Proteobacteria | Alphaproteobacteria | Sphingomonadales | Sphingomonadaceae | sf_1 | 7075 | AJ000920.2 | Novosphingobium sp. str. K16 |
| Bacteria | Proteobacteria | Betaproteobacteria | Unclassified | Unclassified | sf_3 | 7997 |  | |
| Bacteria | Proteobacteria | Gammaproteobacteria | Chromatiales | Ectothiorhodospiraceae | sf_1 | 9598 | AF507818.1 | Mono Lake at depth 2 m station 6 July 2000 clone ML602J-47 proteobacterium |
| Bacteria | Proteobacteria | Gammaproteobacteria | Xanthomonadales | Xanthomonadaceae | sf_3 | 9167 | AB074619.1 | pea aphid symbiont clone APe4_38 |
| Bacteria | Proteobacteria | Gammaproteobacteria | Chromatiales | Chromatiaceae | sf_1 | 9370 | AB010860.1 | isolate str. HTB019 |
| Bacteria | Proteobacteria | Gammaproteobacteria | Alteromonadales | Alteromonadaceae | sf_1 | 9486 | AF406617.1 | Marine isolate str. GK-2001 |
| Bacteria | Proteobacteria | Gammaproteobacteria | Pasteurellales | Pasteurellaceae | sf_1 | 8195 | NZ_AADP01000001.1 | Haemophilus influenzae str. R2866 |
| Bacteria | Proteobacteria | Gammaproteobacteria | Enterobacteriales | Enterobacteriaceae | sf_1 | 9157 | AF293618.1 | Secondary symbiont type-U Acyrthosiphon pisum (rrs) clone 5B type-U |
| Bacteria | Proteobacteria | Deltaproteobacteria | Myxococcales | Polyangiaceae | sf_4 | 9733 | AF382143.1 | bacterioplankton clone ZA3735c |
| Bacteria | Proteobacteria | Deltaproteobacteria | EB1021 group | Unclassified | sf_4 | 9741 | AJ518791.1 | uranium mining waste pile clone JG37-AG-30 proteobacterium |
| Bacteria | Proteobacteria | Deltaproteobacteria | Desulfobacterales | Desulfobacteraceae | sf_5 | 9818 | AY216453.1 | temperate estuarine mud clone KM62 |
| Bacteria | Proteobacteria | Epsilonproteobacteria | Campylobacterales | Campylobacteraceae | sf_3 | 10461 | AB015259.1 | deepest cold-seep area Japan Trench clone JTB360 proteobacterium |
| Bacteria | Fusobacteria | Fusobacteria | Fusobacterales | Fusobacteriaceae | sf_3 | 694 | AF385518.1 | tongue dorsa clone DR011 |
| Bacteria | Fusobacteria | Fusobacteria | Fusobacterales | Fusobacteriaceae | sf_3 | 644 | AJ307981.1 | Ilyobacter polytropus str. DSM 2926 T |
| Bacteria | Actinobacteria | Actinobacteria | Actinomycetales | Actinomycetaceae | sf_1 | 1368 | AJ243893.1 | Actinomyces urogenitalis |
| Bacteria | Actinobacteria | Actinobacteria | Actinomycetales | Pseudonocardiaceae | sf_1 | 1402 |  | |
| Bacteria | Firmicutes | Clostridia | Clostridiales | Lachnospiraceae | sf_5 | 2756 | AY804149.1 | Roseburia faecalis str. M6/1 |
| Bacteria | Firmicutes | Bacilli | Bacillales | Paenibacillaceae | sf_1 | 3630 |  | |
| Bacteria | Firmicutes | Bacilli | Bacillales | Bacillaceae | sf_1 | 3635 | AJ504797.1 | Bacillus aeolius str. 4-1 |
| Bacteria | TM7 | Unclassified | Unclassified | Unclassified | sf_1 | 3679 |  | |
| Bacteria | Firmicutes | Bacilli | Bacillales | Bacillaceae | sf_1 | 3894 | AJ514408.1 | Sporosarcina macmurdoensis str. CMS 21w |
| Bacteria | Firmicutes | Bacilli | Lactobacillales | Streptococcaceae | sf_1 | 3543 |  | |
| Bacteria | Firmicutes | Mollicutes | Acholeplasmatales | Acholeplasmataceae | sf_1 | 3955 | AF521672.1 | Weeping tea tree witches'-broom phytoplasma tree |
| Bacteria | Firmicutes | Mollicutes | Anaeroplasmatales | Erysipelotrichaceae | sf_3 | 3943 | AB030218.1 | Coprobacillus cateniformis str. JCM 10603 |
| Bacteria | Acidobacteria | Acidobacteria | Acidobacteriales | Acidobacteriaceae | sf_14 | 209 | AJ519367.1 | uranium mining waste pile clone JG37-AG-29 sp. |
| Bacteria | Bacteroidetes | Sphingobacteria | Sphingobacteriales | Sphingobacteriaceae | sf_1 | 5513 | AF385549.1 | crevicular epithelial cells clone AZ123 |
| Bacteria | Planctomycetes | Planctomycetacia | Planctomycetales | Anammoxales | sf_4 | 4695 | AF316773.1 | Crater Lake clone CL500-15 |
| Bacteria | Cyanobacteria | Cyanobacteria | Nostocales | Unclassified | sf_1 | 5199 | AB074502.1 | Anabaena variabilis str. IAM M-204 |
| Bacteria | Proteobacteria | Unclassified | Unclassified | Unclassified | sf_20 | 6763 |  | |
| Bacteria | Proteobacteria | Betaproteobacteria | Neisseriales | Neisseriaceae | sf_1 | 8092 | AF487709.1 | Neisseria dentiae str. V33 |
| Bacteria | Proteobacteria | Betaproteobacteria | MND1 clone group | Unclassified | sf_1 | 8023 | AF293006.1 | ferromanganous micronodule clone MND1 |
| Bacteria | Proteobacteria | Betaproteobacteria | Burkholderiales | Burkholderiaceae | sf_1 | 8068 | X67039.1 | Burkholderia caryophylli str. ATCC 25418 |
| Bacteria | Proteobacteria | Gammaproteobacteria | Chromatiales | Ectothiorhodospiraceae | sf_1 | 9450 | AJ318526.1 | Halorhodospira neutrophila str. SG 3304 |
| Bacteria | Proteobacteria | Gammaproteobacteria | Thiotrichales | Thiotrichaceae | sf_3 | 8477 | AF532774.1 | marine sediment clone Limfjorden L8 |
| Bacteria | Proteobacteria | Gammaproteobacteria | Chromatiales | Chromatiaceae | sf_1 | 8408 | Y12368.2 | Thiorhodovibrio winogradskyi |
| Bacteria | Proteobacteria | Gammaproteobacteria | Chromatiales | Chromatiaceae | sf_1 | 9052 |  | |
| Bacteria | Proteobacteria | Gammaproteobacteria | uranium waste clones | Unclassified | sf_1 | 8231 | AJ536870.1 | uranium waste soil clone JG30a-KF-21 |
| Bacteria | Proteobacteria | Gammaproteobacteria | Pseudomonadales | Pseudomonadaceae | sf_1 | 8853 | AB021398.1 | Pseudomonas cichorii str. ATCC 10857T |
| Bacteria | Proteobacteria | Gammaproteobacteria | Pseudomonadales | Pseudomonadaceae | sf_1 | 9243 | Z76670.1 | Pseudomonas tolaasii str. LMG 2342T () |
| Bacteria | Proteobacteria | Unclassified | Unclassified | Unclassified | sf_28 | 10091 |  | |
| Bacteria | Proteobacteria | Deltaproteobacteria | Desulfobacterales | Desulfobacteraceae | sf_5 | 9777 | AY197376.1 | Guaymas Basin hydrothermal vent sediments clone B01R004 |
| Bacteria | Proteobacteria | Epsilonproteobacteria | Campylobacterales | Unclassified | sf_1 | 10414 | AY218582.1 | penguin droppings sediments clone KD2-34 |
| Bacteria | Actinobacteria | Actinobacteria | Actinomycetales | Microbacteriaceae | sf_1 | 1779 | AB042083.1 | Microbacterium sp. str. VKM Ac-2048 |
| Bacteria | Actinobacteria | Actinobacteria | Actinomycetales | Actinomycetaceae | sf_1 | 1672 | AJ234041.1 | Actinomyces odontolyticus str. CCUG 28084 |
| Bacteria | Actinobacteria | Actinobacteria | Actinomycetales | Actinomycetaceae | sf_1 | 2049 | AJ234059.1 | Arcanobacterium haemolyticum str. CIP 103370 |
| Bacteria | Actinobacteria | Actinobacteria | Actinomycetales | Micromonosporaceae | sf_1 | 1641 | AB196714.1 | Micromonospora marina str. JSM3-1 |
| Bacteria | Actinobacteria | Actinobacteria | Actinomycetales | Mycobacteriaceae | sf_1 | 1681 | AY227356.1 | Mycobacterium sp. str. 1B(CD) |
| Bacteria | Actinobacteria | Actinobacteria | Actinomycetales | Mycobacteriaceae | sf_1 | 2034 | X79094.1 | Mycobacterium chlorophenolicum str. PCP-I |
| Bacteria | Actinobacteria | Actinobacteria | Actinomycetales | Dietziaceae | sf_1 | 1970 | AF481211.1 | Dietzia sp. str. E9_2 |
| Bacteria | Chloroflexi | Chloroflexi-4 | Unclassified | Unclassified | sf_2 | 2532 | AF529110.1 | trichloroethene-contaminated site clone FTL276 bacterium |
| Bacteria | Firmicutes | Clostridia | Clostridiales | Peptococc/Acidaminococc | sf_11 | 866 | AF186071.1 | Veillonella ratti str. ATCC17746 |
| Bacteria | Firmicutes | Clostridia | Clostridiales | Peptococc/Acidaminococc | sf_11 | 275 | AY185322.1 | clone AKS21 |
| Bacteria | Firmicutes | Clostridia | Clostridiales | Clostridiaceae | sf_12 | 252 | AB089014.1 | termite gut homogenate clone Rs-A28 bacterium |
| Bacteria | Firmicutes | Bacilli | Bacillales | Bacillaceae | sf_1 | 3848 | AB098576.1 | Bacillus sp. str. TUT1007 |
| Bacteria | Firmicutes | Bacilli | Bacillales | Halobacillaceae | sf_1 | 3769 | AY121437.1 | Gracilibacillus sp. str. YIM-kkny13 |
| Bacteria | Firmicutes | Clostridia | Clostridiales | Lachnospiraceae | sf_5 | 4167 | AB089038.1 | termite gut homogenate clone Rs-D76 bacterium |
| Bacteria | Firmicutes | Clostridia | Clostridiales | Clostridiaceae | sf_21 | 4471 | AB100483.1 | termite gut clone Rs-058 |
| Bacteria | Verrucomicrobia | Verrucomicrobiae | Verrucomicrobiales | Unclassified | sf_4 | 169 | AY114317.1 | anoxic marine sediment clone LD1-PA26 |
| Bacteria | Bacteroidetes | Bacteroidetes | Bacteroidales | Rikenellaceae | sf_5 | 5947 | AJ441218.1 | hydrothermal vent polychaete mucous clone P. palm C/A 221 |
| Bacteria | Bacteroidetes | Sphingobacteria | Sphingobacteriales | Sphingobacteriaceae | sf_1 | 6272 | M11657.1 | Sphingobacterium heparinum |
| Bacteria | Bacteroidetes | Sphingobacteria | Sphingobacteriales | Crenotrichaceae | sf_11 | 6143 | AF534433.1 | Toolik Lake main station at 3 m depth clone TLM09/TLMdgge12a |
| Bacteria | Chlorobi | Unclassified | Unclassified | Unclassified | sf_8 | 5822 | AJ428454.1 | Saltmarsh mud clone K-790 |
| Bacteria | Planctomycetes | Planctomycetacia | Planctomycetales | Planctomycetaceae | sf_3 | 4818 | AY328733.1 | drinking water system simulator clone DSSD34 |
| Bacteria | Cyanobacteria | Cyanobacteria | Plectonema | Unclassified | sf_1 | 5210 | AY250881.1 | lichen-dominated Antarctic cryptoendolithic community clone FBP403 |
| Bacteria | Spirochaetes | Spirochaetes | Spirochaetales | Spirochaetaceae | sf_1 | 6487 | AB088866.1 | termite gut homogenate clone Rs-D52 sp. |
| Bacteria | Spirochaetes | Spirochaetes | Spirochaetales | Spirochaetaceae | sf_2 | 6493 | U42284.1 | Borrelia anserina str. ES-1 |
| Bacteria | Proteobacteria | Betaproteobacteria | Burkholderiales | Alcaligenaceae | sf_1 | 7984 | AJ318125.1 | Waste-gas biofilter clone BIfciii38 |
| Bacteria | Proteobacteria | Betaproteobacteria | Burkholderiales | Comamonadaceae | sf_1 | 8138 | AB021390.1 | Pseudomonas lanceolata str. ATCC 14669T |
| Bacteria | Proteobacteria | Betaproteobacteria | MND1 clone group | Unclassified | sf_1 | 7818 | AJ534623.1 | soil sample uranium mining waste pile near town Johanngeorgenstadt clone JG36-TzT-215 proteobacterium |
| Bacteria | Proteobacteria | Gammaproteobacteria | Unclassified | Unclassified | sf_3 | 9124 | AY145601.1 | 10e-6 dilution marine samples Weser estuary clone DC8-80-1 proteobacterium |
| Bacteria | Proteobacteria | Gammaproteobacteria | Xanthomonadales | Xanthomonadaceae | sf_3 | 8983 | AF012541.1 | Iron oxidising strain ES-1 |
| Bacteria | Proteobacteria | Gammaproteobacteria | Oceanospirillales | Oceanospirillaceae | sf_1 | 9351 | AF382102.1 | bacterioplankton clone ZA2333c |
| Bacteria | Proteobacteria | Gammaproteobacteria | Pseudomonadales | Pseudomonadaceae | sf_1 | 8288 |  | |
| Bacteria | Proteobacteria | Gammaproteobacteria | Alteromonadales | Alteromonadaceae | sf_1 | 8594 | AF482686.1 | Marinobacter sp. str. SBS |
| Bacteria | Proteobacteria | Gammaproteobacteria | Enterobacteriales | Enterobacteriaceae | sf_1 | 9151 |  | |
| Bacteria | Proteobacteria | Deltaproteobacteria | Myxococcales | Polyangiaceae | sf_3 | 9912 | AJ532713.1 | uranium mining waste pile clone JG34-KF-14 proteobacterium |
| Bacteria | Fusobacteria | Fusobacteria | Fusobacterales | Fusobacteriaceae | sf_3 | 721 | AY207053.1 | human mouth clone P2PB_51 |
| Bacteria | Actinobacteria | Actinobacteria | Acidimicrobiales | Acidimicrobiaceae | sf_1 | 2014 | AY093455.1 | marine sediment clone MB-A2-100 |
| Bacteria | Actinobacteria | Actinobacteria | Actinomycetales | Mycobacteriaceae | sf_1 | 1204 | M95488.1 | Mycobacterium sp. 3 |
| Bacteria | Actinobacteria | Actinobacteria | Actinomycetales | Mycobacteriaceae | sf_1 | 1308 | AJ431371.1 | Mycobacterium pyrenivorans str. DSM 44605 |
| Bacteria | Firmicutes | Clostridia | Clostridiales | Clostridiaceae | sf_12 | 4244 | AB089011.1 | termite gut homogenate clone Rs-K46 bacterium |
| Bacteria | Firmicutes | Clostridia | Clostridiales | Clostridiaceae | sf_12 | 4265 | AB088969.1 | termite gut homogenate clone Rs-N70 bacterium |
| Bacteria | Firmicutes | Clostridia | Clostridiales | Clostridiaceae | sf_12 | 4459 | AB100469.1 | termite gut clone Rs-109 |
| Bacteria | Thermotogae | Thermotogae | Thermotogales | Thermotogaceae | sf_4 | 51 | AJ419874.1 | Thermosipho sp. str. MV1063 |
| Bacteria | Nitrospira | Nitrospira | Nitrospirales | Nitrospiraceae | sf_2 | 542 | AF524005.1 | forested wetland clone FW19 |
| Bacteria | Bacteroidetes | Bacteroidetes | Bacteroidales | Prevotellaceae | sf_1 | 6118 |  | |
| Bacteria | Bacteroidetes | Sphingobacteria | Sphingobacteriales | Flexibacteraceae | sf_19 | 5728 | AB073564.2 | Cytophaga sp. str. MBIC04693 |
| Bacteria | Planctomycetes | Planctomycetacia | Planctomycetales | Planctomycetaceae | sf_3 | 4652 | BX294789.1 | anoxic basin clone CY0ARA028C04 |
| Bacteria | Cyanobacteria | Unclassified | Unclassified | Unclassified | sf_6 | 5186 | AY222299.1 | silica sinter depositing geothermal power station discharge drain clone ST01-SN2C |
| Bacteria | Spirochaetes | Spirochaetes | Spirochaetales | Spirochaetaceae | sf_1 | 6554 | AB088889.1 | termite gut homogenate clone Rs-A43 sp. |
| Bacteria | Proteobacteria | Alphaproteobacteria | Azospirillales | Unclassified | sf_1 | 6732 | AF426002.1 | Anabaena circinalis AWQC118C isolate str. UNSW7 |
| Bacteria | Proteobacteria | Betaproteobacteria | Neisseriales | Neisseriaceae | sf_1 | 8041 | AB076000.1 | Aquaspirillum putridiconchylium str. IAM 14964 |
| Bacteria | Proteobacteria | Betaproteobacteria | MND1 clone group | Unclassified | sf_1 | 7808 | AY221081.1 | Mammoth cave clone CCU25 |
| Bacteria | Proteobacteria | Betaproteobacteria | Burkholderiales | Comamonadaceae | sf_1 | 7928 | AY218738.1 | penguin droppings sediments clone KD5-43 |
| Bacteria | Proteobacteria | Betaproteobacteria | Burkholderiales | Comamonadaceae | sf_1 | 7888 | AB021420.1 | Hydrogenophaga flava str. DSM 619T |
| Bacteria | Proteobacteria | Gammaproteobacteria | Unclassified | Unclassified | sf_3 | 8676 |  | |
| Bacteria | Proteobacteria | Unclassified | Unclassified | Unclassified | sf_8 | 8247 |  | |
| Bacteria | Proteobacteria | Gammaproteobacteria | Alteromonadales | Alteromonadaceae | sf_1 | 8196 |  | |
| Bacteria | Proteobacteria | Gammaproteobacteria | Enterobacteriales | Enterobacteriaceae | sf_1 | 8770 |  | |
| Bacteria | Proteobacteria | Deltaproteobacteria | Myxococcales | Polyangiaceae | sf_3 | 10309 | AJ233947.1 | Nannocystis exedens str. Na e571 |
| Bacteria | Proteobacteria | Epsilonproteobacteria | Campylobacterales | Helicobacteraceae | sf_3 | 10602 |  | |
| Bacteria | Proteobacteria | Epsilonproteobacteria | Campylobacterales | Unclassified | sf_1 | 10497 | AY261811.1 | UASB reactor granular sludge clone PD-UASB-2 proteobacterium |
| Bacteria | Fusobacteria | Fusobacteria | Fusobacterales | Fusobacteriaceae | sf_3 | 1034 | AJ307982.1 | Ilyobacter tartaricus str. DSM 2382 T |
| Bacteria | Actinobacteria | Actinobacteria | Coriobacteriales | Coriobacteriaceae | sf_1 | 1926 | AB011815.1 | Collinsella aerofaciens str. JCM7791 |
| Bacteria | Actinobacteria | Actinobacteria | Actinomycetales | Kineosporiaceae | sf_1 | 1087 | AB003931.1 | Kineosporia aurantiaca str. JCM3230 |
| Bacteria | Actinobacteria | Actinobacteria | Actinomycetales | Micromonosporaceae | sf_1 | 1931 | X80823.1 | Actinoplanes utahensis str. ATCC 31044 |
| Bacteria | Actinobacteria | Actinobacteria | Actinomycetales | Mycobacteriaceae | sf_1 | 1888 | AY012577.2 | Mycobacterium brisbanense str. W6743; ATCC 49938 |
| Bacteria | Firmicutes | Clostridia | Clostridiales | Peptococc/Acidaminococc | sf_11 | 2473 |  | |
| Bacteria | SR1 | Unclassified | Unclassified | Unclassified | sf_1 | 2900 |  | |
| Bacteria | Firmicutes | Clostridia | Clostridiales | Peptostreptococcaceae | sf_5 | 2710 | AJ306755.1 | DCP-dechlorinating consortium clone SHA-74 |
| Bacteria | TM7 | TM7-3 | Unclassified | Unclassified | sf_1 | 3145 | AF385520.1 | tongue dorsa clone DR034 |
| Bacteria | Firmicutes | Bacilli | Bacillales | Halobacillaceae | sf_1 | 3633 | AY152839.1 | Bacillus clausii str. GMBAE 42 |
| Bacteria | Firmicutes | Bacilli | Lactobacillales | Streptococcaceae | sf_1 | 3637 | AF227836.1 | str. 37236 |
| Bacteria | Firmicutes | Mollicutes | Acholeplasmatales | Acholeplasmataceae | sf_1 | 3961 | L33766.1 | Clover yellow edge mycoplasma-like organism |
| Bacteria | Chloroflexi | Anaerolineae | Chloroflexi-1f | Unclassified | sf_1 | 765 | AJ278167.1 | anaerobic bioreactor clone SHD-71 |
| Bacteria | Bacteroidetes | Bacteroidetes | Bacteroidales | Unclassified | sf_15 | 5578 | AJ534685.1 | ground water deep-well injection disposal site radioactive wastes Tomsk-7 clone S15A-MN91 |
| Bacteria | Bacteroidetes | Flavobacteria | Flavobacteriales | Flavobacteriaceae | sf_1 | 6140 |  | |
| Bacteria | Cyanobacteria | Unclassified | Unclassified | Unclassified | sf_5 | 5015 | AB093489.1 | Chlorogloeopsis fritschii str. PCC 6912 |
| Bacteria | Proteobacteria | Alphaproteobacteria | Bradyrhizobiales | Unclassified | sf_1 | 7264 | AF508112.1 | Bosea thiooxidans TJ1 |
| Bacteria | Proteobacteria | Alphaproteobacteria | Rhizobiales | Bradyrhizobiaceae | sf_1 | 7029 |  | |
| Bacteria | Proteobacteria | Alphaproteobacteria | Bradyrhizobiales | Bradyrhizobiaceae | sf_1 | 7390 | U87781.1 | Afipia genosp. 10 str. G8996 |
| Bacteria | Proteobacteria | Betaproteobacteria | Burkholderiales | Comamonadaceae | sf_1 | 7705 | AY218644.1 | penguin droppings sediments clone KD4-7 |
| Bacteria | Proteobacteria | Betaproteobacteria | Burkholderiales | Burkholderiaceae | sf_1 | 8097 | AB091761.1 | Burkholderia cepacia |
| Bacteria | Proteobacteria | Gammaproteobacteria | Unclassified | Unclassified | sf_3 | 8488 |  | |
| Bacteria | Proteobacteria | Gammaproteobacteria | Pseudomonadales | Pseudomonadaceae | sf_1 | 9588 | AF530072.1 | Pseudomonas citronellolis str. TERIDB18 |
| Bacteria | Proteobacteria | Gammaproteobacteria | Pasteurellales | Pasteurellaceae | sf_1 | 9477 | AY613549.1 | Haemophilus influenzae str. M11105 |
| Bacteria | Proteobacteria | Deltaproteobacteria | Unclassified | Unclassified | sf_7 | 10048 |  | |
| Bacteria | Planctomycetes | Planctomycetacia | Planctomycetales | Anammoxales | sf_4 | 9662 | AF407728.1 | Great Artesian Basin clone B83 |
| Bacteria | Proteobacteria | Deltaproteobacteria | Syntrophobacterales | Syntrophaceae | sf_3 | 10067 | AF323766.1 | benzoate-degrading consortium clone BA044 |
| Bacteria | Fusobacteria | Fusobacteria | Fusobacterales | Fusobacteriaceae | sf_3 | 558 | AJ344093.1 | Sneathia sanguinegens str. CCUG 41628T |
| Bacteria | Actinobacteria | Actinobacteria | Actinomycetales | Micromonosporaceae | sf_1 | 1689 | AB047516.1 | Actinoplanes yunnanensis str. IFO 14459T |
| Bacteria | Actinobacteria | Actinobacteria | Actinomycetales | Mycobacteriaceae | sf_1 | 1781 | AF498650.1 | Mycobacterium rhodesiae str. JS60 |
| Bacteria | Actinobacteria | Actinobacteria | Actinomycetales | Mycobacteriaceae | sf_1 | 1885 | AF480593.1 | Mycobacterium neoaurum str. ATCC 25795 |
| Bacteria | Firmicutes | Bacilli | Bacillales | Bacillaceae | sf_1 | 3845 | AF252326.1 | hot synthetic compost clone pPD15 |
| Bacteria | Firmicutes | Bacilli | Lactobacillales | Lactobacillaceae | sf_1 | 3490 | AY675248.1 | Lactobacillus suntoryeus str. LH |
| Bacteria | Firmicutes | Clostridia | Clostridiales | Clostridiaceae | sf_12 | 4560 | X68180.1 | Clostridium beijerinckii str. NCIMB9362 |
| Bacteria | Acidobacteria | Acidobacteria | Acidobacteriales | Acidobacteriaceae | sf_14 | 964 | AJ519392.1 | uranium mining waste pile clone JG37-AG-145 sp. |
| Bacteria | Bacteroidetes | Bacteroidetes | Bacteroidales | Unclassified | sf_15 | 5511 | AJ535256.1 | marine sediment above hydrate ridge clone Hyd-B2-1 bacterium |
| Bacteria | Bacteroidetes | Sphingobacteria | Sphingobacteriales | Crenotrichaceae | sf_11 | 5463 | AB078068.1 | Flexibacter sancti str. IFO 16034 |
| Bacteria | Bacteroidetes | Sphingobacteria | Sphingobacteriales | Flexibacteraceae | sf_19 | 6297 | AF502211.1 | EBPR sludge lab scale clone HP1A92 |
| Bacteria | Chloroflexi | Anaerolineae | Chloroflexi-1a | Unclassified | sf_1 | 76 | AJ306749.1 | DCP-dechlorinating consortium clone SHA-147 |
| Bacteria | Cyanobacteria | Cyanobacteria | Plectonema | Unclassified | sf_1 | 5010 | AF170757.1 | LPP-group cyanobacterium isolate str. QSSC5cya QSSC5cya |
| Bacteria | Spirochaetes | Spirochaetes | Spirochaetales | Spirochaetaceae | sf_1 | 6489 | AJ458944.1 | Mixotricha paradoxa is flagellate hindgut Mastotermes darwiniensis clone mp1 of |
| Bacteria | Spirochaetes | Spirochaetes | Spirochaetales | Leptospiraceae | sf_3 | 6470 | AY082470.1 | neutral pH mine biofilm clone 44a-B1-48 |
| Bacteria | Proteobacteria | Alphaproteobacteria | Rhodobacterales | Rhodobacteraceae | sf_1 | 7026 | AY005463.1 | Leisingera methylohalidivorans str. MB2 |
| Bacteria | Proteobacteria | Betaproteobacteria | MND1 clone group | Unclassified | sf_1 | 7750 | AJ518782.1 | uranium mining waste pile near Johanngeorgenstadt soil clone JG37-AG-35 |
| Bacteria | Proteobacteria | Gammaproteobacteria | Acidithiobacillales | Acidithiobacillaceae | sf_1 | 8320 | AF543507.1 | acid mine drainage clone BA11 |
| Bacteria | Proteobacteria | Gammaproteobacteria | Thiotrichales | Piscirickettsiaceae | sf_3 | 8664 | AJ237758.1 | Thiomicrospira sp. str. Milos-T2 |
| Bacteria | Proteobacteria | Gammaproteobacteria | Cardiobacteriales | Cardiobacteriaceae | sf_1 | 8536 | M35014.1 | Cardiobacterium hominis |
| Bacteria | Proteobacteria | Gammaproteobacteria | Ellin307/WD2124 | Unclassified | sf_1 | 9458 | AJ518786.1 | uranium mining waste pile clone JG37-AG-94 proteobacterium |
| Bacteria | Proteobacteria | Gammaproteobacteria | Enterobacteriales | Enterobacteriaceae | sf_1 | 8700 | AF489427.1 | Baumannia cicadellinicola |
| Bacteria | Proteobacteria | Deltaproteobacteria | Desulfuromonadales | Geobacteraceae | sf_1 | 9956 | AF523968.1 | forested wetland clone FW140 |
| Bacteria | Actinobacteria | Actinobacteria | Acidimicrobiales | Microthrixineae | sf_1 | 1964 | AF406548.1 | bacterioplankton clone AEGEAN_247 |
| Bacteria | Actinobacteria | Actinobacteria | Actinomycetales | Mycobacteriaceae | sf_1 | 1187 | AF498656.1 | Mycobacterium aichiense str. JS618 |
| Bacteria | Firmicutes | Clostridia | Clostridiales | Syntrophomonadaceae | sf_5 | 2483 | AF529116.1 | trichloroethene-contaminated site clone FTLM142 bacterium |
| Bacteria | Firmicutes | Clostridia | Clostridiales | Peptococc/Acidaminococc | sf_11 | 862 | AF473834.1 | Megasphaera micronuciformis str. AIP 412.00; CIP 107280 |
| Bacteria | Firmicutes | Clostridia | Clostridiales | Peptococc/Acidaminococc | sf_11 | 300 | AY214183.1 | benzene-contaminated groundwater clone ZZ12C8 |
| Bacteria | Firmicutes | Bacilli | Bacillales | Paenibacillaceae | sf_1 | 3559 | AF378696.1 | Paenibacillus turicensis str. MOL722 |
| Bacteria | Firmicutes | Bacilli | Bacillales | Bacillaceae | sf_1 | 3467 | AJ419629.1 | Bacillus luciferensis str. LMG 18422 |
| Bacteria | Bacteroidetes | Bacteroidetes | Bacteroidales | Rikenellaceae | sf_5 | 5889 | AB088945.1 | termite gut homogenate clone Rs-F73 bacterium |
| Bacteria | Planctomycetes | Planctomycetacia | Planctomycetales | Pirellulae | sf_3 | 4704 | BX294700.1 | aerobic basin clone CY0ARA025E11 |
| Bacteria | Cyanobacteria | Unclassified | Unclassified | Unclassified | sf_9 | 5038 | AF544207.1 | Rumen isolate str. YS2 |
| Bacteria | Spirochaetes | Spirochaetes | Spirochaetales | Spirochaetaceae | sf_1 | 6507 | AB085161.1 | termite gut clone NkS-Ste2 |
| Bacteria | Proteobacteria | Alphaproteobacteria | Rhizobiales | Unclassified | sf_1 | 7060 |  | |
| Bacteria | Proteobacteria | Alphaproteobacteria | Rhizobiales | Phyllobacteriaceae | sf_1 | 6857 | AY195844.1 | Mesorhizobium mediterraneum str. PECA20 |
| Bacteria | Proteobacteria | Betaproteobacteria | Unclassified | Unclassified | sf_3 | 8045 |  | |
| Bacteria | Proteobacteria | Betaproteobacteria | Burkholderiales | Burkholderiaceae | sf_1 | 7914 | AB021422.1 | Burkholderia andropogonis str. ATCC 19311T |
| Bacteria | Proteobacteria | Betaproteobacteria | Burkholderiales | Oxalobacteraceae | sf_1 | 7743 | AF361023.1 | Herbaspirillum sp. str. NAH4 |
| Bacteria | Proteobacteria | Gammaproteobacteria | Pseudomonadales | Pseudomonadaceae | sf_1 | 9175 | AF405328.1 | Pseudomonas extremorientalis str. KMM3447 |
| Bacteria | TM6 | Unclassified | Unclassified | Unclassified | sf_1 | 10203 | AY043739.1 | forest soil clone NOS7.2WL |
| Bacteria | Proteobacteria | Epsilonproteobacteria | Campylobacterales | Helicobacteraceae | sf_3 | 10425 | AB088432.1 | Sulfurimonas autotrophica str. OK5 |
| Bacteria | Proteobacteria | Epsilonproteobacteria | Campylobacterales | Campylobacteraceae | sf_3 | 10463 | AF482990.1 | Campylobacter subsp. fetus |
| Bacteria | Actinobacteria | Actinobacteria | Actinomycetales | Gordoniaceae | sf_1 | 1116 | X79286.1 | Gordona terrae |
| Bacteria | Actinobacteria | Actinobacteria | Actinomycetales | Gordoniaceae | sf_1 | 1567 |  | |
| Bacteria | Actinobacteria | Actinobacteria | Actinomycetales | Gordoniaceae | sf_1 | 1545 |  | |
| Bacteria | Actinobacteria | Actinobacteria | Actinomycetales | Corynebacteriaceae | sf_1 | 1089 | AF537600.1 | Corynebacterium mucifaciens National Microbiology Laboratory Special identifier 01-0118 |
| Bacteria | Firmicutes | Bacilli | Lactobacillales | Aerococcaceae | sf_1 | 3866 | AF349724.1 | Turicibacter sanguinis |
| Bacteria | Firmicutes | Bacilli | Lactobacillales | Aerococcaceae | sf_1 | 3840 | X87150.1 | Trichococcus pasteurii str. KoTa2 |
| Bacteria | Firmicutes | Mollicutes | Acholeplasmatales | Acholeplasmataceae | sf_1 | 4064 | AJ542542.1 | Malus domestica isolate str. AT1/93 |
| Bacteria | Synergistes | Unclassified | Unclassified | Unclassified | sf_3 | 740 | AF371930.1 | swine intestine clone p-4292-4Wa3 |
| Bacteria | Bacteroidetes | Flavobacteria | Flavobacteriales | Flavobacteriaceae | sf_1 | 5521 | AF493689.1 | Flavobacteriaceae str. SW269 |
| Bacteria | Bacteroidetes | Bacteroidetes | Bacteroidales | Unclassified | sf_15 | 5784 | AY838556.1 | fruiting body Pleurotus eryngii clone PE01 |
| Bacteria | Bacteroidetes | Bacteroidetes | Bacteroidales | Bacteroidaceae | sf_6 | 5792 | AF513095.1 | activated sludge foam clone 47 |
| Bacteria | Chlorobi | Unclassified | Unclassified | Unclassified | sf_8 | 549 | AY118151.1 | benzene-degrading nitrate-reducing consortium clone Cart-N2 bacterium |
| Bacteria | Unclassified | Unclassified | Unclassified | Unclassified | sf_34 | 4668 |  | |
| Bacteria | Spirochaetes | Spirochaetes | Spirochaetales | Spirochaetaceae | sf_1 | 6565 | AB085166.1 | termite gut clone NkS-Oxy25 |
| Bacteria | Spirochaetes | Spirochaetes | Spirochaetales | Spirochaetaceae | sf_1 | 6579 | AB084968.1 | termite gut clone NkS83 |
| Bacteria | Proteobacteria | Alphaproteobacteria | Bradyrhizobiales | Bradyrhizobiaceae | sf_1 | 6887 | AB067729.1 | Bradyrhizobium str. YB2 |
| Bacteria | Proteobacteria | Betaproteobacteria | Rhodocyclales | Rhodocyclaceae | sf_1 | 8052 | AY032610.1 | Dechloromonas aromatica str. RCB |
| Bacteria | Proteobacteria | Betaproteobacteria | Burkholderiales | Burkholderiaceae | sf_1 | 7969 | U37342.1 | Burkholderia sp. |
| Bacteria | Proteobacteria | Gammaproteobacteria | Thiotrichales | Piscirickettsiaceae | sf_3 | 9557 | AF449225.1 | Riftia pachyptila's tube clone R76-B23 |
| Bacteria | Proteobacteria | Unclassified | Unclassified | Unclassified | sf_8 | 9558 |  | |
| Bacteria | Proteobacteria | Gammaproteobacteria | Pseudomonadales | Pseudomonadaceae | sf_1 | 9050 | AF139998.1 | Pseudomonas sp. str. ML-124 |
| Bacteria | Proteobacteria | Gammaproteobacteria | Alteromonadales | Alteromonadaceae | sf_1 | 9411 | AY170366.1 | Shewanella waksmanii str. KMM 3823 |
| Bacteria | Proteobacteria | Deltaproteobacteria | Desulfobacterales | Desulfobacteraceae | sf_5 | 9666 | AJ535229.1 | marine sediment above hydrate ridge clone Hyd89-13 proteobacterium |
| Bacteria | Actinobacteria | Actinobacteria | Actinomycetales | Micromonosporaceae | sf_1 | 1760 | AJ277583.1 | Actinoplanes roseosporangius str. IMSNU 22133 |
| Bacteria | Actinobacteria | Actinobacteria | Actinomycetales | Gordoniaceae | sf_1 | 1654 | AF416719.1 | Gordonia polyisoprenivorans str. Y2K |
| Bacteria | Firmicutes | Bacilli | Lactobacillales | Carnobacteriaceae | sf_1 | 3792 | AF451271.1 | Carnobacterium sp. str. D35 |
| Bacteria | Bacteroidetes | Bacteroidetes | Bacteroidales | Unclassified | sf_15 | 5353 | AF529128.1 | trichloroethene-contaminated site clone FTLpost3 bacterium |
| Bacteria | Bacteroidetes | Flavobacteria | Flavobacteriales | Flavobacteriaceae | sf_1 | 6246 | AF385569.1 | crevicular epithelial cells clone BU084 |
| Bacteria | Bacteroidetes | Sphingobacteria | Sphingobacteriales | Flexibacteraceae | sf_19 | 5307 | AB078082.1 | Microscilla sericea str. IFO 16561 |
| Bacteria | Spirochaetes | Spirochaetes | Spirochaetales | Spirochaetaceae | sf_1 | 6529 | AB062806.1 | termite gut homogenate clone BCf10-21 |
| Bacteria | Proteobacteria | Betaproteobacteria | Burkholderiales | Comamonadaceae | sf_1 | 7986 | AF468446.1 | Arctic sea ice ARK10281 |
| Bacteria | Proteobacteria | Betaproteobacteria | Burkholderiales | Comamonadaceae | sf_1 | 7987 | AB076844.1 | Acidovorax sp. str. OS-6 |
| Bacteria | Proteobacteria | Gammaproteobacteria | Acidithiobacillales | Acidithiobacillaceae | sf_1 | 8552 | AJ278723.1 | Acidithiobacillus ferrooxidans str. D2 |
| Bacteria | Proteobacteria | Gammaproteobacteria | Unclassified | Unclassified | sf_3 | 9105 |  | |
| Bacteria | Proteobacteria | Gammaproteobacteria | Enterobacteriales | Enterobacteriaceae | sf_1 | 9290 | AF465797.1 | Baumannia cicadellinicola |
| Bacteria | Proteobacteria | Deltaproteobacteria | dechlorinating clone group | Unclassified | sf_1 | 9959 | AF523965.1 | forested wetland clone FW110 |
| Bacteria | Proteobacteria | Epsilonproteobacteria | Campylobacterales | Helicobacteraceae | sf_23 | 10443 | U00679.1 | Helicobacter pylori str. 85D08 |
| Bacteria | Proteobacteria | Epsilonproteobacteria | Campylobacterales | Helicobacteraceae | sf_3 | 10576 | AF142585.1 | Helicobacter sp. 'liver 3' str. liver 3 |
| Bacteria | Actinobacteria | Actinobacteria | Actinomycetales | Acidothermaceae | sf_1 | 1399 | AJ532700.1 | uranium mill tailings clone Gitt-KF-183 |
| Bacteria | Actinobacteria | Actinobacteria | Actinomycetales | Gordoniaceae | sf_1 | 1191 | X80635.1 | Gordonia amarae str. DSM43392 |
| Bacteria | Actinobacteria | Actinobacteria | Actinomycetales | Nocardiaceae | sf_1 | 1432 | AF430055.1 | Nocardia veterana str. DSM 44445 |
| Bacteria | Actinobacteria | Actinobacteria | Actinomycetales | Nocardiaceae | sf_1 | 1787 | AF430046.1 | Nocardia pseudovaccinii str. DSM 43406 |
| Bacteria | OP9/JS1 | JS1 | Unclassified | Unclassified | sf_1 | 2491 | AY093469.1 | deep marine sediment clone MB-B2-103 |
| Bacteria | Firmicutes | Clostridia | Clostridiales | Lachnospiraceae | sf_5 | 2893 | AY169411.1 | Ruminococcus obeum |
| Bacteria | Firmicutes | Bacilli | Bacillales | Halobacillaceae | sf_1 | 3552 | AB087828.1 | Paraliobacillus ryukyuensis |
| Bacteria | Firmicutes | Bacilli | Lactobacillales | Lactobacillaceae | sf_1 | 3366 | AY255802.1 | Lactobacillus saerimneri str. GDA154 LMG 22087 DSM 16049 (T); CCUG 48462 (T) |
| Bacteria | Firmicutes | Bacilli | Lactobacillales | Aerococcaceae | sf_1 | 3736 | Y14650.1 | Desemzia incerta str. DSM 20581 |
| Bacteria | Firmicutes | Clostridia | Clostridiales | Clostridiaceae | sf_12 | 4296 | AB081585.1 | Clostridiaceae str. A4d |
| Bacteria | Firmicutes | Clostridia | Clostridiales | Clostridiaceae | sf_12 | 4378 |  | |
| Bacteria | Proteobacteria | Deltaproteobacteria | Desulfobacterales | Unclassified | sf_3 | 468 | AJ241004.1 | marine sediment clone Sva0515 |
| Bacteria | Bacteroidetes | Flavobacteria | Flavobacteriales | Flavobacteriaceae | sf_1 | 5726 | AY008691.1 | Bergeyella sp. oral AK152 clone |
| Bacteria | Bacteroidetes | Bacteroidetes | Bacteroidales | Unclassified | sf_15 | 5355 | AJ306736.1 | DCP-dechlorinating consortium clone SHA-5 |
| Bacteria | Spirochaetes | Spirochaetes | Spirochaetales | Spirochaetaceae | sf_1 | 6532 | AB084963.1 | termite gut clone NkS39 |
| Bacteria | Proteobacteria | Deltaproteobacteria | EB1021 group | Unclassified | sf_4 | 8169 | AF523886.1 | forested wetland clone RCP2-54 |
| Bacteria | Proteobacteria | Gammaproteobacteria | Pseudomonadales | Pseudomonadaceae | sf_1 | 9343 | AF448515.1 | Cellvibrio subsp. mixtus str. ACM 2601 |
| Bacteria | Proteobacteria | Gammaproteobacteria | Enterobacteriales | Enterobacteriaceae | sf_1 | 9309 | AJ417833.1 | Buchnera sp |
| Bacteria | Proteobacteria | Deltaproteobacteria | Desulfobacterales | Desulfobacteraceae | sf_5 | 10046 | AJ237603.1 | Desulfobacterium cetonicum str. DSM 7267 oil recovery water |
| Bacteria | Proteobacteria | Epsilonproteobacteria | Campylobacterales | Helicobacteraceae | sf_3 | 10518 | X67854.1 | Helicobacter pylori str. ATCC 49396T |
| Bacteria | Gemmatimonadetes | Unclassified | Unclassified | Unclassified | sf_5 | 2047 | AF234148.1 | soil clone #0319-7G21 |
| Bacteria | Actinobacteria | Actinobacteria | Actinomycetales | Streptomycetaceae | sf_1 | 1375 | AF423240.1 | soil clone 228-1 |
| Bacteria | Actinobacteria | Actinobacteria | Actinomycetales | Micrococcaceae | sf_1 | 1212 | AB071952.1 | Rothia aeria str. GTC 867 |
| Bacteria | Actinobacteria | Actinobacteria | Actinomycetales | Micromonosporaceae | sf_1 | 1615 | AB036999.1 | Actinoplanes derwentensis str. IFO 14935T |
| Bacteria | Actinobacteria | Actinobacteria | Actinomycetales | Pseudonocardiaceae | sf_1 | 1849 |  | |
| Bacteria | Actinobacteria | Actinobacteria | Actinomycetales | Nocardiaceae | sf_1 | 1146 | AF430068.1 | Nocardia otitidiscaviarum str. S639 |
| Bacteria | Firmicutes | Clostridia | Clostridiales | Peptococc/Acidaminococc | sf_11 | 59 | AF371694.1 | swine intestine clone p-1941-s962-3 |
| Bacteria | Firmicutes | Clostridia | Clostridiales | Peptococc/Acidaminococc | sf_11 | 1036 | AF407698.1 | Great Artesian Basin clone G07 |
| Bacteria | Firmicutes | Mollicutes | Acholeplasmatales | Acholeplasmataceae | sf_1 | 3975 | AF302841.1 | Black raspberry witches'-broom phytoplasma str. BRWB witches'-broom |
| Bacteria | Firmicutes | Catabacter | Unclassified | Unclassified | sf_1 | 4261 | AB089006.1 | termite gut homogenate clone Rs-G04 bacterium |
| Bacteria | Firmicutes | Catabacter | Unclassified | Unclassified | sf_4 | 4325 | AB088963.1 | termite gut homogenate clone Rs-K21 bacterium |
| Bacteria | Firmicutes | Clostridia | Clostridiales | Clostridiaceae | sf_12 | 4275 | M59087.1 | Clostridium botulinum str. type G |
| Bacteria | Nitrospira | Nitrospira | Nitrospirales | Nitrospiraceae | sf_2 | 697 | AF524003.1 | forested wetland clone FW118 |
| Bacteria | Bacteroidetes | Bacteroidetes | Bacteroidales | Prevotellaceae | sf_1 | 6045 | AY689230.1 | Prevotella nigrescens str. ChDC B270 |
| Bacteria | Bacteroidetes | Flavobacteria | Flavobacteriales | Flavobacteriaceae | sf_1 | 5695 | AJ535225.1 | marine sediment above hydrate ridge clone Hyd24-41 bacterium |
| Bacteria | Bacteroidetes | Flavobacteria | Flavobacteriales | Flavobacteriaceae | sf_1 | 5436 | AF468406.1 | Arctic sea ice ARK10004 |
| Bacteria | Proteobacteria | Alphaproteobacteria | Sphingomonadales | Sphingomonadaceae | sf_1 | 7100 | D16147.1 | Novosphingobium capsulatum str. GIFU11526 |
| Bacteria | Proteobacteria | Gammaproteobacteria | Unclassified | Unclassified | sf_3 | 9498 |  | |
| Bacteria | Proteobacteria | Gammaproteobacteria | Pseudomonadales | Pseudomonadaceae | sf_1 | 9172 | AB041885.1 | Pseudomonas psychrophila str. E-3 |
| Bacteria | Proteobacteria | Gammaproteobacteria | Alteromonadales | Pseudoalteromonadaceae | sf_1 | 9339 | AF284230.1 | Pseudoalteromonas sp. str. 05 |
| Bacteria | Proteobacteria | Deltaproteobacteria | Desulfobacterales | Desulfobulbaceae | sf_1 | 10062 | AJ535241.1 | marine sediment above hydrate ridge clone Hyd24-11 proteobacterium |
| Bacteria | Actinobacteria | Actinobacteria | Actinomycetales | Actinomycetaceae | sf_1 | 1649 | AJ540309.1 | Actinomyces graevenitzii str. CCUG 27294 |
| Bacteria | Firmicutes | Clostridia | Clostridiales | Clostridiaceae | sf_12 | 3219 | AF407695.1 | Great Artesian Basin clone R82 |
| Bacteria | Firmicutes | Bacilli | Bacillales | Paenibacillaceae | sf_1 | 3415 | AF480937.1 | Paenibacillus nematophilus str. NEM1b |
| Bacteria | Firmicutes | Bacilli | Lactobacillales | Aerococcaceae | sf_1 | 3631 | D50541.1 | Abiotrophia defectiva str. GIFU12707 (ATCC49176) |
| Bacteria | Firmicutes | Bacilli | Lactobacillales | Lactobacillaceae | sf_1 | 3829 | AJ417500.1 | Lactobacillus paralimentarius str. DSM 13238 |
| Bacteria | Firmicutes | Bacilli | Lactobacillales | Aerococcaceae | sf_1 | 3326 | AF255736.1 | Nostocoida limicola I str. Ben206 |
| Bacteria | Firmicutes | Clostridia | Clostridiales | Lachnospiraceae | sf_5 | 4623 | AJ408957.1 | human colonic clone HuCA1 |
| Bacteria | Bacteroidetes | Flavobacteria | Flavobacteriales | Flavobacteriaceae | sf_1 | 5906 | X67609.1 | Capnocytophaga sputigena str. ATCC 33612 |
| Bacteria | Bacteroidetes | Sphingobacteria | Sphingobacteriales | Crenotrichaceae | sf_11 | 5619 | AY211072.1 | anaerobic VC-degrading enrichment clone VC10 bacterium |
| Bacteria | Bacteroidetes | Sphingobacteria | Sphingobacteriales | Flexibacteraceae | sf_19 | 5542 | AB073595.2 | Cytophaga sp. I-1787 |
| Bacteria | Planctomycetes | Planctomycetacia | Planctomycetales | Planctomycetaceae | sf_3 | 4855 | BX294726.1 | aerobic basin clone CY0ARA026D10 |
| Bacteria | Acidobacteria | Acidobacteria | Acidobacteriales | Acidobacteriaceae | sf_14 | 6412 | AF047646.1 | acid mine drainage clone TRB82 |
| Bacteria | Proteobacteria | Betaproteobacteria | Unclassified | Unclassified | sf_3 | 8036 | AJ519624.1 | Uranium mill tailings soil sample clone Sh765B-TzT-132 proteobacterium |
| Bacteria | Proteobacteria | Gammaproteobacteria | Chromatiales | Chromatiaceae | sf_1 | 9054 |  | |
| Bacteria | Proteobacteria | Gammaproteobacteria | Pseudomonadales | Pseudomonadaceae | sf_1 | 8725 | AJ419674.1 | Pseudomonas sp. str. 2N1-1 |
| Bacteria | Proteobacteria | Gammaproteobacteria | Pseudomonadales | Pseudomonadaceae | sf_1 | 9493 | AF430125.1 | Pseudomonas sp. str. dcm7B |
| Bacteria | Proteobacteria | Gammaproteobacteria | Pasteurellales | Pasteurellaceae | sf_1 | 9349 | AF227861.1 | Pasteurella sp. str. 91985 |
| Bacteria | NC10 | Unclassified | Unclassified | Unclassified | sf_1 | 2516 | AF529103.1 | TCE-contaminated site clone FTL22 |
| Bacteria | Firmicutes | Clostridia | Clostridiales | Lachnospiraceae | sf_5 | 3109 | X87151.1 | Catonella morbi str. ATCC 51271 |
| Bacteria | Firmicutes | Clostridia | Clostridiales | Peptostreptococcaceae | sf_5 | 2796 | AF287764.1 | human subgingival plaque clone BB142 |
| Bacteria | Firmicutes | Bacilli | Bacillales | Bacillaceae | sf_1 | 3650 |  | |
| Bacteria | Firmicutes | Bacilli | Lactobacillales | Aerococcaceae | sf_1 | 3522 | M58797.1 | Aerococcus viridans |
| Bacteria | Bacteroidetes | Flavobacteria | Flavobacteriales | Flavobacteriaceae | sf_1 | 5955 | AJ244701.1 | Flavobacterium sp. str. V4.MS.29 = MM_2747 |
| Bacteria | Spirochaetes | Spirochaetes | Spirochaetales | Spirochaetaceae | sf_1 | 6583 | AB088875.1 | termite gut homogenate clone Rs-J42 sp. |
| Bacteria | Proteobacteria | Betaproteobacteria | Rhodocyclales | Rhodocyclaceae | sf_1 | 7817 | AY133064.1 | TCE-contaminated site clone ccs265 |
| Bacteria | Proteobacteria | Betaproteobacteria | Burkholderiales | Comamonadaceae | sf_1 | 8012 | AJ420325.1 | Acidovorax konjaci str. DSM 7481 |
| Bacteria | Proteobacteria | Gammaproteobacteria | Thiotrichales | Thiotrichaceae | sf_3 | 8321 | AF532769.1 | Wadden Sea sediment clone Dangast A9 |
| Bacteria | Proteobacteria | Gammaproteobacteria | Pseudomonadales | Moraxellaceae | sf_3 | 9641 | X81662.1 | Acinetobacter haemolyticus |
| Bacteria | Proteobacteria | Gammaproteobacteria | Pseudomonadales | Pseudomonadaceae | sf_1 | 9300 | AY150183.1 | Lyrodus pedicellatus symbiont |
| Bacteria | Proteobacteria | Gammaproteobacteria | Pseudomonadales | Pseudomonadaceae | sf_1 | 8687 | AF094738.1 | Pseudomonas putida str. ATCC 17472 |
| Bacteria | Proteobacteria | Gammaproteobacteria | Alteromonadales | Alteromonadaceae | sf_1 | 9149 | AF081498.1 | Pseudoalteromonas piscicida str. Cura-d |
| Bacteria | Proteobacteria | Deltaproteobacteria | Desulfovibrionales | Desulfovibrionaceae | sf_1 | 10212 |  | |
| Bacteria | Actinobacteria | Actinobacteria | Actinomycetales | Streptosporangiaceae | sf_1 | 1190 | U48977.1 | Nonomuraea polychroma str. IFO 14345 |
| Bacteria | Actinobacteria | Actinobacteria | Actinomycetales | Microbacteriaceae | sf_1 | 1241 | AJ717353.1 | Microbacterium kitamiense CV88 |
| Bacteria | Actinobacteria | Actinobacteria | Actinomycetales | Microbacteriaceae | sf_1 | 1404 | AJ507468.1 | freshwater isolate str. MWH-Ta3 |
| Bacteria | Firmicutes | Clostridia | Clostridiales | Lachnospiraceae | sf_5 | 3001 | AJ408972.1 | human colonic clone HuCA20 |
| Bacteria | Firmicutes | Clostridia | Clostridiales | Peptostreptococcaceae | sf_5 | 1037 | AY169425.1 | Finegoldia magna |
| Bacteria | Chloroflexi | Anaerolineae | Chloroflexi-1a | Unclassified | sf_1 | 927 | AJ441227.1 | hydrothermal vent polychaete mucous clone P. palm C 37 |
| Bacteria | Deinococcus-Thermus | Unclassified | Unclassified | Unclassified | sf_1 | 178 | AY028380.1 | Thermus sp. str. C4 |
| Bacteria | Bacteroidetes | Sphingobacteria | Sphingobacteriales | Flexibacteraceae | sf_19 | 5602 |  | |
| Bacteria | Unclassified | Unclassified | Unclassified | Unclassified | sf_160 | 7767 |  | |
| Bacteria | Proteobacteria | Gammaproteobacteria | Pseudomonadales | Pseudomonadaceae | sf_1 | 8338 | AF267911.1 | Pseudomonas synxantha str. DSM 13080 G |
| Bacteria | Proteobacteria | Gammaproteobacteria | Pasteurellales | Pasteurellaceae | sf_1 | 9030 | AY216870.1 | Volucribacter amazonae str. B96/5 |
| Bacteria | Proteobacteria | Gammaproteobacteria | Enterobacteriales | Enterobacteriaceae | sf_1 | 9293 | AJ245598.1 | USA clone 14/7 |
| Bacteria | Proteobacteria | Deltaproteobacteria | Syntrophobacterales | Syntrophobacteraceae | sf_1 | 10221 | AF482439.1 | granular sludge clone R3p4 |
| Bacteria | Proteobacteria | Deltaproteobacteria | Desulfobacterales | Desulfobulbaceae | sf_1 | 10187 | AF507840.1 | Mono Lake at depth 23 m station 6 July 2000 clone ML623J-57 proteobacterium |
| Bacteria | Actinobacteria | Actinobacteria | Rubrobacterales | Rubrobacteraceae | sf_1 | 1901 | AY039806.1 | earthworm burrow isolate B33D1 |
| Bacteria | Actinobacteria | Actinobacteria | Actinomycetales | Unclassified | sf_3 | 1296 | AB089080.1 | termite gut homogenate clone Rs-M66 bacterium |
| Bacteria | Actinobacteria | Actinobacteria | Actinomycetales | Unclassified | sf_3 | 1900 |  | |
| Bacteria | Actinobacteria | Actinobacteria | Actinomycetales | Mycobacteriaceae | sf_1 | 1726 | X52925.1 | Mycobacterium terrae str. ATCC 15755 |
| Bacteria | Firmicutes | Mollicutes | Acholeplasmatales | Acholeplasmataceae | sf_1 | 4045 | AY081817.1 | Chinaberry yellows phytoplasma |
| Bacteria | Firmicutes | Mollicutes | Entomoplasmatales | Entomoplasmataceae | sf_2 | 4112 | AY157871.1 | Entomoplasma somnilux str. ATCC 49194 |
| Bacteria | SPAM | Unclassified | Unclassified | Unclassified | sf_1 | 738 | AJ532725.1 | uranium mining waste clone JG34-KF-252 |
| Bacteria | Acidobacteria | Acidobacteria-4 | Ellin6075/11-25 | Unclassified | sf_1 | 790 | Z95709.1 | soil clone 11-25 |
| Bacteria | Acidobacteria | Acidobacteria-6 | Unclassified | Unclassified | sf_1 | 897 | AY221071.1 | Mammoth cave clone CCM8b |
| Bacteria | Bacteroidetes | Bacteroidetes | Bacteroidales | Prevotellaceae | sf_1 | 5403 | AJ006457.1 | Prevotella bryantii str. B14 (DSM 11371 species ) |
| Bacteria | Bacteroidetes | Sphingobacteria | Sphingobacteriales | Sphingobacteriaceae | sf_1 | 5492 | AF492000.1 | Sphingobacterium sp. str. HC-6155 |
| Bacteria | Bacteroidetes | Sphingobacteria | Sphingobacteriales | Flexibacteraceae | sf_19 | 5994 | AJ549285.1 | Hymenobacter sp. str. NS/50 |
| Bacteria | Bacteroidetes | Sphingobacteria | Sphingobacteriales | Flexibacteraceae | sf_19 | 5366 | AB078061.1 | Flexibacter roseolus str. IFO 16030 |
| Bacteria | Cyanobacteria | Unclassified | Unclassified | Unclassified | sf_9 | 5164 | AB089123.1 | termite gut homogenate clone Rs-H34 |
| Bacteria | Cyanobacteria | Cyanobacteria | Spirulina | Unclassified | sf_1 | 5034 | AF329394.1 | Spirulina subsalsa str. FACHB351 |
| Bacteria | Spirochaetes | Spirochaetes | Spirochaetales | Spirochaetaceae | sf_1 | 6494 | AB088890.1 | termite gut homogenate clone Rs-C47 sp. |
| Bacteria | Proteobacteria | Alphaproteobacteria | Unclassified | Unclassified | sf_6 | 7340 | AJ536878.1 | uranium mining waste pile soil sample clone JG30-KF-AS50 |
| Bacteria | Proteobacteria | Gammaproteobacteria | Thiotrichales | Thiotrichaceae | sf_3 | 8559 | L40998.1 | Thioploca ingrica |
| Bacteria | Proteobacteria | Gammaproteobacteria | Methylococcales | Methylococcaceae | sf_1 | 9438 | AJ535221.1 | marine sediment above hydrate ridge clone Hyd24-01 proteobacterium |
| Bacteria | Proteobacteria | Gammaproteobacteria | Pseudomonadales | Pseudomonadaceae | sf_1 | 8513 | AF064458.1 | Pseudomonas monteilii str. CIP 104883 |
| Bacteria | Proteobacteria | Gammaproteobacteria | Pseudomonadales | Pseudomonadaceae | sf_1 | 9221 | AJ492830.1 | Pseudomonas fulgida str. DSM 14938 = LMG 2146 P 515/12 |
| Bacteria | Proteobacteria | Epsilonproteobacteria | Campylobacterales | Helicobacteraceae | sf_3 | 10417 | AY216452.1 | temperate estuarine mud clone KM61 |
| Bacteria | Actinobacteria | Actinobacteria | Actinomycetales | Kineosporiaceae | sf_1 | 1581 | AJ293746.1 | Cryptosporangium aurantiacum str. IMSNU 22120 |
| Bacteria | Actinobacteria | Actinobacteria | Actinomycetales | Mycobacteriaceae | sf_1 | 1650 | X58890.1 | Mycobacterium tuberculosis str. NCTC 7416 H37Rv |
| Bacteria | Firmicutes | Clostridia | Clostridiales | Lachnospiraceae | sf_5 | 3236 | AF287770.1 | Firmicutes sp. oral strain str. FTB41 |
| Bacteria | Firmicutes | Clostridia | Clostridiales | Clostridiaceae | sf_12 | 3108 | Z69940.1 | Clostridium paradoxum str. DSM 7308T |
| Bacteria | Firmicutes | Bacilli | Lactobacillales | Leuconostocaceae | sf_1 | 3497 | AY035892.1 | Weissella koreensis S-5673 |
| Bacteria | Firmicutes | Bacilli | Lactobacillales | Lactobacillaceae | sf_1 | 3547 | AJ250074.1 | Lactobacillus frumenti str. TMW 1.666 |
| Bacteria | Firmicutes | Bacilli | Lactobacillales | Streptococcaceae | sf_1 | 3560 | AJ307888.1 | Streptococcus gallinaceus str. CCUG 42692 |
| Bacteria | Firmicutes | Clostridia | Clostridiales | Clostridiaceae | sf_12 | 4418 | AB088966.2 | termite gut homogenate clone Rs-H18 bacterium |
| Bacteria | Firmicutes | Clostridia | Clostridiales | Clostridiaceae | sf_12 | 4627 | AB089030.1 | termite gut homogenate clone Rs-A13 bacterium |
| Bacteria | Firmicutes | Clostridia | Clostridiales | Clostridiaceae | sf_12 | 4637 | AB100473.1 | termite gut clone Rs-088 |
| Bacteria | Firmicutes | Clostridia | Clostridiales | Clostridiaceae | sf_12 | 4607 | X68188.1 | Clostridium novyi str. NCTC538 |
| Bacteria | Firmicutes | Clostridia | Clostridiales | Clostridiaceae | sf_12 | 4427 | L08062.1 | Clostridium tyrobutyricum str. NIZO 51 |
| Bacteria | Acidobacteria | Acidobacteria-4 | Ellin6075/11-25 | Unclassified | sf_1 | 435 | AY211077.1 | anaerobic VC-degrading enrichment clone VC47 bacterium |
| Bacteria | Acidobacteria | Acidobacteria | Acidobacteriales | Acidobacteriaceae | sf_14 | 6378 | D26171.1 | Acidobacterium capsulatum |
| Bacteria | Proteobacteria | Alphaproteobacteria | Rickettsiales | Rickettsiaceae | sf_1 | 6809 | U11021.1 | Rickettsia rickettsii str. Sawtooth |
| Bacteria | Proteobacteria | Betaproteobacteria | Burkholderiales | Comamonadaceae | sf_1 | 8152 | AJ543434.1 | nephridia Octolasion lacteum clone Ol2-2 |
| Bacteria | Proteobacteria | Betaproteobacteria | Burkholderiales | Burkholderiaceae | sf_1 | 7957 | AF476089.1 | Maconellicoccus hirsutus symbiont |
| Bacteria | Proteobacteria | Betaproteobacteria | Burkholderiales | Oxalobacteraceae | sf_1 | 8058 | Y10146.1 | Herbaspirillum seropedicae str. DSM 6445 ATCC 35892 |
| Bacteria | Proteobacteria | Gammaproteobacteria | Symbionts | Unclassified | sf_1 | 8403 | AF432146.1 | Selenate-reducing isolate str. KE4OH1 |
| Bacteria | Proteobacteria | Gammaproteobacteria | Thiotrichales | Francisellaceae | sf_1 | 9554 | AF206675.1 | Tilapia parasite TPT-541 |
| Bacteria | Proteobacteria | Deltaproteobacteria | EB1021 group | Unclassified | sf_4 | 9884 | AF523885.1 | forested wetland clone RCP2-62 |
| Bacteria | Proteobacteria | Deltaproteobacteria | EB1021 group | Unclassified | sf_4 | 10024 | AF420338.1 | hydrothermal sediment clone AF420338 |
| Bacteria | Actinobacteria | Actinobacteria | Rubrobacterales | Rubrobacteraceae | sf_1 | 1551 | AF498683.1 | soil isolate Ellin301 |
| Bacteria | Actinobacteria | Actinobacteria | Rubrobacterales | Rubrobacteraceae | sf_1 | 1739 |  | |
| Bacteria | Actinobacteria | Actinobacteria | Bifidobacteriales | Bifidobacteriaceae | sf_1 | 1835 | AY513712.1 | Bifidobacterium breve str. KB 92 |
| Bacteria | Actinobacteria | Actinobacteria | Actinomycetales | Mycobacteriaceae | sf_1 | 1262 | AJ310467.1 | Mycobacterium holsaticum str. 1406 |
| Bacteria | Firmicutes | Clostridia | Clostridiales | Lachnospiraceae | sf_5 | 2801 | AY169412.1 | Butyrivibrio fibrisolvens |
| Bacteria | Firmicutes | Clostridia | Clostridiales | Peptostreptococcaceae | sf_5 | 2993 | AF538856.1 | oral clone P2PB_46 P3 |
| Bacteria | TM7 | TM7-3 | Unclassified | Unclassified | sf_1 | 2697 | AJ576410.1 | midgut homogenate Pachnoda ephippiata larva clone PeM47 |
| Bacteria | Firmicutes | Bacilli | Lactobacillales | Lactobacillaceae | sf_1 | 3767 | AJ575744.1 | Lactobacillus suebicus str. CECT 5917T |
| Bacteria | Firmicutes | Mollicutes | Unclassified | Unclassified | sf_6 | 149 | M23730.1 | Streptococcus pleomorphus |
| Bacteria | Bacteroidetes | Flavobacteria | Flavobacteriales | Flavobacteriaceae | sf_1 | 6248 | AY005073.1 | Capnocytophaga sp. oral strain str. S3 |
| Bacteria | Unclassified | Unclassified | Unclassified | Unclassified | sf_160 | 6360 |  | |
| Bacteria | Proteobacteria | Alphaproteobacteria | Ellin329/Riz1046 | Unclassified | sf_1 | 6945 | AB081581.3 | Rhizobiales str. A48 |
| Bacteria | Proteobacteria | Gammaproteobacteria | Pseudomonadales | Pseudomonadaceae | sf_1 | 9002 | AJ316016.1 | Paederus fuscipes endosymbiont |
| Bacteria | Proteobacteria | Gammaproteobacteria | Pseudomonadales | Pseudomonadaceae | sf_1 | 9238 |  | |
| Bacteria | Proteobacteria | Gammaproteobacteria | Pseudomonadales | Pseudomonadaceae | sf_1 | 9028 | AF468452.1 | Pseudomonas koreensis str. Ps 9-14 |
| Bacteria | Proteobacteria | Gammaproteobacteria | Pseudomonadales | Pseudomonadaceae | sf_1 | 8601 | AB021401.1 | Pseudomonas marginalis str. ATCC 10844T |
| Bacteria | Proteobacteria | Gammaproteobacteria | Alteromonadales | Alteromonadaceae | sf_1 | 9501 | AB086226.1 | sea water isolate str. BP-PH |
| Bacteria | Proteobacteria | Gammaproteobacteria | Alteromonadales | Alteromonadaceae | sf_1 | 9369 | X82144.1 | Pseudoalteromonas luteoviolacea str. NCIMB 1893T |
| Bacteria | Proteobacteria | Gammaproteobacteria | Enterobacteriales | Enterobacteriaceae | sf_1 | 8182 | X07652.1 | Proteus vulgaris str. IFAM 1731 |
| Bacteria | Actinobacteria | Actinobacteria | Actinomycetales | Thermomonosporaceae | sf_1 | 1741 | AJ293710.1 | Actinomadura pelletieri str. IMSNU 22169T |
| Bacteria | Actinobacteria | Actinobacteria | Actinomycetales | Dermatophilaceae | sf_1 | 1852 | AB096085.1 | Tonsillophilus suis str. HT1-19 |
| Bacteria | Actinobacteria | Actinobacteria | Bifidobacteriales | Bifidobacteriaceae | sf_1 | 1109 | AY148470.1 | Bifidobacterium thermacidophilum porcinum subsp. suis str. P3-14 subsp. |
| Bacteria | Actinobacteria | Actinobacteria | Actinomycetales | Micromonosporaceae | sf_1 | 1488 | AJ277574.1 | Actinoplanes utahensis str. IMSNU 20044T |
| Bacteria | Actinobacteria | Actinobacteria | Actinomycetales | Gordoniaceae | sf_1 | 1184 | AB065369.1 | Gordonia alkanivorans |
| Bacteria | Firmicutes | Catabacter | Unclassified | Unclassified | sf_4 | 2716 | AB089010.1 | termite gut homogenate clone Rs-F76 bacterium |
| Bacteria | Firmicutes | Bacilli | Bacillales | Halobacillaceae | sf_1 | 3756 | AY121439.1 | Salibacillus sp. str. YIM-kkny16 |
| Bacteria | Firmicutes | Bacilli | Lactobacillales | Lactobacillaceae | sf_1 | 3521 | AJ271383.1 | Pediococcus inopinatus str. DSM 20285 |
| Bacteria | Chlorobi | Chlorobia | Chlorobiales | Chlorobiaceae | sf_1 | 995 | AB054671.1 | Chlorobium limicola str. M1 |
| Bacteria | Spirochaetes | Spirochaetes | Spirochaetales | Spirochaetaceae | sf_1 | 6523 | AB088912.1 | termite gut homogenate clone Rs-J64 sp. |
| Bacteria | Proteobacteria | Alphaproteobacteria | Unclassified | Unclassified | sf_6 | 7312 |  | |
| Bacteria | Proteobacteria | Betaproteobacteria | Burkholderiales | Comamonadaceae | sf_1 | 8112 | AF336312.1 | Comamonas testosteroni str. SMCC B329 |
| Bacteria | Proteobacteria | Gammaproteobacteria | Pseudomonadales | Pseudomonadaceae | sf_1 | 8433 | AB001441.1 | Pseudomonas syringae pv. broussonetiae str. KOZ 8101 pv. |
| Bacteria | Proteobacteria | Gammaproteobacteria | Pseudomonadales | Pseudomonadaceae | sf_1 | 8561 | AF332541.1 | Pseudomonas sp. B65 |
| Bacteria | Proteobacteria | Gammaproteobacteria | Pseudomonadales | Pseudomonadaceae | sf_1 | 8708 |  | |
| Bacteria | Proteobacteria | Gammaproteobacteria | Alteromonadales | Alteromonadaceae | sf_1 | 9384 | AJ132226.1 | Moritella viscosa str. NVI 88/478T |
| Bacteria | Unclassified | Unclassified | Unclassified | Unclassified | sf_160 | 1914 |  | |
| Bacteria | Actinobacteria | Actinobacteria | Actinomycetales | Unclassified | sf_3 | 1486 | AY093463.1 | deep marine sediment clone MB-A2-108 |
| Bacteria | Actinobacteria | Actinobacteria | Bifidobacteriales | Bifidobacteriaceae | sf_1 | 1875 |  | |
| Bacteria | Actinobacteria | Actinobacteria | Actinomycetales | Micromonosporaceae | sf_1 | 1159 | AJ277568.1 | Actinoplanes durhamensis str. IMSNU 22124T |
| Bacteria | Chloroflexi | Unclassified | Unclassified | Unclassified | sf_12 | 2523 | AJ347055.1 | sponge clone TK10 |
| Bacteria | Firmicutes | Clostridia | Clostridiales | Lachnospiraceae | sf_5 | 2709 | AY916314.1 | human stool clone B065 |
| Bacteria | Firmicutes | Clostridia | Clostridiales | Peptostreptococcaceae | sf_5 | 2722 | AB088987.2 | termite gut homogenate clone Rs-E61 bacterium |
| Bacteria | Firmicutes | Clostridia | Clostridiales | Lachnospiraceae | sf_5 | 4434 | AB089036.1 | termite gut homogenate clone Rs-K11 bacterium |
| Bacteria | Firmicutes | Clostridia | Clostridiales | Clostridiaceae | sf_12 | 4575 | X68451.1 | Clostridium subterminale str. NCIMB 10746 |
| Bacteria | Cyanobacteria | Unclassified | Unclassified | Unclassified | sf_5 | 5027 |  | |
| Bacteria | Acidobacteria | Solibacteres | Unclassified | Unclassified | sf_1 | 6426 | AF407714.1 | Great Artesian Basin clone B11 |
| Bacteria | Spirochaetes | Spirochaetes | Spirochaetales | Spirochaetaceae | sf_1 | 6476 | AB084965.1 | termite gut clone NkS50 |
| Bacteria | Proteobacteria | Alphaproteobacteria | Bradyrhizobiales | Bradyrhizobiaceae | sf_1 | 7353 | AY216437.1 | temperate estuarine mud clone HC65 |
| Bacteria | Proteobacteria | Alphaproteobacteria | Consistiales | Unclassified | sf_4 | 6810 | AY033325.1 | marine bacterioplankton clone MB13F01 |
| Bacteria | Proteobacteria | Alphaproteobacteria | Rhodobacterales | Unclassified | sf_5 | 7471 | AJ347025.1 | sponge clone TK03 |
| Bacteria | Proteobacteria | Alphaproteobacteria | Rickettsiales | Rickettsiaceae | sf_1 | 7556 | U11014.1 | Rickettsia bellii str. strains 369-C and G2D42 |
| Bacteria | Proteobacteria | Alphaproteobacteria | Sphingomonadales | Sphingomonadaceae | sf_1 | 6650 | AY563441.1 | Sphingomonas phyllosphaerae str. FA1 |
| Bacteria | Proteobacteria | Betaproteobacteria | Burkholderiales | Oxalobacteraceae | sf_1 | 7866 | AB021375.1 | Paucimonas lemoignei str. ATCC 17989T |
| Bacteria | Proteobacteria | Gammaproteobacteria | GAO cluster | Unclassified | sf_1 | 9059 | AF361096.1 | activated sludge clone SBRH10 |
| Bacteria | Proteobacteria | Gammaproteobacteria | Oceanospirillales | Unclassified | sf_3 | 8230 |  | |
| Bacteria | Proteobacteria | Gammaproteobacteria | Pseudomonadales | Pseudomonadaceae | sf_1 | 8344 | AF425998.1 | Anabaena circinalis AWQC118C isolate str. UNSW3 |
| Bacteria | Proteobacteria | Gammaproteobacteria | Pseudomonadales | Pseudomonadaceae | sf_1 | 8850 | D88521.1 | Agrobacterium agile str. IAM12615 |
| Bacteria | Proteobacteria | Gammaproteobacteria | Pseudomonadales | Pseudomonadaceae | sf_1 | 9049 | AJ296559.1 | uranium mining mill tailing clone GR-Sh2-34 GR-Sh2-34 |
| Bacteria | Actinobacteria | Actinobacteria | Actinomycetales | Micromonosporaceae | sf_1 | 1633 | AJ277585.1 | Actinoplanes tuftoflagellus str. IMSNU 22135 |
| Bacteria | Actinobacteria | Actinobacteria | Actinomycetales | Micromonosporaceae | sf_1 | 1972 | AB037011.1 | Actinoplanes regularis str. IFO 12514T |
| Bacteria | Actinobacteria | Actinobacteria | Actinomycetales | Micromonosporaceae | sf_1 | 1120 | AJ277586.1 | Actinoplanes violaceus str. IMSNU 22136 |
| Bacteria | Firmicutes | Clostridia | Clostridiales | Peptostreptococcaceae | sf_5 | 2738 | AB037875.1 | Mogibacterium neglectum str. ATCC 700924 (=P9a-h) |
| Bacteria | Firmicutes | Bacilli | Bacillales | Bacillaceae | sf_1 | 3419 | AY228462.1 | Bacillus algicola str. KMM 3737 |
| Bacteria | Gemmatimonadetes | Unclassified | Unclassified | Unclassified | sf_5 | 442 | AF507712.1 | forest soil clone S0134 |
| Bacteria | Bacteroidetes | Flavobacteria | Flavobacteriales | Flavobacteriaceae | sf_1 | 5730 |  | |
| Bacteria | Planctomycetes | Planctomycetacia | Planctomycetales | Pirellulae | sf_3 | 4803 | X86388.1 | Pirellula sp. str. ACM 3181 |
| Bacteria | Cyanobacteria | Cyanobacteria | Chloroplasts | Chloroplasts | sf_5 | 5130 | AF445714.1 | travertine hot spring clone SM2B11 |
| Bacteria | Proteobacteria | Betaproteobacteria | Burkholderiales | Burkholderiaceae | sf_1 | 7782 | AY040365.1 | Burkholderia hospita str. LMG 20598T |
| Bacteria | Proteobacteria | Betaproteobacteria | Burkholderiales | Oxalobacteraceae | sf_1 | 7968 | U49757.2 | Oxalobacter formigenes str. OXB ovinen rumen |
| Bacteria | Proteobacteria | Gammaproteobacteria | Pseudomonadales | Pseudomonadaceae | sf_1 | 8508 | AF530073.1 | Pseudomonas citronellolis str. TERIDB26 |
| Bacteria | Proteobacteria | Gammaproteobacteria | Pseudomonadales | Pseudomonadaceae | sf_1 | 9219 | AF181576.1 | Pseudomonas cf. monteilii 9 |
| Bacteria | Proteobacteria | Gammaproteobacteria | Unclassified | Unclassified | sf_3 | 8714 | AB021372.1 | Marinobacter hydrocarbonoclasticus str. ATCC 27132T |
| Bacteria | Proteobacteria | Gammaproteobacteria | Enterobacteriales | Enterobacteriaceae | sf_1 | 8173 | Z76752.1 | Photorhabdus asymbiotica str. ATCC 43949 |
| Bacteria | Proteobacteria | Deltaproteobacteria | Unclassified | Unclassified | sf_9 | 9798 | AJ519663.1 | uranium mill tailings soil sample clone GuBH2-AD/TzT-67 proteobacterium |
| Bacteria | Proteobacteria | Deltaproteobacteria | Desulfuromonadales | Unclassified | sf_1 | 10076 | AF407700.1 | Great Artesian Basin clone G13 |
| Bacteria | Proteobacteria | Deltaproteobacteria | Desulfobacterales | Desulfobulbaceae | sf_1 | 9734 | AF449229.1 | Riftia pachyptila's tube clone R103-B13 |
| Bacteria | Actinobacteria | Actinobacteria | Actinomycetales | Streptosporangiaceae | sf_1 | 1158 | X89947.1 | Streptosporangium roseum str. DSM43021T |
| Bacteria | Actinobacteria | Actinobacteria | Actinomycetales | Microbacteriaceae | sf_1 | 1106 |  | |
| Bacteria | Firmicutes | Clostridia | Clostridiales | Peptostreptococcaceae | sf_5 | 619 | AY217429.1 | TCE-dechlorinating microbial community clone 1G |
| Bacteria | Firmicutes | Bacilli | Lactobacillales | Unclassified | sf_1 | 3289 | AJ302648.1 | Isobaculum melis CCUG 37660T |
| Bacteria | Firmicutes | Clostridia | Clostridiales | Clostridiaceae | sf_12 | 4551 | X78070.1 | Clostridium acetobutylicum str. ATCC 824 (T) |
| Bacteria | Unclassified | Unclassified | Unclassified | Unclassified | sf_160 | 333 |  | |
| Bacteria | Spirochaetes | Spirochaetes | Spirochaetales | Spirochaetaceae | sf_1 | 6506 | AB088911.1 | termite gut homogenate clone Rs-J58 sp. |
| Bacteria | Proteobacteria | Alphaproteobacteria | Ellin314/wr0007 | Unclassified | sf_1 | 7222 | AF407727.1 | Great Artesian Basin clone B79 |
| Bacteria | Proteobacteria | Gammaproteobacteria | Symbionts | Unclassified | sf_1 | 9128 | X95229.1 | Lucina nassula gill symbiont |
| Bacteria | Proteobacteria | Gammaproteobacteria | Pseudomonadales | Pseudomonadaceae | sf_1 | 8553 | AB046997.1 | Pseudomonas fulva str. IAM 1587 |
| Bacteria | Proteobacteria | Gammaproteobacteria | Alteromonadales | Alteromonadaceae | sf_1 | 8374 | AB076560.1 | Agarivorans albus str. MKT 89 |
| Bacteria | Proteobacteria | Deltaproteobacteria | Myxococcales | Unclassified | sf_1 | 10259 |  | |
| Bacteria | Proteobacteria | Deltaproteobacteria | Desulfobacterales | Desulfobacteraceae | sf_5 | 10031 | AY177795.1 | Antarctic sediment clone SB1_49 |
| Bacteria | Proteobacteria | Epsilonproteobacteria | Campylobacterales | Unclassified | sf_1 | 10407 | AF420342.1 | hydrothermal sediment clone AF420342 |
| Bacteria | Actinobacteria | Actinobacteria | Acidimicrobiales | Acidimicrobiaceae | sf_1 | 1090 |  | |
| Bacteria | Gemmatimonadetes | Unclassified | Unclassified | Unclassified | sf_5 | 1565 | AJ532728.1 | uranium mining waste pile clone JG34-KF-418 |
| Bacteria | OP9/JS1 | JS1 | Unclassified | Unclassified | sf_1 | 2489 | AY197377.1 | Guaymas Basin hydrothermal vent sediments clone B01R005 |
| Bacteria | Chloroflexi | Dehalococcoidetes | Unclassified | Unclassified | sf_1 | 2397 | AY093480.1 | deep marine sediment clone MB-C2-127 |
| Bacteria | Firmicutes | Clostridia | Clostridiales | Clostridiaceae | sf_12 | 3049 | Z69929.1 | Clostridium paradoxum str. DSM 7308T |
| Bacteria | Spirochaetes | Spirochaetes | Spirochaetales | Spirochaetaceae | sf_1 | 6490 | AB062823.1 | termite gut homogenate clone BCf4-14 |
| Bacteria | Proteobacteria | Alphaproteobacteria | Azospirillales | Magnetospirillaceae | sf_1 | 6922 | AY171615.1 | Dechlorospirillum sp. str. SN1 |
| Bacteria | Proteobacteria | Alphaproteobacteria | Consistiales | Caedibacteraceae | sf_5 | 6947 | AB089084.2 | termite gut homogenate clone Rs-B60 proteobacterium |
| Bacteria | Proteobacteria | Gammaproteobacteria | Unclassified | Unclassified | sf_3 | 8245 |  | |
| Bacteria | Proteobacteria | Gammaproteobacteria | Pseudomonadales | Pseudomonadaceae | sf_1 | 8777 | AB095005.1 | Pseudomonas sp. str. KNA6-5 |
| Bacteria | Proteobacteria | Gammaproteobacteria | Pseudomonadales | Pseudomonadaceae | sf_1 | 8852 | AF063219.1 | Pseudomonas stutzeri str. KC |
| Bacteria | Proteobacteria | Gammaproteobacteria | Pseudomonadales | Pseudomonadaceae | sf_1 | 9267 | AB001450.1 | Pseudomonas syringae pv. theae str. PT1 |
| Bacteria | Proteobacteria | Epsilonproteobacteria | Campylobacterales | Campylobacteraceae | sf_3 | 10397 | AB030592.1 | groundwater clone 1006 |
| Bacteria | Actinobacteria | Actinobacteria | Coriobacteriales | Coriobacteriaceae | sf_1 | 1459 | AB089077.1 | termite gut homogenate clone Rs-J59 bacterium |
| Bacteria | Actinobacteria | Actinobacteria | Actinomycetales | Micromonosporaceae | sf_1 | 2051 | D85479.1 | Couchioplanes subsp. caeruleus str. IFO13939 |
| Bacteria | Firmicutes | Clostridia | Clostridiales | Peptococc/Acidaminococc | sf_11 | 131 | AF349425.1 | pig feces clone |
| Bacteria | Firmicutes | Clostridia | Clostridiales | Peptococc/Acidaminococc | sf_11 | 181 | AF548373.1 | Allisonella histaminiformans str. MR2 |
| Bacteria | Firmicutes | Bacilli | Bacillales | Sporolactobacillaceae | sf_1 | 3747 | AB043848.1 | Bacillus sp. str. C-59-2 |
| Bacteria | Firmicutes | Bacilli | Lactobacillales | Aerococcaceae | sf_1 | 3386 | AF317386.1 | feedlot manure clone B87 |
| Bacteria | Firmicutes | Bacilli | Lactobacillales | Lactobacillaceae | sf_1 | 3696 | AY253657.1 | Lactobacillus kalixensis str. Kx127A2; LMG 22115T; DSM 16043T; CCUG 48459T |
| Bacteria | Firmicutes | Mollicutes | Entomoplasmatales | Entomoplasmataceae | sf_1 | 4074 | AF371522.1 | swine intestine clone p-2013-s959-5 |
| Bacteria | Verrucomicrobia | Verrucomicrobiae | Verrucomicrobiales | Unclassified | sf_3 | 11 | AF234711.1 | sludge clone H2 |
| Bacteria | Proteobacteria | Betaproteobacteria | Burkholderiales | Burkholderiaceae | sf_1 | 7720 | AY218566.1 | penguin droppings sediments clone KD1-79 |
| Bacteria | Proteobacteria | Gammaproteobacteria | Methylococcales | Methylococcaceae | sf_1 | 8243 | AF521582.1 | isolate str. IR |
| Bacteria | Proteobacteria | Gammaproteobacteria | Chromatiales | Unclassified | sf_1 | 9316 |  | |
| Bacteria | Proteobacteria | Gammaproteobacteria | Pseudomonadales | Moraxellaceae | sf_3 | 8604 | AB087260.2 | Moraxella oblonga str. IAM 14971 |
| Bacteria | Actinobacteria | Actinobacteria | Actinomycetales | Streptomycetaceae | sf_1 | 1771 | D63872.1 | Streptomyces setonii str. ATCC25497 |
| Bacteria | Actinobacteria | Actinobacteria | Actinomycetales | Microbacteriaceae | sf_1 | 1804 | AY244784.1 | Microbacterium resistens str. 2002-59119 |
| Bacteria | Firmicutes | Bacilli | Bacillales | Bacillaceae | sf_1 | 3383 |  | |
| Bacteria | Firmicutes | Mollicutes | Anaeroplasmatales | Erysipelotrichaceae | sf_3 | 4038 | AB031058.1 | Bulleidia moorei str. JCM 10647 RCA59-77 |
| Bacteria | Firmicutes | Clostridia | Clostridiales | Clostridiaceae | sf_12 | 4614 | AB093546.1 | Clostridium sp. str. JC3 |
| Bacteria | Firmicutes | Clostridia | Clostridiales | Lachnospiraceae | sf_5 | 4335 | AB088952.1 | termite gut homogenate clone Rs-N86 bacterium |
| Bacteria | Firmicutes | Clostridia | Clostridiales | Clostridiaceae | sf_12 | 4550 | AF371790.1 | swine intestine clone p-320-a3 |
| Bacteria | Firmicutes | Clostridia | Clostridiales | Clostridiaceae | sf_12 | 4582 | AF371783.1 | swine intestine clone p-2600-9F5 |
| Bacteria | Spirochaetes | Spirochaetes | Spirochaetales | Spirochaetaceae | sf_1 | 6568 | AJ419821.1 | Spirochaeta sp |
| Bacteria | Proteobacteria | Alphaproteobacteria | Unclassified | Unclassified | sf_6 | 6694 | AF523879.1 | forested wetland clone RCP2-92 |
| Bacteria | Proteobacteria | Alphaproteobacteria | Rhizobiales | Phyllobacteriaceae | sf_1 | 7009 |  | |
| Bacteria | Proteobacteria | Betaproteobacteria | Unclassified | Unclassified | sf_3 | 7765 |  | |
| Bacteria | Proteobacteria | Gammaproteobacteria | Acidithiobacillales | Acidithiobacillaceae | sf_1 | 9224 | AJ459804.1 | Acidithiobacillus albertensis str. DSM 14366 |
| Bacteria | Proteobacteria | Gammaproteobacteria | Chromatiales | Chromatiaceae | sf_1 | 8697 | AJ401211.1 | Thiococcus sp. AT2204 |
| Bacteria | Proteobacteria | Gammaproteobacteria | Unclassified | Unclassified | sf_3 | 8926 | AB099937.1 | inactive deep-sea hydrothermal vent chimneys clone IheB2-13 |
| Bacteria | Proteobacteria | Gammaproteobacteria | Pasteurellales | Pasteurellaceae | sf_1 | 8952 | M75068.1 | Actinobacillus lignieresii |
| Bacteria | Proteobacteria | Deltaproteobacteria | Unclassified | Unclassified | sf_9 | 9784 | AY177804.1 | Antarctic sediment clone LH5_30 |
| Bacteria | Actinobacteria | Actinobacteria | Actinomycetales | Streptomycetaceae | sf_3 | 1743 | AB026214.1 | Streptomyces scabiei str. DNK-G01 |
| Bacteria | Actinobacteria | Actinobacteria | Actinomycetales | Streptosporangiaceae | sf_1 | 1224 | X89941.1 | Acrocarpospora corrugata str. DSM43316T |
| Bacteria | Actinobacteria | Actinobacteria | Actinomycetales | Microbacteriaceae | sf_1 | 1667 | AB007415.1 | Microbacterium lacticum |
| Bacteria | Actinobacteria | Actinobacteria | Actinomycetales | Dietziaceae | sf_1 | 1872 | X79290.1 | Dietzia maris |
| Bacteria | Firmicutes | Bacilli | Bacillales | Bacillaceae | sf_1 | 3926 | X92160.1 | Lake Bogoria isolate 64B4 |
| Bacteria | Firmicutes | Bacilli | Lactobacillales | Lactobacillaceae | sf_1 | 3526 | AF401673.1 | Lactobacillus sakei |
| Bacteria | Firmicutes | Clostridia | Clostridiales | Clostridiaceae | sf_12 | 4359 | AB089047.1 | termite gut homogenate clone Rs-C69 bacterium |
| Bacteria | Bacteroidetes | Bacteroidetes | Bacteroidales | Bacteroidaceae | sf_12 | 5950 |  | |
| Bacteria | Bacteroidetes | Flavobacteria | Flavobacteriales | Flavobacteriaceae | sf_1 | 5267 | AF406541.1 | bacterioplankton clone AEGEAN_179 |
| Bacteria | Bacteroidetes | Flavobacteria | Flavobacteriales | Flavobacteriaceae | sf_1 | 6269 | AY080916.1 | acidic forest soil clone UC1 |
| Bacteria | Proteobacteria | Betaproteobacteria | Burkholderiales | Unclassified | sf_1 | 8035 |  | |
| Bacteria | Proteobacteria | Betaproteobacteria | Burkholderiales | Alcaligenaceae | sf_1 | 7737 | AY081977.1 | atrazine-catabolizing microbial presence methanol clone KRA30+06A |
| Bacteria | Proteobacteria | Gammaproteobacteria | SUP05 | Unclassified | sf_1 | 8654 | AB099939.1 | inactive deep-sea hydrothermal vent chimneys clone IheB2-31 |
| Bacteria | Proteobacteria | Gammaproteobacteria | Oceanospirillales | Alcanivoraceae | sf_1 | 9658 | AB053127.1 | Alcanivorax sp. str. Haw1 |
| Bacteria | Proteobacteria | Gammaproteobacteria | Pasteurellales | Pasteurellaceae | sf_1 | 9263 | M75067.1 | Actinobacillus capsulatus |
| Bacteria | Actinobacteria | Actinobacteria | Actinomycetales | Pseudonocardiaceae | sf_1 | 1343 | Z38017.1 | Saccharomonospora azurea str. M.Goodfel K161=NA128 (type st |
| Bacteria | OP3 | Unclassified | Unclassified | Unclassified | sf_3 | 1015 | AY013695.1 | polluted aquifer clone BVC56 |
| Bacteria | Bacteroidetes | Unclassified | Unclassified | Unclassified | sf_1 | 5745 |  | |
| Bacteria | Cyanobacteria | Cyanobacteria | Oscillatoriales | Unclassified | sf_1 | 5189 | AB039015.1 | Oscillatoria sancta str. PCC 7515 |
| Bacteria | Cyanobacteria | Cyanobacteria | Oscillatoriales | Unclassified | sf_1 | 5049 | AF420444.1 | Oscillatoria spongeliae str. 520bg |
| Bacteria | Cyanobacteria | Cyanobacteria | Chloroplasts | Chloroplasts | sf_11 | 5123 | AF289249.1 | Lepocinclis fusiformis str. ACOI 1025 |
| Bacteria | Proteobacteria | Alphaproteobacteria | Rickettsiales | Anaplasmataceae | sf_3 | 6803 | AY007547.1 | Wolbachia sp. Dlem16SWol |
| Bacteria | Proteobacteria | Alphaproteobacteria | Rickettsiales | Anaplasmataceae | sf_3 | 7481 | AF179630.1 | Wolbachia pipientis |
| Bacteria | Proteobacteria | Betaproteobacteria | Rhodocyclales | Rhodocyclaceae | sf_1 | 7925 | Y17591.1 | Thauera selenatis str. ATCC 55363T |
| Bacteria | Proteobacteria | Betaproteobacteria | Burkholderiales | Alcaligenaceae | sf_1 | 7768 | AF371864.1 | swine intestine clone p-861-a5 |
| Bacteria | Proteobacteria | Gammaproteobacteria | Chromatiales | Unclassified | sf_1 | 9282 |  | |
| Bacteria | Proteobacteria | Gammaproteobacteria | Pseudomonadales | Pseudomonadaceae | sf_1 | 9228 | AJ297767.1 | Pseudomonas stutzeri HY-105 |
| Bacteria | Proteobacteria | Gammaproteobacteria | Alteromonadales | Alteromonadaceae | sf_1 | 9111 | AF539773.1 | Pseudoalteromonas sp. str. E36 |
| Bacteria | Bacteroidetes | Sphingobacteria | Sphingobacteriales | Flexibacteraceae | sf_20 | 10311 | AJ431236.1 | Cytophaga sp. str. BHI60-57B |
| Bacteria | Firmicutes | Clostridia | Clostridiales | Lachnospiraceae | sf_5 | 3152 | AF385510.1 | tongue dorsa clone DO016 |
| Bacteria | Firmicutes | Clostridia | Clostridiales | Clostridiaceae | sf_12 | 4415 | AB089041.1 | termite gut homogenate clone Rs-K32 bacterium |
| Bacteria | Bacteroidetes | Flavobacteria | Flavobacteriales | Flavobacteriaceae | sf_1 | 5836 | U41348.1 | Capnocytophaga granulosa str. LMG 12119; FDC SD4 |
| Bacteria | Acidobacteria | Acidobacteria | Acidobacteriales | Acidobacteriaceae | sf_14 | 6425 | AF407719.1 | Great Artesian Basin clone B27 |
| Bacteria | Proteobacteria | Gammaproteobacteria | Pseudomonadales | Pseudomonadaceae | sf_1 | 8813 | AY150184.1 | Lyrodus pedicellatus symbiont |
| Bacteria | Proteobacteria | Gammaproteobacteria | Alteromonadales | Alteromonadaceae | sf_1 | 8222 |  | |
| Bacteria | Proteobacteria | Gammaproteobacteria | Pasteurellales | Pasteurellaceae | sf_1 | 9213 | AF224307.1 | Haemophilus quentini str. MCCM 02026 |
| Bacteria | Proteobacteria | Deltaproteobacteria | Syntrophobacterales | Syntrophobacteraceae | sf_1 | 10021 | AJ519630.1 | uranium mill tailings soil sample clone Sh765B-TzT-29 proteobacterium |
| Bacteria | Gemmatimonadetes | Unclassified | Unclassified | Unclassified | sf_5 | 10112 | AF432607.1 | forest soil clone NOS7.157WL |
| Bacteria | Gemmatimonadetes | Unclassified | Unclassified | Unclassified | sf_5 | 317 | AY218696.1 | penguin droppings sediments clone KD8-87 |
| Bacteria | Firmicutes | Clostridia | Clostridiales | Lachnospiraceae | sf_5 | 2784 | AJ408993.1 | human colonic clone HuCB12 |
| Bacteria | Firmicutes | Clostridia | Clostridiales | Lachnospiraceae | sf_5 | 2991 | AB034063.1 | rumen clone 3C3d-8 |
| Bacteria | Firmicutes | Clostridia | Clostridiales | Eubacteriaceae | sf_1 | 28 | AB088988.1 | termite gut homogenate clone Rs-H81 bacterium |
| Bacteria | NC10 | NC10-1 | Unclassified | Unclassified | sf_1 | 536 | AJ519669.1 | uranium mill tailings clone GuBH2-AD-8 |
| Bacteria | Bacteroidetes | Flavobacteria | Flavobacteriales | Flavobacteriaceae | sf_1 | 5423 | AF170754.1 | Aequorivita antarctica str. QSSC9-14 |
| Bacteria | Proteobacteria | Betaproteobacteria | Unclassified | Unclassified | sf_3 | 7974 |  | |
| Bacteria | Proteobacteria | Deltaproteobacteria | Myxococcales | Polyangiaceae | sf_3 | 9755 | AF382126.1 | bacterioplankton clone ZA3704c |
| Bacteria | Proteobacteria | Deltaproteobacteria | Desulfuromonadales | Geobacteraceae | sf_1 | 10171 |  | |
| Bacteria | Proteobacteria | Deltaproteobacteria | Syntrophobacterales | Syntrophobacteraceae | sf_1 | 9845 | AJ518800.1 | uranium mining waste pile clone JG37-AG-128 proteobacterium |
| Bacteria | Actinobacteria | Actinobacteria | Unclassified | Unclassified | sf_2 | 1233 |  | |
| Bacteria | Actinobacteria | Actinobacteria | Actinomycetales | Streptomycetaceae | sf_1 | 1544 | AB022872.1 | Kitasatospora cystarginea str. IFO14836T |
| Bacteria | Actinobacteria | Actinobacteria | Actinomycetales | Streptomycetaceae | sf_1 | 1617 | AF423260.1 | soil clone 41-1 |
| Bacteria | Firmicutes | Bacilli | Bacillales | Bacillaceae | sf_1 | 3688 | AY167821.1 | Bacillus sp. str. SAFN-006 |
| Bacteria | Bacteroidetes | Sphingobacteria | Sphingobacteriales | Crenotrichaceae | sf_11 | 6050 | AF527580.1 | EBPR sludge lab scale clone LPB08 |
| Bacteria | Planctomycetes | Planctomycetacia | Planctomycetales | Pirellulae | sf_3 | 4677 | BX294863.1 | aerobic basin clone CY0ARA032A03 |
| Bacteria | Cyanobacteria | Cyanobacteria | Nostocales | Unclassified | sf_1 | 5057 | AJ781148.1 | Nodularia sphaerocarpa str. UTEX B 2093 |
| Bacteria | Spirochaetes | Spirochaetes | Spirochaetales | Spirochaetaceae | sf_1 | 6458 | AB084961.1 | termite gut clone NkS34 |
| Bacteria | Spirochaetes | Spirochaetes | Spirochaetales | Spirochaetaceae | sf_1 | 6562 | AF523928.1 | forested wetland clone RCP1-96 |
| Bacteria | Proteobacteria | Betaproteobacteria | Burkholderiales | Alcaligenaceae | sf_1 | 7788 | AY081994.1 | atrazine-catabolizing microbial absence methanol clone KRA30-58 |
| Bacteria | Proteobacteria | Betaproteobacteria | Burkholderiales | Oxalobacteraceae | sf_1 | 7878 | AY250094.1 | napthalene-contaminated sediment clone 29 |
| Bacteria | Proteobacteria | Betaproteobacteria | MND1 clone group | Unclassified | sf_1 | 7993 | AF423222.1 | soil clone 1326-2 |
| Bacteria | Proteobacteria | Deltaproteobacteria | Desulfuromonadales | Desulfuromonaceae | sf_1 | 10020 | AJ519664.1 | uranium mill tailings soil sample clone GuBH2-AG-114 proteobacterium |
| Bacteria | Gemmatimonadetes | Unclassified | Unclassified | Unclassified | sf_5 | 1238 | AB072735.1 | Gemmatimonas aurantiaca |
| Bacteria | Actinobacteria | Actinobacteria | Actinomycetales | Streptomycetaceae | sf_1 | 1786 | X79851.1 | Streptomyces bikiniensis str. DSM40581 |
| Bacteria | Actinobacteria | Actinobacteria | Bifidobacteriales | Bifidobacteriaceae | sf_1 | 2040 | AF275881.1 | Bifidobacterium adolescentis str. E-981074T |
| Bacteria | Chloroflexi | Unclassified | Unclassified | Unclassified | sf_1 | 2534 | AF507693.1 | forest soil clone S085 |
| Bacteria | Firmicutes | Bacilli | Lactobacillales | Aerococcaceae | sf_1 | 3519 | AF016390.1 | Granulicatella elegans str. DSM 11693; B1333 |
| Bacteria | Firmicutes | Bacilli | Lactobacillales | Enterococcaceae | sf_1 | 3680 | X75752.1 | Melissococcus plutonius str. NCDO 2440 |
| Bacteria | Deinococcus-Thermus | Unclassified | Unclassified | Unclassified | sf_2 | 637 | AF513964.1 | hypersaline pond clone LA7-B27N |
| Bacteria | Bacteroidetes | Bacteroidetes | Bacteroidales | Unclassified | sf_15 | 6046 | AJ488097.1 | chlorobenzene-degrading consortium clone IIIB-1 |
| Bacteria | Proteobacteria | Alphaproteobacteria | Consistiales | Caedibacteraceae | sf_4 | 7157 | AF533506.1 | acid mine drainage clone ASL45 |
| Bacteria | Proteobacteria | Betaproteobacteria | Burkholderiales | Alcaligenaceae | sf_1 | 7932 | AJ278451.1 | Achromobacter subsp. denitrificans str. DSM 30026 (T) |
| Bacteria | Proteobacteria | Betaproteobacteria | Burkholderiales | Oxalobacteraceae | sf_1 | 8013 | AF236013.1 | isolate str. A1020 |
| Bacteria | Proteobacteria | Gammaproteobacteria | Alteromonadales | Alteromonadaceae | sf_1 | 8318 | AY207502.1 | Aestuariibacter salexigens str. JC2042 |
| Bacteria | Proteobacteria | Gammaproteobacteria | Pasteurellales | Pasteurellaceae | sf_1 | 8876 | AF053894.1 | Mannheimia sp. R19.2 str. R19.2; CCUG 38463 R19.2 |
| Bacteria | Proteobacteria | Deltaproteobacteria | Syntrophobacterales | Syntrophobacteraceae | sf_1 | 9864 | AJ518801.1 | uranium mining waste pile clone JG37-AG-133 proteobacterium |
| Bacteria | Actinobacteria | Actinobacteria | Acidimicrobiales | Unclassified | sf_1 | 1666 |  | |
| Bacteria | Actinobacteria | Actinobacteria | Unclassified | Unclassified | sf_1 | 1676 |  | |
| Bacteria | Actinobacteria | Actinobacteria | Actinomycetales | Micromonosporaceae | sf_1 | 1910 | AB013495.2 | Actinoplanes capillaceus str. K95-5561 |
| Bacteria | Firmicutes | Bacilli | Bacillales | Bacillaceae | sf_1 | 3589 | AF519468.1 | Bacillus senegalensis str. RS8; CIP 106 669 |
| Bacteria | Proteobacteria | Deltaproteobacteria | Desulfobacterales | Nitrospinaceae | sf_2 | 594 | AJ296568.1 | uranium mining mill tailing clone GR-296.II.52 GR-296.I.52 |
| Bacteria | Proteobacteria | Deltaproteobacteria | Desulfuromonadales | Geobacteraceae | sf_1 | 482 | AF529120.1 | trichloroethene-contaminated site clone FTLM205 proteobacterium |
| Bacteria | Chloroflexi | Anaerolineae | Unclassified | Unclassified | sf_9 | 72 | AY221035.1 | sediments collected at Charon's Cascade near Echo River October 2000 clone CCD21 |
| Bacteria | Acidobacteria | Acidobacteria | Acidobacteriales | Acidobacteriaceae | sf_14 | 6366 | AJ292578.1 | PCB-polluted soil clone WD228 |
| Bacteria | Acidobacteria | Acidobacteria | Acidobacteriales | Acidobacteriaceae | sf_14 | 6424 | AF524859.1 | sphagnum peat bog clone K-5b10 |
| Bacteria | Spirochaetes | Spirochaetes | Spirochaetales | Spirochaetaceae | sf_3 | 6558 | AF166259.1 | Spironema culicis str. BR91 |
| Bacteria | Proteobacteria | Alphaproteobacteria | Bradyrhizobiales | Xanthobacteraceae | sf_1 | 6660 | D11342.1 | Azorhizobium caulinodans str. ORS 571 |
| Bacteria | Proteobacteria | Betaproteobacteria | Rhodocyclales | Rhodocyclaceae | sf_1 | 8156 | AY098637.2 | industrial-phenol-degrading community clone MM1 sp. |
| Bacteria | Proteobacteria | Betaproteobacteria | Procabacteriales | Procabacteriaceae | sf_1 | 8136 | AF177426.1 | Acanthamoeba sp. UWC6 symbiont |
| Bacteria | Proteobacteria | Gammaproteobacteria | Ellin307/WD2124 | Unclassified | sf_1 | 8532 |  | |
| Bacteria | Proteobacteria | Gammaproteobacteria | Alteromonadales | Alteromonadaceae | sf_1 | 9292 |  | |
| Bacteria | Proteobacteria | Gammaproteobacteria | Alteromonadales | Alteromonadaceae | sf_1 | 8643 | AY771715.1 | Pseudoalteromonas porphyrae str. S2-65 |
| Bacteria | Proteobacteria | Gammaproteobacteria | Alteromonadales | Alteromonadaceae | sf_1 | 8579 | AJ416756.1 | Psychromonas profunda str. 2825 |
| Bacteria | Proteobacteria | Deltaproteobacteria | Desulfobacterales | Desulfobulbaceae | sf_1 | 10047 | AY355301.2 | epibiontic clone C11-D3 |
| Bacteria | Proteobacteria | Deltaproteobacteria | Syntrophobacterales | Syntrophaceae | sf_3 | 9665 | X85132.1 | Syntrophus gentianae str. HQgoe1 |
| Bacteria | Proteobacteria | Epsilonproteobacteria | Campylobacterales | Helicobacteraceae | sf_3 | 10411 | AB089112.2 | termite gut homogenate clone Rs-P71 proteobacterium |
| Bacteria | Actinobacteria | Actinobacteria | Actinomycetales | Streptomycetaceae | sf_1 | 1354 | X80825.1 | Streptomyces subrutilus str. DSM 40445 |
| Bacteria | Actinobacteria | Actinobacteria | Bifidobacteriales | Bifidobacteriaceae | sf_1 | 1967 | D86187.1 | Bifidobacterium pseudocatenulatum str. JCM1200 |
| Bacteria | Firmicutes | Clostridia | Clostridiales | Lachnospiraceae | sf_5 | 3075 | AJ312385.1 | Roseburia intestinalis str. L1-82 |
| Bacteria | Firmicutes | Bacilli | Bacillales | Bacillaceae | sf_1 | 3517 | AF500007.1 | Planococcus maritimus str. TF-9 |
| Bacteria | Firmicutes | Clostridia | Clostridiales | Lachnospiraceae | sf_5 | 4539 | AB089046.1 | termite gut homogenate clone Rs-C61 bacterium |
| Bacteria | Firmicutes | Clostridia | Clostridiales | Clostridiaceae | sf_12 | 4610 | AF127024.1 | Clostridium putrefaciens str. DSM 1291 |
| Bacteria | Proteobacteria | Alphaproteobacteria | Unclassified | Unclassified | sf_2 | 6697 | AB089090.1 | termite gut homogenate clone Rs-D84 proteobacterium |
| Bacteria | Proteobacteria | Alphaproteobacteria | Unclassified | Unclassified | sf_6 | 7572 |  | |
| Bacteria | Proteobacteria | Gammaproteobacteria | Oceanospirillales | Halomonadaceae | sf_1 | 8514 | AJ295144.1 | Chromohalobacter israelensis str. ATCC 43985 T |
| Bacteria | Proteobacteria | Gammaproteobacteria | Pasteurellales | Pasteurellaceae | sf_1 | 9533 | AF224299.1 | Haemophilus segnis str. MCCM 00337 |
| Bacteria | Proteobacteria | Gammaproteobacteria | Enterobacteriales | Enterobacteriaceae | sf_1 | 8286 | AF476102.1 | Cyphonococcus alpinus symbiont |
| Bacteria | Proteobacteria | Deltaproteobacteria | Syntrophobacterales | Syntrophobacteraceae | sf_1 | 9731 | AJ518795.1 | uranium mining waste pile clone JG37-AG-90 proteobacterium |
| Bacteria | Proteobacteria | Deltaproteobacteria | Desulfobacterales | Desulfobacteraceae | sf_5 | 9940 | AY177800.1 | Antarctic sediment clone SB2_56 |
| Bacteria | Bacteroidetes | Bacteroidetes | Bacteroidales | Unclassified | sf_15 | 5957 | AJ441217.1 | hydrothermal vent polychaete mucous clone P. palm C/A 20 |
| Bacteria | Bacteroidetes | Sphingobacteria | Sphingobacteriales | Crenotrichaceae | sf_11 | 5888 | AY218661.1 | penguin droppings sediments clone KD9-169 |
| Bacteria | Spirochaetes | Spirochaetes | Spirochaetales | Spirochaetaceae | sf_1 | 6491 | AB062840.1 | termite gut homogenate clone BCf8-03 |
| Bacteria | Proteobacteria | Gammaproteobacteria | Legionellales | Coxiellaceae | sf_3 | 9444 | AF523974.1 | forested wetland clone FW23 |
| Bacteria | Proteobacteria | Gammaproteobacteria | Pseudomonadales | Pseudomonadaceae | sf_1 | 8474 | AJ534672.1 | ground water deep-well injection disposal site radioactive wastes Tomsk-7 clone S15A-MN7 proteobacterium |
| Bacteria | Actinobacteria | Actinobacteria | Actinomycetales | Frankiaceae | sf_1 | 1286 | AJ408875.1 | Frankia sp. Sn5-8 |
| Bacteria | Firmicutes | Bacilli | Bacillales | Bacillaceae | sf_1 | 283 | Z26926.1 | Geobacillus thermocatenulatus str. DSM 730 |
| Bacteria | Firmicutes | Mollicutes | Acholeplasmatales | Acholeplasmataceae | sf_1 | 3976 |  | |
| Bacteria | NC10 | NC10-1 | Unclassified | Unclassified | sf_1 | 452 | AY177763.1 | vadose clone 5G01 |
| Bacteria | Cyanobacteria | Cyanobacteria | Chloroplasts | Chloroplasts | sf_5 | 5192 | AF244551.1 | Cycas revoluta |
| Bacteria | Proteobacteria | Alphaproteobacteria | Acetobacterales | Acetobacteraceae | sf_1 | 7529 | AJ012698.1 | Gluconacetobacter europaeus str. ZIM B028 V3 |
| Bacteria | Proteobacteria | Alphaproteobacteria | Rickettsiales | Anaplasmataceae | sf_3 | 6628 | U23709.1 | Wolbachia pipientis |
| Bacteria | Proteobacteria | Gammaproteobacteria | Alteromonadales | Alteromonadaceae | sf_1 | 9236 | AF114501.1 | attached marine recovered surface clone 18 proteobacterium |
| Bacteria | Proteobacteria | Gammaproteobacteria | Pasteurellales | Pasteurellaceae | sf_1 | 8848 | AF227858.1 | str. 86355 |
| Bacteria | Proteobacteria | Deltaproteobacteria | Myxococcales | Myxococcaceae | sf_1 | 10358 | AJ233917.1 | Myxococcus fulvus str. Mx f2 |
| Bacteria | Proteobacteria | Deltaproteobacteria | Unclassified | Unclassified | sf_9 | 9876 | AY093471.1 | deep marine sediment clone MB-B2-106 |
| Bacteria | Proteobacteria | Deltaproteobacteria | AMD clone group | Unclassified | sf_1 | 9678 | AF523883.1 | coal effluent wetland clone RCP185 |
| Bacteria | Actinobacteria | Actinobacteria | Actinomycetales | Promicromonosporaceae | sf_1 | 1711 | AJ272024.1 | Promicromonospora sukumoe str. DSM 44121 |
| Bacteria | Actinobacteria | Actinobacteria | Actinomycetales | Micrococcaceae | sf_1 | 1889 | AB094464.1 | Citricoccus sp. str. 2216.25.22 |
| Bacteria | Actinobacteria | Actinobacteria | Actinomycetales | Mycobacteriaceae | sf_1 | 1175 | AJ243481.1 | Mycobacterium cf. xenopi 'Hymi_Wue Tb_939/99' str. Hymi_Wue Tb_939/99 |
| Bacteria | Caldithrix | Unclassified | Caldithrales | Caldithraceae | sf_1 | 2384 | AF286032.1 | saltmarsh clone LCP-89 |
| Bacteria | Firmicutes | Clostridia | Clostridiales | Lachnospiraceae | sf_5 | 3088 | X73449.1 | Clostridium sphenoides str. DSM 632 |
| Bacteria | Firmicutes | Bacilli | Lactobacillales | Carnobacteriaceae | sf_1 | 3536 |  | |
| Bacteria | Acidobacteria | Unclassified | Unclassified | Unclassified | sf_1 | 4222 | AF523991.1 | forested wetland clone FW105 |
| Bacteria | Firmicutes | Clostridia | Clostridiales | Clostridiaceae | sf_12 | 4638 |  | |
| Bacteria | Firmicutes | Clostridia | Clostridiales | Clostridiaceae | sf_12 | 4180 | AB089028.1 | termite gut homogenate clone Rs-M23 bacterium |
| Bacteria | Firmicutes | Clostridia | Clostridiales | Clostridiaceae | sf_12 | 4339 | U51843.1 | Clostridium chauvoei str. ATCC 10092T |
| Bacteria | Firmicutes | Clostridia | Clostridiales | Clostridiaceae | sf_12 | 4589 | X68176.1 | Clostridium butyricum str. ATCC43755 |
| Bacteria | Nitrospira | Nitrospira | Nitrospirales | Nitrospiraceae | sf_1 | 984 | AJ519405.1 | uranium mining waste pile clone JG37-AG-131 sp. |
| Bacteria | Bacteroidetes | Bacteroidetes | Bacteroidales | Unclassified | sf_15 | 6233 | AY134906.1 | oral periodontitis clone FX069 |
| Bacteria | Chloroflexi | Anaerolineae | Unclassified | Unclassified | sf_9 | 576 | AJ306746.2 | DCP-dechlorinating consortium clone SHA-36 |
| Bacteria | Unclassified | Unclassified | Unclassified | Unclassified | sf_160 | 651 |  | |
| Bacteria | Acidobacteria | Acidobacteria | Acidobacteriales | Acidobacteriaceae | sf_14 | 6350 | AF498719.1 | soil isolate Ellin337 |
| Bacteria | Proteobacteria | Alphaproteobacteria | Rhizobiales | Phyllobacteriaceae | sf_1 | 7497 | AJ294416.1 | Pseudaminobacter salicylatoxidans str. KTC001 |
| Bacteria | Proteobacteria | Alphaproteobacteria | Rickettsiales | Anaplasmataceae | sf_3 | 6648 | AF035160.1 | Wolbachia sp |
| Bacteria | Proteobacteria | Alphaproteobacteria | Rickettsiales | Anaplasmataceae | sf_3 | 6908 | M85267.1 | Rhinocyllus conicus endosymbiont |
| Bacteria | Proteobacteria | Gammaproteobacteria | aquatic clone group | Unclassified | sf_1 | 9246 | AY221036.1 | Mammoth Cave sediment clone CCD24 |
| Bacteria | Proteobacteria | Gammaproteobacteria | Pseudomonadales | Moraxellaceae | sf_3 | 9428 | AF143841.1 | hydrocarbon-degrading consortium clone AF2-1D |
| Bacteria | Proteobacteria | Gammaproteobacteria | Alteromonadales | Alteromonadaceae | sf_1 | 9416 | AF539786.1 | marine isolate str. R8 |
| Bacteria | Proteobacteria | Deltaproteobacteria | Myxococcales | Polyangiaceae | sf_3 | 9900 | AF280857.1 | bioreactor clone mle1-27 |
| Bacteria | Proteobacteria | Epsilonproteobacteria | Campylobacterales | Helicobacteraceae | sf_3 | 10590 | AB089113.1 | termite gut homogenate clone Rs-H40 proteobacterium |
| Bacteria | Actinobacteria | Actinobacteria | Actinomycetales | Actinomycetaceae | sf_1 | 2039 | AJ421779.1 | Actinomyces cardiffensis str. CCUG 44997 |
| Bacteria | Spirochaetes | Spirochaetes | Spirochaetales | Spirochaetaceae | sf_1 | 6526 | AY230217.1 | Treponema sp. str. 7CPL208 |
| Bacteria | Spirochaetes | Spirochaetes | Spirochaetales | Spirochaetaceae | sf_1 | 6508 | X89050.1 | termite hindgut clone mpsp2 |
| Bacteria | Proteobacteria | Alphaproteobacteria | Rhodobacterales | Rhodobacteraceae | sf_1 | 7433 | AF359537.1 | Scrippsiella trochoidea NEPCC 15 |
| Bacteria | Proteobacteria | Gammaproteobacteria | Alteromonadales | Shewanellaceae | sf_1 | 8641 | AJ252022.1 | Moritella abyssi str. 2693 |
| Bacteria | Proteobacteria | Gammaproteobacteria | Pasteurellales | Pasteurellaceae | sf_1 | 8614 | AF359941.1 | Acidithiobacillus thiooxidans str. KCTC 8928P |
| Bacteria | TM6 | Unclassified | Unclassified | Unclassified | sf_1 | 9803 | AF507715.1 | forest soil clone S1204 |
| Bacteria | Firmicutes | Bacilli | Lactobacillales | Lactobacillaceae | sf_1 | 3885 | M58834.1 | Pediococcus pentosaceus |
| Bacteria | Firmicutes | Mollicutes | Acholeplasmatales | Acholeplasmataceae | sf_1 | 4044 |  | |
| Bacteria | Firmicutes | Clostridia | Clostridiales | Clostridiaceae | sf_12 | 4555 | M59113.1 | Clostridium tyrobutyricum |
| Bacteria | WS3 | Unclassified | Unclassified | Unclassified | sf_3 | 95 | AJ535231.1 | marine sediment above hydrate ridge clone Hyd24-32 |
| Bacteria | Bacteroidetes | Sphingobacteria | Sphingobacteriales | Flexibacteraceae | sf_19 | 5563 | AB073573.2 | Cytophaga sp. I-545 |
| Bacteria | Acidobacteria | Solibacteres | Unclassified | Unclassified | sf_1 | 6357 | AY118153.1 | anaerobic benzene-degrading clone Cart-N4 |
| Bacteria | Proteobacteria | Alphaproteobacteria | Bradyrhizobiales | Unclassified | sf_1 | 7199 | AJ532683.1 | uranium mill tailings clone Gitt-KF-194 |
| Bacteria | Proteobacteria | Alphaproteobacteria | Sphingomonadales | Sphingomonadaceae | sf_15 | 7035 | Y09639.1 | Sphingomonas asaccharolytica str. IFO 10564-T |
| Bacteria | Proteobacteria | Betaproteobacteria | Burkholderiales | Ralstoniaceae | sf_1 | 8110 | AF085226.1 | Wautersia paucula str. LMG 3413 |
| Bacteria | Proteobacteria | Gammaproteobacteria | Enterobacteriales | Enterobacteriaceae | sf_1 | 8607 | AF476100.1 | Amonostherium lichtensioides symbiont |
| Bacteria | Actinobacteria | Actinobacteria | Actinomycetales | Thermomonosporaceae | sf_1 | 1546 | AJ420137.1 | Actinomadura fulvescens str. DSM 43923T |
| Bacteria | Firmicutes | Clostridia | Clostridiales | Lachnospiraceae | sf_5 | 2693 | AB008552.1 | ruminantium str. GA195 |
| Bacteria | Firmicutes | Bacilli | Bacillales | Paenibacillaceae | sf_1 | 3641 | AJ313027.1 | Brevibacillus sp. MN 47.2a |
| Bacteria | Firmicutes | Mollicutes | Acholeplasmatales | Acholeplasmataceae | sf_1 | 4046 | L33735.1 | Pigeon pea witches'-broom mycoplasma-like organism |
| Bacteria | Bacteroidetes | Bacteroidetes | Bacteroidales | Prevotellaceae | sf_1 | 5769 | AB081578.1 | Bacteroidaceae str. A42 |
| Bacteria | marine group A | mgA-1 | Unclassified | Unclassified | sf_1 | 6454 | U34043.1 | marine clone SAR406 |
| Bacteria | Spirochaetes | Spirochaetes | Spirochaetales | Spirochaetaceae | sf_1 | 6571 | AJ458946.1 | Mixotricha paradoxa is flagellate hindgut Mastotermes darwiniensis clone mp4 of |
| Bacteria | Proteobacteria | Betaproteobacteria | Burkholderiales | Alcaligenaceae | sf_1 | 7992 | AJ509012.1 | Alcaligenes faecalis 5659-H |
| Bacteria | Proteobacteria | Betaproteobacteria | Burkholderiales | Alcaligenaceae | sf_1 | 8062 | AJ277742.1 | Brackiella oedipodis str. LMG 1945 R8846 |
| Bacteria | Proteobacteria | Gammaproteobacteria | Xanthomonadales | Xanthomonadaceae | sf_3 | 9320 | AJ318204.1 | Waste-gas biofilter clone BIyi3 |
| Bacteria | Proteobacteria | Gammaproteobacteria | Alteromonadales | Alteromonadaceae | sf_1 | 8975 | AB016268.1 | Alteromonas sp. str. NIBH P1M3 |
| Bacteria | Proteobacteria | Epsilonproteobacteria | Campylobacterales | Unclassified | sf_1 | 10427 | AY672515.1 | hydrothermal vent 9 degrees North East Rise Pacific Ocean clone CH3_17_BAC_16SrRNA_9N_EPR |
| Bacteria | Proteobacteria | Epsilonproteobacteria | Campylobacterales | Campylobacteraceae | sf_3 | 10446 |  | |
| Bacteria | Proteobacteria | Epsilonproteobacteria | Campylobacterales | Campylobacteraceae | sf_3 | 10540 | AF550655.1 | Campylobacter showae str. LMG 12636 |
| Bacteria | Actinobacteria | Actinobacteria | Actinomycetales | Dermabacteraceae | sf_1 | 2053 | X91033.1 | Brachybacterium nesterenkovii str. DSM 9573 |
| Bacteria | Unclassified | Unclassified | Unclassified | Unclassified | sf_156 | 4291 | AF507892.1 | Mono Lake at depth 35m station 6 July 2000 clone ML635J-21 G+C |
| Bacteria | Firmicutes | Clostridia | Clostridiales | Clostridiaceae | sf_12 | 4598 | AB161372.1 | Clostridium sardiniense str. DSM 600 |
| Bacteria | Bacteroidetes | Unclassified | Unclassified | Unclassified | sf_4 | 5703 |  | |
| Bacteria | Bacteroidetes | Sphingobacteria | Sphingobacteriales | Sphingobacteriaceae | sf_1 | 6158 | AF538748.1 | municipal wastewater treatment bioreactor isolate str. CAGY10 |
| Bacteria | Bacteroidetes | Sphingobacteria | Sphingobacteriales | Crenotrichaceae | sf_11 | 5654 | AB088636.1 | Saprospira grandis str. ATCC23116 |
| Bacteria | Chloroflexi | Anaerolineae | Chloroflexi-1a | Unclassified | sf_1 | 106 | AJ306798.1 | DCP-dechlorinating consortium clone SHD-231 |
| Bacteria | Planctomycetes | Planctomycetacia | Planctomycetales | Anammoxales | sf_4 | 4694 | AF418968.1 | USA: Colorado Fort collins Horsetooth Reservoir clone HT2F11 |
| Bacteria | TM7 | Unclassified | Unclassified | Unclassified | sf_1 | 5177 |  | |
| Bacteria | Acidobacteria | Solibacteres | Unclassified | Unclassified | sf_1 | 6367 | AF529104.1 | TCE-contaminated site clone FTL227 |
| Bacteria | Acidobacteria | Acidobacteria | Acidobacteriales | Acidobacteriaceae | sf_14 | 6410 |  | |
| Bacteria | Proteobacteria | Alphaproteobacteria | Acetobacterales | Unclassified | sf_1 | 7028 |  | |
| Bacteria | Proteobacteria | Betaproteobacteria | Nitrosomonadales | Nitrosomonadaceae | sf_1 | 7770 | BX321856.1 | Nitrosomonas europaea str. ATCC 19718 |
| Bacteria | Proteobacteria | Gammaproteobacteria | Legionellales | Coxiellaceae | sf_3 | 9198 | AJ295651.1 | uranium mining waste pile clone KF-JG30-B15 KF-JG30-B15 |
| Bacteria | Proteobacteria | Deltaproteobacteria | Desulfobacterales | Desulfobacteraceae | sf_5 | 9800 | AF523960.1 | forested wetland clone FW57 |
| Bacteria | Actinobacteria | Actinobacteria | Actinomycetales | Nocardiaceae | sf_1 | 1142 |  | |
| Bacteria | Firmicutes | Clostridia | Clostridiales | Peptococc/Acidaminococc | sf_11 | 863 | AY162469.1 | Dialister invisus str. E7_25 |
| Bacteria | Firmicutes | Bacilli | Bacillales | Sporolactobacillaceae | sf_1 | 3365 | AF454300.1 | Bacillus sp. clone ML615J-19 |
| Bacteria | Firmicutes | Bacilli | Bacillales | Bacillaceae | sf_1 | 3424 | AJ532701.1 | uranium mill tailings clone Gitt-KF-76 |
| Bacteria | Acidobacteria | Acidobacteria-6 | Unclassified | Unclassified | sf_1 | 1049 | AF013534.2 | soil clone C112 |
| Bacteria | Bacteroidetes | Flavobacteria | Flavobacteriales | Flavobacteriaceae | sf_1 | 5401 | AF543295.1 | Capnocytophaga gingivalis str. ChDC OS45 |
| Bacteria | Proteobacteria | Betaproteobacteria | Nitrosomonadales | Nitrosomonadaceae | sf_1 | 8145 | AY123795.1 | Nitrosomonas eutropha str. Nm57 |
| Bacteria | Proteobacteria | Betaproteobacteria | Burkholderiales | Comamonadaceae | sf_1 | 7856 | AY169431.1 | Variovorax paradoxus |
| Bacteria | Proteobacteria | Betaproteobacteria | Burkholderiales | Oxalobacteraceae | sf_1 | 7921 | AY593479.1 | Collimonas fungivorans str. Ter331 |
| Bacteria | Proteobacteria | Gammaproteobacteria | Xanthomonadales | Xanthomonadaceae | sf_3 | 9286 | AJ131912.1 | Stenotrophomonas maltophilia str. LMG 11104 |
| Bacteria | Proteobacteria | Gammaproteobacteria | Alteromonadales | Alteromonadaceae | sf_1 | 8172 | AB055791.1 | Pseudoalteromonas sp. str. Bdeep-1 |
| Bacteria | Proteobacteria | Gammaproteobacteria | Alteromonadales | Alteromonadaceae | sf_1 | 8336 | AF237977.1 | Alteromonas sp. str. MS23 |
| Bacteria | Actinobacteria | Actinobacteria | Coriobacteriales | Coriobacteriaceae | sf_1 | 1800 | AJ534681.1 | ground water deep-well injection disposal site radioactive wastes Tomsk-7 clone S15A-MN100 |
| Bacteria | Actinobacteria | Actinobacteria | Acidimicrobiales | Acidimicrobiaceae | sf_1 | 1856 | AF523913.1 | forested wetland clone RCP2-105 |
| Bacteria | Actinobacteria | Actinobacteria | Actinomycetales | Micrococcaceae | sf_1 | 2063 | M59055.1 | Rothia dentocariosa str. ATCC 17931 |
| Bacteria | Firmicutes | Clostridia | Clostridiales | Peptococc/Acidaminococc | sf_11 | 534 | AJ488084.1 | chlorobenzene-degrading consortium clone IIA-26 |
| Bacteria | Firmicutes | Clostridia | Clostridiales | Clostridiaceae | sf_12 | 4187 | AF538854.1 | Clostridiales oral clone P4PB_122 P3 |
| Bacteria | Bacteroidetes | Bacteroidetes | Bacteroidales | Porphyromonadaceae | sf_1 | 5713 | X82823.1 | Porphyromonas catoniae str. ATCC 51270 |
| Bacteria | Bacteroidetes | Bacteroidetes | Bacteroidales | Prevotellaceae | sf_1 | 6239 | AF385512.1 | tongue dorsa clone DO033 |
| Bacteria | Bacteroidetes | Flavobacteria | Flavobacteriales | Flavobacteriaceae | sf_1 | 5473 |  | |
| Bacteria | Bacteroidetes | Flavobacteria | Flavobacteriales | Flavobacteriaceae | sf_1 | 5914 | AY771714.1 | Psychroserpens burtonensis str. S2-64 |
| Bacteria | Chloroflexi | Anaerolineae | Chloroflexi-1b | Unclassified | sf_2 | 789 | AF445676.1 | travertine hot spring clone SM1D10 |
| Bacteria | Proteobacteria | Betaproteobacteria | Neisseriales | Neisseriaceae | sf_1 | 7945 | AB074518.1 | Aquaspirillum serpens str. IAM 13944 |
| Bacteria | Proteobacteria | Betaproteobacteria | Burkholderiales | Ralstoniaceae | sf_1 | 7823 | AF312022.1 | Wautersia basilensis str. DSM 11853 |
| Bacteria | Proteobacteria | Gammaproteobacteria | SAR86 | Unclassified | sf_1 | 8962 | AF406526.1 | bacterioplankton clone AEGEAN_234 |
| Bacteria | Proteobacteria | Gammaproteobacteria | Alteromonadales | Alteromonadaceae | sf_1 | 9205 | AF354597.1 | marine clone Arctic96B-17 |
| Bacteria | Proteobacteria | Gammaproteobacteria | Alteromonadales | Pseudoalteromonadaceae | sf_1 | 9627 | AF218245.1 | Pseudoalteromonas sp |
| Bacteria | Proteobacteria | Deltaproteobacteria | Myxococcales | Polyangiaceae | sf_3 | 9735 | AJ518790.1 | uranium mining waste pile clone JG37-AG-15 proteobacterium |
| Bacteria | Proteobacteria | Deltaproteobacteria | Syntrophobacterales | Syntrophobacteraceae | sf_1 | 9661 | AJ306805.1 | DCP-dechlorinating consortium clone SHD-1 |
| Bacteria | Actinobacteria | BD2-10 group | Unclassified | Unclassified | sf_2 | 1652 | AY193208.1 | marine sediment clone Bol7 |
| Bacteria | Actinobacteria | Actinobacteria | Actinomycetales | Corynebacteriaceae | sf_1 | 1517 | X84446.1 | Corynebacterium xerosis str. DSM 20743 |
| Bacteria | Actinobacteria | Actinobacteria | Actinomycetales | Corynebacteriaceae | sf_1 | 1803 | AJ429234.1 | Corynebacterium spheniscorum str. CCUG 45512 |
| Bacteria | Firmicutes | Clostridia | Clostridiales | Peptostreptococcaceae | sf_5 | 2721 | AB088971.1 | termite gut homogenate clone Rs-N71 bacterium |
| Bacteria | Firmicutes | Clostridia | Clostridiales | Peptostreptococcaceae | sf_5 | 2913 | AB088960.2 | termite gut homogenate clone Rs-N82 bacterium |
| Bacteria | Firmicutes | Bacilli | Bacillales | Bacillaceae | sf_1 | 3345 | AY548955.1 | Bacillus pumilus str. S9 |
| Bacteria | Firmicutes | Clostridia | Clostridiales | Clostridiaceae | sf_12 | 4156 | AF407388.1 | MCB-contaminated groundwater-treating reactor clone RA9C1 |
| Bacteria | Acidobacteria | Acidobacteria-4 | Ellin6075/11-25 | Unclassified | sf_1 | 87 | AF097766.1 | activated sludge clone 2951 |
| Bacteria | Proteobacteria | Gammaproteobacteria | Enterobacteriales | Enterobacteriaceae | sf_1 | 8642 | AF373202.1 | Erwinia chrysanthemi str. 580 |
| Bacteria | Proteobacteria | Deltaproteobacteria | Syntrophobacterales | Syntrophobacteraceae | sf_1 | 10013 | AF420341.2 | hydrothermal sediment clone AF420341 |
| Bacteria | Unclassified | Unclassified | Unclassified | Unclassified | sf_92 | 9999 | AB089068.1 | termite gut homogenate clone Rs-J96 |
| Bacteria | Gemmatimonadetes | Unclassified | Unclassified | Unclassified | sf_5 | 1127 | AJ519396.1 | uranium mining waste pile near Johanngeorgenstadt soil clone JG37-AG-21 |
| Bacteria | Actinobacteria | Actinobacteria | Actinomycetales | Microbacteriaceae | sf_1 | 1197 | AF468440.1 | Arctic sea ice ARK10173 |
| Bacteria | Actinobacteria | Actinobacteria | Actinomycetales | Kineosporiaceae | sf_1 | 1598 | AY250880.1 | lichen-dominated Antarctic cryptoendolithic community clone FBP402 |
| Bacteria | Firmicutes | Clostridia | Clostridiales | Lachnospiraceae | sf_5 | 3066 | AF067965.1 | Clostridium methoxybenzovorans str. SR3; DSM 12182 |
| Bacteria | Firmicutes | Bacilli | Bacillales | Bacillaceae | sf_1 | 3661 | AB094471.1 | Bacillus sp. str. 2216.25.2 |
| Bacteria | Firmicutes | Clostridia | Clostridiales | Clostridiaceae | sf_12 | 4229 |  | |
| Bacteria | Bacteroidetes | Bacteroidetes | Bacteroidales | Prevotellaceae | sf_1 | 5249 | AY323524.1 | Prevotella denticola str. ATCC 35308 |
| Bacteria | Bacteroidetes | Sphingobacteria | Sphingobacteriales | Flexibacteraceae | sf_19 | 5667 | AY218749.1 | penguin droppings sediments clone KD6-118 |
| Bacteria | Chlorobi | Unclassified | Unclassified | Unclassified | sf_6 | 5294 | AY221073.1 | Mammoth cave clone CCM9b |
| Bacteria | Chloroflexi | Anaerolineae | Chloroflexi-1a | Unclassified | sf_1 | 583 | AJ278171.2 | anaerobic bioreactor clone SHD-238 |
| Bacteria | Cyanobacteria | Unclassified | Unclassified | Unclassified | sf_5 | 5030 | AY034793.1 | Hapalosiphon welwitschii |
| Bacteria | Spirochaetes | Spirochaetes | Spirochaetales | Spirochaetaceae | sf_1 | 6459 | AJ431240.1 | Spirochaeta sp. str. BHI80-158 |
| Bacteria | Proteobacteria | Alphaproteobacteria | Rhodobacterales | Rhodobacteraceae | sf_1 | 7263 |  | |
| Bacteria | Proteobacteria | Betaproteobacteria | Neisseriales | Neisseriaceae | sf_1 | 7675 | AY157705.1 | Neisseria sp. str. CCUG 46910 |
| Bacteria | Proteobacteria | Betaproteobacteria | Rhodocyclales | Rhodocyclaceae | sf_1 | 7956 |  | |
| Bacteria | Proteobacteria | Betaproteobacteria | Rhodocyclales | Rhodocyclaceae | sf_1 | 7907 | AJ315680.1 | Thauera aromatica str. LG356 |
| Bacteria | Proteobacteria | Betaproteobacteria | Burkholderiales | Burkholderiaceae | sf_1 | 7771 | AB021374.1 | Burkholderia glathei str. ATCC 29195T |
| Bacteria | Proteobacteria | Gammaproteobacteria | Xanthomonadales | Xanthomonadaceae | sf_3 | 8577 | AF442743.1 | Xanthomonas axonopodis pv. citri str. MA |
| Bacteria | Proteobacteria | Gammaproteobacteria | Unclassified | Unclassified | sf_3 | 8606 |  | |
| Bacteria | Proteobacteria | Gammaproteobacteria | Alteromonadales | Alteromonadaceae | sf_1 | 8533 |  | |
| Bacteria | Proteobacteria | Gammaproteobacteria | Alteromonadales | Alteromonadaceae | sf_1 | 9562 | AF513941.2 | Alteromonadaceae clone PH-B55N |
| Bacteria | Proteobacteria | Deltaproteobacteria | Myxococcales | Polyangiaceae | sf_3 | 10298 | AY193162.1 | marine tidal mat clone BTM36 |
| Bacteria | Actinobacteria | Actinobacteria | Actinomycetales | Unclassified | sf_3 | 2045 | AF507852.1 | hypersaline lake clone ML602J-44 |
| Bacteria | Actinobacteria | Actinobacteria | Actinomycetales | Nocardiaceae | sf_1 | 1940 | AB178563.1 | Rhodococcus opacus str. B-4 |
| Bacteria | Actinobacteria | Actinobacteria | Actinomycetales | Nocardiaceae | sf_1 | 1834 | AF430047.1 | Nocardia transvalensis str. DSM 43405 |
| Bacteria | Proteobacteria | Unclassified | Unclassified | Unclassified | sf_20 | 2520 |  | |
| Bacteria | Firmicutes | Clostridia | Clostridiales | Lachnospiraceae | sf_5 | 2937 | AF371541.1 | swine intestine clone p-2482-18B5 |
| Bacteria | Firmicutes | Clostridia | Clostridiales | Clostridiaceae | sf_12 | 4306 | AY261814.1 | UASB reactor granular sludge clone PD-UASB-4 bacterium |
| Bacteria | Chloroflexi | Unclassified | Unclassified | Unclassified | sf_7 | 757 | AJ306741.2 | DCP-dechlorinating consortium clone SHA-8 |
| Bacteria | Cyanobacteria | Cyanobacteria | Chloroplasts | Chloroplasts | sf_5 | 5183 | M37430.1 | Pisum sativum -- chloroplast |
| Bacteria | Spirochaetes | Spirochaetes | Spirochaetales | Spirochaetaceae | sf_1 | 6479 | Y08893.1 | Treponema sp |
| Bacteria | Spirochaetes | Spirochaetes | Spirochaetales | Spirochaetaceae | sf_1 | 6580 | AF056341.1 | Treponema sp. str. III:C:BA213 |
| Bacteria | Proteobacteria | Gammaproteobacteria | Alteromonadales | Alteromonadaceae | sf_1 | 9288 | AJ295715.1 | Alteromonas stellipolaris str. LMG 21861 |
| Bacteria | Proteobacteria | Gammaproteobacteria | Alteromonadales | Alteromonadaceae | sf_1 | 9143 | AJ417594.1 | Pseudoalteromonas agarivorans str. KMM 255 |
| Bacteria | Firmicutes | Bacilli | Bacillales | Bacillaceae | sf_1 | 3550 | AF142677.4 | Bacillus megaterium str. QM B1551 |
| Bacteria | Firmicutes | Bacilli | Bacillales | Bacillaceae | sf_1 | 3489 | AY167818.1 | Bacillus silvestris str. SAFN-010 |
| Bacteria | Firmicutes | Bacilli | Lactobacillales | Streptococcaceae | sf_1 | 3907 | U87830.1 | aortic heart valve patient with endocarditis clone v6 |
| Bacteria | Firmicutes | Clostridia | Clostridiales | Lachnospiraceae | sf_5 | 4212 | AB100488.1 | termite gut clone Rs-061 |
| Bacteria | Bacteroidetes | Bacteroidetes | Bacteroidales | Unclassified | sf_15 | 6069 | AJ289174.1 | corneal ulcer clone E1-K9 |
| Bacteria | Bacteroidetes | Bacteroidetes | Bacteroidales | Prevotellaceae | sf_1 | 6259 |  | |
| Bacteria | Bacteroidetes | Flavobacteria | Flavobacteriales | Flavobacteriaceae | sf_1 | 5915 | AJ534682.1 | ground water deep-well injection disposal site radioactive wastes Tomsk-7 clone S15A-MN27 bacterium |
| Bacteria | Proteobacteria | Alphaproteobacteria | Bradyrhizobiales | Hyphomicrobiaceae | sf_1 | 7646 | Y14304.1 | Hyphomicrobium aestuarii str. DSM 1564 |
| Bacteria | Proteobacteria | Alphaproteobacteria | Consistiales | Unclassified | sf_4 | 7105 | AB186982.1 | Mariana trough hydrothermal vent water 0.2micro-m filterable fraction clone MT-NB25 |
| Bacteria | Proteobacteria | Betaproteobacteria | Neisseriales | Unclassified | sf_1 | 8037 | AY323827.2 | Chitinimonas taiwanensis str. cf |
| Bacteria | Proteobacteria | Gammaproteobacteria | Pseudomonadales | Moraxellaceae | sf_3 | 9466 | AF509830.1 | Acinetobacter tandoii str. 4N13 |
| Bacteria | Proteobacteria | Deltaproteobacteria | Desulfovibrionales | Desulfovibrionaceae | sf_1 | 9826 | AB089108.2 | termite gut homogenate clone Rs-M72 proteobacterium |
| Bacteria | Proteobacteria | Deltaproteobacteria | Unclassified | Unclassified | sf_9 | 9760 | AY093467.1 | deep marine sediment clone MB-A2-137 |
| Bacteria | Proteobacteria | Deltaproteobacteria | AMD clone group | Unclassified | sf_1 | 10084 | AF543496.1 | acid mine drainage clone AS6 |
| Bacteria | Proteobacteria | Epsilonproteobacteria | Campylobacterales | Campylobacteraceae | sf_3 | 10484 | U03022.1 | Campylobacter helveticus |
| Bacteria | Actinobacteria | Actinobacteria | Actinomycetales | Sporichthyaceae | sf_1 | 1695 | AY250883.1 | lichen-dominated Antarctic cryptoendolithic community clone FBP417 |
| Bacteria | Firmicutes | Clostridia | Clostridiales | Lachnospiraceae | sf_5 | 3089 | AY169419.1 | Ruminococcus obeum |
| Bacteria | Firmicutes | Clostridia | Clostridiales | Clostridiaceae | sf_12 | 2915 | AY158079.1 | Tepidibacter thalassicus str. SC 562 |
| Bacteria | Firmicutes | Bacilli | Bacillales | Bacillaceae | sf_1 | 3918 | AF549498.1 | Bacillus subtilis |
| Bacteria | Firmicutes | Clostridia | Clostridiales | Lachnospiraceae | sf_5 | 4273 | AB089044.1 | termite gut homogenate clone Rs-M14 bacterium |
| Bacteria | Firmicutes | Clostridia | Clostridiales | Unclassified | sf_17 | 4307 |  | |
| Bacteria | Deinococcus-Thermus | Unclassified | Unclassified | Unclassified | sf_1 | 563 | AJ507298.1 | Vulcanithermus mediatlanticus str. TR |
| Bacteria | Verrucomicrobia | Unclassified | Unclassified | Unclassified | sf_3 | 40 | AJ401121.1 | Elbe river clone DEV055 |
| Bacteria | Bacteroidetes | Bacteroidetes | Bacteroidales | Prevotellaceae | sf_1 | 5398 | AY699286.1 | Prevotella ruminicola L16 |
| Bacteria | Bacteroidetes | Sphingobacteria | Sphingobacteriales | Crenotrichaceae | sf_11 | 6123 | AB078055.1 | Flexibacter japonensis str. IFO 16041 |
| Bacteria | Proteobacteria | Betaproteobacteria | Burkholderiales | Alcaligenaceae | sf_1 | 7902 | AF155147.1 | Alcaligenes faecalis str. M3A |
| Bacteria | Proteobacteria | Betaproteobacteria | Burkholderiales | Ralstoniaceae | sf_1 | 7761 | AF280433.1 | Ralstonia detusculanense str. APF11 |
| Bacteria | Proteobacteria | Gammaproteobacteria | Alteromonadales | Alteromonadaceae | sf_1 | 8863 | AF529060.1 | Alteromonas marina str. SW-47 |
| Bacteria | Proteobacteria | Gammaproteobacteria | Alteromonadales | Alteromonadaceae | sf_1 | 9640 | AY028205.1 | exposed to diatom detritus isolate str. Tw-10 Tw-10 |
| Bacteria | Firmicutes | Clostridia | Clostridiales | Syntrophomonadaceae | sf_5 | 2456 | AF482440.1 | granular sludge clone R4b14 |
| Bacteria | Firmicutes | Bacilli | Bacillales | Bacillaceae | sf_1 | 385 | X57309.1 | Geobacillus stearothermophilus str. T10 |
| Bacteria | Firmicutes | Catabacter | Unclassified | Unclassified | sf_4 | 4526 | AY133082.1 | TCE-contaminated site clone ccslm210 |
| Bacteria | Acidobacteria | Acidobacteria | Acidobacteriales | Acidobacteriaceae | sf_14 | 6335 | AF523984.1 | forested wetland clone FW45 |
| Bacteria | Spirochaetes | Spirochaetes | Spirochaetales | Spirochaetaceae | sf_1 | 6488 | AF093251.1 | Treponema primitia str. ZAS-1 |
| Bacteria | Proteobacteria | Alphaproteobacteria | Rhodobacterales | Rhodobacteraceae | sf_1 | 6980 | AJ582226.1 | Loktanella vestfoldensis str. LMG 22003 |
| Bacteria | Proteobacteria | Gammaproteobacteria | Alteromonadales | Alteromonadaceae | sf_1 | 8174 | AF114499.1 | attached marine recovered surface clone 17 proteobacterium |
| Bacteria | Proteobacteria | Gammaproteobacteria | Alteromonadales | Alteromonadaceae | sf_1 | 8580 | AJ295713.1 | Arctic seawater isolate str. R7076 |
| Bacteria | Actinobacteria | Actinobacteria | Acidimicrobiales | Unclassified | sf_1 | 1217 | AJ306762.2 | DCP-dechlorinating consortium clone SHA-34 |
| Bacteria | Actinobacteria | Actinobacteria | Actinomycetales | Micrococcaceae | sf_1 | 1686 | AY228479.1 | Yania halotolerans str. YIM 70085 |
| Bacteria | Actinobacteria | Actinobacteria | Actinomycetales | Micrococcaceae | sf_1 | 1582 | AY053487.1 | gas hydrate clone AT425_EubD11 |
| Bacteria | Actinobacteria | Actinobacteria | Actinomycetales | Micrococcaceae | sf_1 | 1573 | AJ315492.1 | Arthrobacter nicotianae str. SB42 |
| Bacteria | Firmicutes | Clostridia | Clostridiales | Lachnospiraceae | sf_5 | 3017 | AB088993.1 | termite gut homogenate clone Rs-D48 bacterium |
| Bacteria | Firmicutes | Bacilli | Bacillales | Staphylococcaceae | sf_1 | 3592 | Y12593.1 | Staphylococcus caprae str. DSM 20608 |
| Bacteria | Firmicutes | Bacilli | Bacillales | Staphylococcaceae | sf_1 | 3494 | AB079788.1 | Micrococcus luteus B-P 26 |
| Bacteria | Firmicutes | Clostridia | Clostridiales | Lachnospiraceae | sf_5 | 4525 | AB088991.1 | termite gut homogenate clone Rs-Q18 bacterium |
| Bacteria | Acidobacteria | Acidobacteria | Acidobacteriales | Acidobacteriaceae | sf_14 | 6364 | AY234728.1 | Solibacter usitatus Ellin6076 |
| Bacteria | Proteobacteria | Alphaproteobacteria | Ellin314/wr0007 | Unclassified | sf_1 | 7123 | AJ518774.1 | uranium mining waste pile near Johanngeorgenstadt soil clone JG37-AG-102 |
| Bacteria | Proteobacteria | Gammaproteobacteria | Oceanospirillales | Unclassified | sf_3 | 8327 | AF468398.1 | Arctic sea ice ARK10148 |
| Bacteria | Proteobacteria | Gammaproteobacteria | Aeromonadales | Aeromonadaceae | sf_1 | 9294 | AJ557849.1 | Arctic deep sea Isolation common chemoorganotrophic oxygen-respiring polar current d 1210 (50 m above sediment Hakon Mosby Mud Vulcano (HMMV 72N 14E)) via serial dilution series applying 2 g/l yeast or meat extract artifical seawater |
| Bacteria | Proteobacteria | Gammaproteobacteria | Alteromonadales | Alteromonadaceae | sf_1 | 8932 | AF045560.1 | Pseudoalteromonas antarctica str. N-1 |
| Bacteria | Fusobacteria | Fusobacteria | Fusobacterales | Fusobacteriaceae | sf_1 | 488 | NZ_AABF02000111.1 | Fusobacterium nucleatum subsp. vincentii str. ATCC 49256 |
| Bacteria | Firmicutes | Clostridia | Clostridiales | Peptostreptococcaceae | sf_5 | 3112 | CR933145.1 | Evry municipal wastewater treatment plant clone 012C11_B_SD_P15 |
| Bacteria | Firmicutes | Bacilli | Bacillales | Bacillaceae | sf_1 | 234 | AJ293805.1 | Bacillus vulcani str. 3S-1 |
| Bacteria | Firmicutes | Bacilli | Bacillales | Bacillaceae | sf_1 | 3540 | AB034836.1 | Geobacillus thermoleovorans str. B23 |
| Bacteria | Cyanobacteria | Cyanobacteria | Chloroplasts | Chloroplasts | sf_5 | 5006 |  | |
| Bacteria | Cyanobacteria | Cyanobacteria | Chloroplasts | Chloroplasts | sf_5 | 5182 | M81884.1 | Epifagus virginiana -- chloroplast |
| Bacteria | Proteobacteria | Alphaproteobacteria | Caulobacterales | Caulobacteraceae | sf_1 | 6968 | AB021415.1 | Brevundimonas diminuta str. IAM 12691T |
| Bacteria | Proteobacteria | Deltaproteobacteria | AMD clone group | Unclassified | sf_1 | 6830 | AF523882.1 | coal effluent wetland clone RCP124 |
| Bacteria | Proteobacteria | Gammaproteobacteria | Unclassified | Unclassified | sf_3 | 9568 | AF523888.1 | forested wetland clone RCP2-96 |
| Bacteria | Proteobacteria | Gammaproteobacteria | Alteromonadales | Alteromonadaceae | sf_1 | 8578 | AY147906.1 | Marinobacter lipolyticus str. SM-19 |
| Bacteria | Proteobacteria | Gammaproteobacteria | Pasteurellales | Pasteurellaceae | sf_1 | 9360 | AF224297.1 | Pasteurella multocida subsp. gallicida str. MCCM 00021 subsp. |
| Bacteria | Proteobacteria | Deltaproteobacteria | Myxococcales | Unclassified | sf_1 | 10092 | AY102330.1 | heavy metal-contaminated soil clone a13134 |
| Bacteria | Proteobacteria | Deltaproteobacteria | Myxococcales | Polyangiaceae | sf_3 | 9874 | AJ532714.1 | uranium mining waste pile clone JG34-KF-243 proteobacterium |
| Bacteria | Proteobacteria | Deltaproteobacteria | AMD clone group | Unclassified | sf_1 | 9945 | AF225446.1 | acid mine drainage clone BA18 |
| Bacteria | Proteobacteria | Epsilonproteobacteria | Campylobacterales | Helicobacteraceae | sf_3 | 10438 | AF154101.1 | hydrocarbon seep clone GCA014 |
| Bacteria | Actinobacteria | Actinobacteria | Actinomycetales | Corynebacteriaceae | sf_1 | 1428 | AF537604.1 | Corynebacterium simulans National Microbiology Laboratory Special identifier 00-0186 |
| Bacteria | Firmicutes | Desulfotomaculum | Unclassified | Unclassified | sf_1 | 2359 | AB091324.1 | UASB granular sludge clone JP |
| Bacteria | Proteobacteria | Deltaproteobacteria | Unclassified | Unclassified | sf_9 | 244 | AY093483.1 | deep marine sediment clone MB-C2-152 |
| Bacteria | Firmicutes | Bacilli | Bacillales | Thermoactinomycetaceae | sf_1 | 3301 | AB088361.1 | Thermoactinomyces sp. str. 700375 |
| Bacteria | Firmicutes | Clostridia | Clostridiales | Lachnospiraceae | sf_5 | 4535 | AF376201.1 | ckncm297-B1-1 clone |
| Bacteria | Unclassified | Unclassified | Unclassified | Unclassified | sf_160 | 485 | AY193179.1 | thermal spring mat clone O1aA90 |
| Bacteria | Bacteroidetes | Flavobacteria | Flavobacteriales | Flavobacteriaceae | sf_1 | 6274 |  | |
| Bacteria | Bacteroidetes | Bacteroidetes | Bacteroidales | Unclassified | sf_15 | 6324 | AY216445.1 | temperate estuarine mud clone KM02 |
| Bacteria | Proteobacteria | Betaproteobacteria | Rhodocyclales | Rhodocyclaceae | sf_1 | 7800 | AY013692.1 | sample taken upstream landfill clone BVC77 landfill |
| Bacteria | Proteobacteria | Betaproteobacteria | Methylophilales | Methylophilaceae | sf_1 | 8137 | AF289159.1 | freshwater clone PRD01a011B |
| Bacteria | Proteobacteria | Gammaproteobacteria | aquatic clone group | Unclassified | sf_1 | 8957 | AF354614.1 | marine clone Arctic97C-5 |
| Bacteria | Proteobacteria | Gammaproteobacteria | Pseudomonadales | Pseudomonadaceae | sf_1 | 9613 | U01916.1 | Pseudomonas flavescens str. B62 |
| Bacteria | Proteobacteria | Gammaproteobacteria | Alteromonadales | Alteromonadaceae | sf_1 | 8753 | AY553079.1 | Idiomarina loihiensis str. GSP37 |
| Bacteria | Proteobacteria | Gammaproteobacteria | Alteromonadales | Alteromonadaceae | sf_1 | 9058 | X82136.1 | Pseudoalteromonas carrageenovora str. ATCC 12662T |
| Bacteria | Proteobacteria | Gammaproteobacteria | Alteromonadales | Alteromonadaceae | sf_1 | 9218 | X67024.1 | Pseudoalteromonas haloplanktis str. ATCC 14393 |
| Bacteria | Proteobacteria | Deltaproteobacteria | Desulfobacterales | Unclassified | sf_4 | 9951 | AF523973.1 | forested wetland clone FW13 |
| Bacteria | LD1PA group | Unclassified | Unclassified | Unclassified | sf_1 | 10118 | AY114324.1 | anoxic marine sediment clone LD1-PA38 |
| Bacteria | Proteobacteria | Deltaproteobacteria | Desulfobacterales | Desulfobulbaceae | sf_1 | 9739 | AJ535252.1 | gas hydrate clone Hyd89-51 |
| Bacteria | Actinobacteria | Actinobacteria | Actinomycetales | Unclassified | sf_3 | 1577 | AB089072.1 | termite gut homogenate clone Rs-N91 bacterium |
| Bacteria | Actinobacteria | Actinobacteria | Actinomycetales | Micrococcaceae | sf_1 | 2020 | AF543278.1 | Rothia dentocariosa str. ChDC B200 |
| Bacteria | Actinobacteria | Actinobacteria | Actinomycetales | Actinomycetaceae | sf_1 | 1227 | AB062278.1 | Actinomyces naeslundii |
| Bacteria | Actinobacteria | Actinobacteria | Actinomycetales | Pseudonocardiaceae | sf_1 | 1863 |  | |
| Bacteria | Firmicutes | Clostridia | Clostridiales | Unclassified | sf_17 | 926 |  | |
| Bacteria | Firmicutes | Clostridia | Clostridiales | Lachnospiraceae | sf_5 | 2681 | AB088974.1 | termite gut homogenate clone Rs-K41 bacterium |
| Bacteria | Proteobacteria | Deltaproteobacteria | AMD clone group | Unclassified | sf_1 | 3084 | AF523884.1 | coal effluent wetland clone RCP216 |
| Bacteria | Firmicutes | Clostridia | Clostridiales | Lachnospiraceae | sf_5 | 4315 | AB089040.1 | termite gut homogenate clone Rs-N94 bacterium |
| Bacteria | Verrucomicrobia | Verrucomicrobiae | Verrucomicrobiales | Verrucomicrobia subdivision 5 | sf_1 | 885 | AJ390455.1 | soil clone PBS-III-26 |
| Bacteria | Gemmatimonadetes | Unclassified | Unclassified | Unclassified | sf_5 | 227 | AJ519397.1 | uranium mining waste pile clone JG37-AG-36 |
| Bacteria | Cyanobacteria | Cyanobacteria | Chloroplasts | Chloroplasts | sf_5 | 5040 | Y18934.1 | Solanum nigrum |
| Bacteria | Proteobacteria | Betaproteobacteria | Burkholderiales | Alcaligenaceae | sf_1 | 7838 | AB195161.1 | Alcaligenes defragrans str. PD-19 |
| Bacteria | Proteobacteria | Betaproteobacteria | Burkholderiales | Ralstoniaceae | sf_1 | 8128 | M32021.1 | Cupriavidus necator |
| Bacteria | Proteobacteria | Deltaproteobacteria | Desulfovibrionales | Desulfomicrobiaceae | sf_1 | 10079 | AJ277896.1 | Desulfomicrobium baculatum str. DSM 1742 |
| Bacteria | Proteobacteria | Deltaproteobacteria | Unclassified | Unclassified | sf_9 | 10049 | AJ306774.1 | DCP-dechlorinating consortium clone SHA-72 |
| Bacteria | Proteobacteria | Epsilonproteobacteria | Campylobacterales | Helicobacteraceae | sf_3 | 10430 | AF506783.1 | Helicobacter heilmannii str. MM2 |
| Bacteria | Firmicutes | Bacilli | Bacillales | Bacillaceae | sf_1 | 571 | Z26922.1 | Bacillus caldotenax str. DSM 406 |
| Bacteria | Firmicutes | Bacilli | Bacillales | Staphylococcaceae | sf_1 | 3432 | AY188939.1 | deep-sea sediment isolate str. P_wp0225 |
| Bacteria | Firmicutes | Bacilli | Lactobacillales | Lactobacillaceae | sf_1 | 3330 | AB107637.1 | Lactobacillus kitasatonis str. KM9212 |
| Bacteria | Firmicutes | Bacilli | Lactobacillales | Aerococcaceae | sf_1 | 3833 | L08623.1 | Carnobacterium alterfunditum |
| Bacteria | Synergistes | Unclassified | Unclassified | Unclassified | sf_3 | 117 | AB089065.2 | termite gut homogenate clone Rs-D89 |
| Bacteria | Bacteroidetes | Bacteroidetes | Bacteroidales | Unclassified | sf_15 | 5475 | AJ249101.1 | SHA-25 clone |
| Bacteria | Cyanobacteria | Cyanobacteria | Chloroplasts | Chloroplasts | sf_5 | 4976 | AF244550.1 | Calypogeia muelleriana |
| Bacteria | Proteobacteria | Alphaproteobacteria | Unclassified | Unclassified | sf_2 | 7188 | AB089085.1 | termite gut homogenate clone Rs-B50 proteobacterium |
| Bacteria | Gemmatimonadetes | Unclassified | Unclassified | Unclassified | sf_5 | 9464 | AF432648.1 | lodgepole pine rhizosphere soil British Columbia Ministry Forests Long-Term Soil Productivity |
| Bacteria | Proteobacteria | Deltaproteobacteria | Desulfovibrionales | Desulfovibrionaceae | sf_1 | 10262 | AF228127.2 | Desulfovibrio sp. str. Ac5.2 |
| Bacteria | Acidobacteria | Unclassified | Unclassified | Unclassified | sf_1 | 572 | AF523986.1 | forested wetland clone FW144 |
| Bacteria | Cyanobacteria | Cyanobacteria | Chloroplasts | Chloroplasts | sf_5 | 4966 | AF244549.1 | Adiantum pedatum |
| Bacteria | BRC1 | Unclassified | Unclassified | Unclassified | sf_1 | 5143 | AJ390438.1 | soil clone PBS-II-1 |
| Bacteria | Proteobacteria | Gammaproteobacteria | Legionellales | Coxiellaceae | sf_3 | 8969 | AJ536861.1 | uranium mining waste pile soil sample clone JG30-KF-C15 proteobacterium |
| Bacteria | Proteobacteria | Gammaproteobacteria | Pseudomonadales | Pseudomonadaceae | sf_1 | 8209 | AJ518787.1 | uranium mining waste pile clone JG37-AG-122 proteobacterium |
| Bacteria | Firmicutes | Clostridia | Unclassified | Unclassified | sf_4 | 2398 | AY093477.1 | deep marine sediment clone MB-C2-106 |
| Bacteria | Firmicutes | Clostridia | Clostridiales | Lachnospiraceae | sf_5 | 2804 | AY353957.1 | Clostridium amygdalinum str. BR-10 |
| Bacteria | Firmicutes | Clostridia | Clostridiales | Lachnospiraceae | sf_5 | 3171 | AY169414.1 | Lachnospira pectinoschiza |
| Bacteria | Firmicutes | Bacilli | Bacillales | Bacillaceae | sf_1 | 3460 | AY312404.1 | Geobacillus jurassicus str. DS1 |
| Bacteria | Firmicutes | Clostridia | Clostridiales | Clostridiaceae | sf_12 | 4310 | AB100478.1 | termite gut clone Rs-056 |
| Bacteria | Firmicutes | Clostridia | Clostridiales | Clostridiaceae | sf_12 | 4507 | AB088984.1 | termite gut homogenate clone Rs-N21 bacterium |
| Bacteria | Unclassified | Unclassified | Unclassified | Unclassified | sf_160 | 4410 |  | |
| Bacteria | Bacteroidetes | Bacteroidetes | Bacteroidales | Porphyromonadaceae | sf_1 | 5817 | AB088927.2 | termite gut homogenate clone Rs-N56 bacterium |
| Bacteria | Bacteroidetes | Bacteroidetes | Bacteroidales | Porphyromonadaceae | sf_1 | 5961 | AJ488070.1 | chlorobenzene-degrading consortium clone IA-16 |
| Bacteria | Bacteroidetes | Sphingobacteria | Sphingobacteriales | Unclassified | sf_3 | 6168 | AF534435.1 | Toolik Lake main station at 3 m depth clone TLM11/TLMdgge04 |
| Bacteria | Bacteroidetes | Sphingobacteria | Sphingobacteriales | Crenotrichaceae | sf_11 | 6267 | AF316115.1 | Cilia- respiratory isolate str. 243-54 |
| Bacteria | Chlorobi | Chlorobia | Chlorobiales | Chlorobiaceae | sf_1 | 859 | Y08105.1 | Chlorobium phaeovibrioides str. 2631 |
| Bacteria | Proteobacteria | Alphaproteobacteria | Rickettsiales | Unclassified | sf_2 | 6639 |  | |
| Bacteria | Proteobacteria | Gammaproteobacteria | Thiotrichales | Thiotrichaceae | sf_3 | 9321 | AB108786.1 | marine sediment clone Tokyo Bay D |
| Bacteria | Natronoanaerobium | Unclassified | Unclassified | Unclassified | sf_1 | 3570 | AF454298.1 | Bacillus sp. clone ML1228J-1 |
| Bacteria | Firmicutes | Bacilli | Bacillales | Staphylococcaceae | sf_1 | 3654 | AF322002.1 | Staphylococcus pettenkoferi str. B3117 |
| Bacteria | Bacteroidetes | Flavobacteria | Flavobacteriales | Flavobacteriaceae | sf_1 | 5317 | AB078057.1 | Tenacibaculum maritimum str. IFO 15946 |
| Bacteria | Cyanobacteria | Cyanobacteria | Chloroplasts | Chloroplasts | sf_11 | 5098 | AF289245.1 | Euglena tripteris str. UW OB |
| Bacteria | Proteobacteria | Betaproteobacteria | Rhodocyclales | Rhodocyclaceae | sf_1 | 7824 | AB089101.1 | termite gut homogenate clone Rs-B77 proteobacterium |
| Bacteria | Proteobacteria | Gammaproteobacteria | uranium waste clones | Unclassified | sf_1 | 8747 | AJ536882.1 | uranium waste soil clone JG30-KF-CM35 |
| Bacteria | Proteobacteria | Gammaproteobacteria | Legionellales | Coxiellaceae | sf_3 | 8457 | AJ240918.1 | 5' clone CHAB-XI-27 |
| Bacteria | Firmicutes | Clostridia | Clostridiales | Lachnospiraceae | sf_5 | 3111 | AB034059.1 | rumen clone 6C3d-11 |
| Bacteria | Firmicutes | Bacilli | Bacillales | Bacillaceae | sf_1 | 1050 | AJ717384.1 | Bacillus firmus CV93b |
| Bacteria | Firmicutes | Bacilli | Bacillales | Staphylococcaceae | sf_1 | 3794 |  | |
| Bacteria | OP9/JS1 | OP9 | Unclassified | Unclassified | sf_1 | 726 | AF027086.1 | hot spring clone OPB72 |
| Bacteria | Bacteroidetes | Flavobacteria | Flavobacteriales | Flavobacteriaceae | sf_1 | 5991 | AB032506.1 | Tenacibaculum ovolyticum str. IAM14318 |
| Bacteria | Acidobacteria | Acidobacteria | Acidobacteriales | Acidobacteriaceae | sf_14 | 6368 | AF200698.1 | soil clone UA2 |
| Bacteria | Proteobacteria | Gammaproteobacteria | Pseudomonadales | Pseudomonadaceae | sf_1 | 8691 | AE004501.1 | Pseudomonas aeruginosa str. PAO1 |
| Bacteria | Proteobacteria | Gammaproteobacteria | Pseudomonadales | Pseudomonadaceae | sf_1 | 9366 | AJ293826.1 | Arctic seawater isolate str. R7366 |
| Bacteria | Chloroflexi | Dehalococcoidetes | Unclassified | Unclassified | sf_1 | 2339 | AJ519643.1 | uranium mill tailings soil sample clone Sh765B-TzT-20 bacterium |
| Bacteria | Firmicutes | Clostridia | Clostridiales | Peptococc/Acidaminococc | sf_11 | 428 | AJ488090.1 | chlorobenzene-degrading consortium clone IIIA-1 |
| Bacteria | Firmicutes | Clostridia | Clostridiales | Peptostreptococcaceae | sf_5 | 3182 | AB088970.1 | termite gut homogenate clone Rs-Q64 bacterium |
| Bacteria | Firmicutes | Bacilli | Bacillales | Staphylococcaceae | sf_1 | 3545 |  | |
| Bacteria | Firmicutes | Bacilli | Bacillales | Staphylococcaceae | sf_1 | 3822 | AJ320272.1 | Staphylococcus succinus str. SB72 |
| Bacteria | Firmicutes | Bacilli | Lactobacillales | Lactobacillaceae | sf_1 | 3418 | AB001836.2 | Lactobacillus subsp. aviarius |
| Bacteria | Firmicutes | Clostridia | Clostridiales | Clostridiaceae | sf_12 | 4157 | AB089043.1 | termite gut homogenate clone Rs-A15 bacterium |
| Bacteria | OP10 | Unclassified | Unclassified | Unclassified | sf_4 | 484 | AF524022.1 | forested wetland clone FW68 |
| Bacteria | Proteobacteria | Gammaproteobacteria | Legionellales | Legionellaceae | sf_1 | 8865 | AF468229.1 | Arctic pack ice; northern Fram Strait; 80 31.1 N; 01 deg 59.7 min E clone ARKCH2Br2-23 |
| Bacteria | Proteobacteria | Gammaproteobacteria | Oceanospirillales | Alcanivoraceae | sf_1 | 8335 | AB055205.1 | Alcanivorax sp. str. K3-3 (MBIC 4323) |
| Bacteria | Proteobacteria | Gammaproteobacteria | Alteromonadales | Alteromonadaceae | sf_1 | 9386 | AB016267.1 | Alteromonas sp. str. NIBH P2M11 |
| Bacteria | Proteobacteria | Gammaproteobacteria | Pasteurellales | Pasteurellaceae | sf_1 | 8228 | AF268964.1 | Actinobacillus indolicus str. H1419 |
| Bacteria | Proteobacteria | Gammaproteobacteria | Pasteurellales | Pasteurellaceae | sf_1 | 8861 | AB004031.1 | Haemophilus parasuis 427 |
| Bacteria | Proteobacteria | Gammaproteobacteria | Enterobacteriales | Enterobacteriaceae | sf_1 | 8504 | AF476104.1 | Dysmicoccus neobrevipes symbiont |
| Bacteria | Actinobacteria | Actinobacteria | Actinomycetales | Promicromonosporaceae | sf_1 | 1671 | X79453.1 | Cellulosimicrobium cellulans str. NCIMB 11025 |
| Bacteria | Actinobacteria | Actinobacteria | Actinomycetales | Micrococcaceae | sf_1 | 1324 | AF479325.1 | glacial ice isolate str. CanDirty1 |
| Bacteria | Firmicutes | Bacilli | Bacillales | Staphylococcaceae | sf_1 | 3638 | X86641.1 | Staphylococcus sp str. AG-30 |
| Bacteria | Firmicutes | Clostridia | Clostridiales | Clostridiaceae | sf_12 | 4477 | AB089042.1 | termite gut homogenate clone Rs-N85 bacterium |
| Bacteria | Synergistes | Unclassified | Unclassified | Unclassified | sf_3 | 60 | AF481216.1 | Flexistipes sp. str. E3_33 |
| Bacteria | Bacteroidetes | Bacteroidetes | Bacteroidales | Prevotellaceae | sf_1 | 5916 | AY244931.1 | cow rumen clone BE14 |
| Bacteria | Bacteroidetes | Bacteroidetes | Bacteroidales | Unclassified | sf_15 | 5481 | AJ535258.1 | marine sediment above hydrate ridge clone Hyd89-72 bacterium |
| Bacteria | Proteobacteria | Alphaproteobacteria | Bradyrhizobiales | Methylobacteriaceae | sf_1 | 7585 | U58018.1 | Methylobacterium thiocyanatum str. ALL/SCN-P |
| Bacteria | Proteobacteria | Betaproteobacteria | Burkholderiales | Comamonadaceae | sf_1 | 7965 | X72724.1 | Anoxobacterium dechloraticum |
| Bacteria | Proteobacteria | Gammaproteobacteria | Pseudomonadales | Pseudomonadaceae | sf_1 | 9068 | AF143245.1 | Pseudomonas stutzeri str. A1501 |
| Bacteria | Proteobacteria | Gammaproteobacteria | Enterobacteriales | Enterobacteriaceae | sf_1 | 8283 | AF263562.1 | Heteropsylla texana symbiont |
| Bacteria | Proteobacteria | Epsilonproteobacteria | Campylobacterales | Unclassified | sf_1 | 10543 | U15103.1 | hydrothermal vent clone PVB_10 |
| Bacteria | Actinobacteria | Actinobacteria | Unclassified | Unclassified | sf_1 | 1898 | AB089074.1 | termite gut homogenate clone Rs-J10 bacterium |
| Bacteria | Firmicutes | Bacilli | Bacillales | Bacillaceae | sf_1 | 3492 | AB042061.1 | Bacillus subtilis str. IAM 12118T |
| Bacteria | Firmicutes | Bacilli | Bacillales | Staphylococcaceae | sf_1 | 3569 | L37596.1 | Staphylococcus saprophyticus |
| Bacteria | Firmicutes | Clostridia | Clostridiales | Clostridiaceae | sf_12 | 4225 | AB100486.1 | termite gut clone Rs-116 |
| Bacteria | Firmicutes | Clostridia | Clostridiales | Clostridiaceae | sf_12 | 4266 | AB088951.1 | termite gut homogenate clone Rs-M86 bacterium |
| Bacteria | Unclassified | Unclassified | Unclassified | Unclassified | sf_93 | 925 | AF254404.1 | 4MB-degrading consortium clone UASB_TL26 |
| Bacteria | Bacteroidetes | Bacteroidetes | Bacteroidales | Porphyromonadaceae | sf_1 | 5680 | AB100460.1 | termite gut clone Rs-106 |
| Bacteria | Bacteroidetes | Bacteroidetes | Bacteroidales | Prevotellaceae | sf_1 | 5940 | AF481226.1 | Prevotella sp. str. E7_34 |
| Bacteria | Chlamydiae | Chlamydiae | Chlamydiales | Parachlamydiaceae | sf_1 | 4964 | AY082465.1 | neutral pH mine biofilm clone 44a-B1-34 |
| Bacteria | Proteobacteria | Betaproteobacteria | Burkholderiales | Oxalobacteraceae | sf_1 | 8032 | AB074523.1 | Aquaspirillum arcticum str. IAM 14963 |
| Bacteria | Proteobacteria | Gammaproteobacteria | Pseudomonadales | Pseudomonadaceae | sf_1 | 9310 | AJ519791.1 | Pseudomonas sp. str. AC-167 |
| Bacteria | Proteobacteria | Gammaproteobacteria | Alteromonadales | Alteromonadaceae | sf_1 | 9239 | AF468400.1 | Arctic sea ice ARK10228 |
| Bacteria | Proteobacteria | Deltaproteobacteria | Desulfovibrionales | Desulfovibrionaceae | sf_1 | 10071 | M34113.1 | Desulfovibrio desulfuricans |
| Bacteria | Actinobacteria | Actinobacteria | Actinomycetales | Micrococcaceae | sf_1 | 1266 | AF134179.1 | Arthrobacter psychrolactophilus |
| Bacteria | Natronoanaerobium | Unclassified | Unclassified | Unclassified | sf_1 | 2437 | AF507879.2 | Mono Lake at depth 23m station 6 July 2000 clone ML623J-19 |
| Bacteria | Firmicutes | Clostridia | Clostridiales | Lachnospiraceae | sf_5 | 2844 | AF202260.1 | Pseudobutyrivibrio ruminis str. pC-XS2 |
| Bacteria | Firmicutes | Clostridia | Clostridiales | Lachnospiraceae | sf_5 | 3038 | AF371648.1 | swine intestine clone p-1594-c5 |
| Bacteria | Firmicutes | Bacilli | Lactobacillales | Aerococcaceae | sf_1 | 3870 | AB022027.1 | Abiotrophia para-adiacens str. TKT1 |
| Bacteria | Firmicutes | Clostridia | Clostridiales | Clostridiaceae | sf_12 | 4297 |  | |
| Bacteria | Firmicutes | Clostridia | Clostridiales | Lachnospiraceae | sf_5 | 4512 | AF332711.1 | granular sludge clone UASB_brew_B25 |
| Bacteria | Caldithrix | Unclassified | Caldithrales | Caldithraceae | sf_2 | 91 | AF323775.1 | benzoate-degrading consortium clone BA059 |
| Bacteria | Cyanobacteria | Unclassified | Unclassified | Unclassified | sf_5 | 4998 |  | |
| Bacteria | Proteobacteria | Alphaproteobacteria | Azospirillales | Unclassified | sf_1 | 7400 | AF524861.1 | sphagnum peat bog clone K-5b5 |
| Bacteria | Proteobacteria | Gammaproteobacteria | Pseudomonadales | Moraxellaceae | sf_3 | 8727 | AY251390.1 | Alkanindiges hongkongensis str. HKU9 |
| Bacteria | Proteobacteria | Gammaproteobacteria | Pseudomonadales | Pseudomonadaceae | sf_1 | 9469 | AF290486.1 | cf. Pseudomonas sp. clone Llangefni 52 |
| Bacteria | Proteobacteria | Gammaproteobacteria | Pasteurellales | Pasteurellaceae | sf_1 | 8555 | AY613451.1 | Haemophilus influenzae str. M9741 |
| Bacteria | Proteobacteria | Gammaproteobacteria | Pasteurellales | Pasteurellaceae | sf_1 | 9628 | AF549390.1 | Histophilus somni str. CCUG 12839 |
| Bacteria | Proteobacteria | Deltaproteobacteria | Desulfobacterales | Desulfoarculaceae | sf_2 | 10227 | AY193132.1 | marine sediment clone Bol11 |
| Bacteria | Proteobacteria | Deltaproteobacteria | Desulfobacterales | Desulfobacteraceae | sf_5 | 9875 | AF420354.1 | hydrothermal sediment clone AF420354 |
| Bacteria | Actinobacteria | Actinobacteria | Actinomycetales | Nocardiopsaceae | sf_1 | 1385 | AF178988.1 | Streptomonospora salina str. YIM90002 |
| Bacteria | Actinobacteria | Actinobacteria | Actinomycetales | Cellulomonadaceae | sf_1 | 1586 | X83800.1 | Cellulomonas gelida str. DSM 20111T |
| Bacteria | Firmicutes | Clostridia | Clostridiales | Peptostreptococcaceae | sf_5 | 2729 | AJ306754.1 | DCP-dechlorinating consortium clone SHA-58 |
| Bacteria | Firmicutes | Bacilli | Bacillales | Bacillaceae | sf_1 | 3283 | AB021194.1 | Bacillus niacini str. IFO15566 |
| Bacteria | Firmicutes | Bacilli | Bacillales | Staphylococcaceae | sf_1 | 3605 |  | |
| Bacteria | Firmicutes | Clostridia | Clostridiales | Clostridiaceae | sf_12 | 4584 | X71852.1 | Clostridium papyrosolvens str. DSM 2782 |
| Bacteria | Firmicutes | Clostridia | Clostridiales | Clostridiaceae | sf_12 | 4554 | AB100479.1 | termite gut clone Rs-068 |
| Bacteria | Firmicutes | Clostridia | Clostridiales | Clostridiaceae | sf_12 | 4321 | AB089045.1 | termite gut homogenate clone Rs-C76 bacterium |
| Bacteria | Bacteroidetes | Bacteroidetes | Bacteroidales | Prevotellaceae | sf_1 | 5946 | AF385511.1 | tongue dorsa clone DO027 |
| Bacteria | Chlorobi | Unclassified | Unclassified | Unclassified | sf_8 | 636 | AY118152.1 | benzene-degrading nitrate-reducing consortium clone Cart-N3 bacterium |
| Bacteria | Cyanobacteria | Cyanobacteria | Prochlorales | Unclassified | sf_1 | 5001 | AJ347056.1 | sponge clone TK09 |
| Bacteria | Proteobacteria | Betaproteobacteria | Rhodocyclales | Rhodocyclaceae | sf_1 | 7762 | AF150698.1 | Elbe River snow isolate Iso18 Iso18_1411 |
| Bacteria | Proteobacteria | Gammaproteobacteria | Pseudomonadales | Pseudomonadaceae | sf_1 | 9240 | AJ278812.1 | Pseudomonas fluorescens str. CHA0 |
| Bacteria | Proteobacteria | Gammaproteobacteria | Pseudomonadales | Pseudomonadaceae | sf_1 | 8755 | AJ288146.1 | Pseudomonas sp. SK-1-3-1 |
| Bacteria | Proteobacteria | Epsilonproteobacteria | Campylobacterales | Unclassified | sf_1 | 10475 | AF420359.1 | hydrothermal sediment clone AF420359 |
| Bacteria | Firmicutes | Unclassified | Unclassified | Unclassified | sf_8 | 2433 | AF282252.1 | Ferribacter thermoautotrophicus str. JW/JH-Fiji-2 |
| Bacteria | Firmicutes | Bacilli | Bacillales | Bacillaceae | sf_1 | 462 | Z26927.1 | Geobacillus thermodenitrificans str. DSM 466 |
| Bacteria | Firmicutes | Bacilli | Lactobacillales | Lactobacillaceae | sf_1 | 3703 | AY389803.1 | Lactobacillus salivarius str. RA2115 |
| Bacteria | Firmicutes | Clostridia | Clostridiales | Lachnospiraceae | sf_5 | 4571 | X85022.1 | Faecalibacterium prausnitzii str. ATCC 27766 |
| Bacteria | Firmicutes | Clostridia | Clostridiales | Clostridiaceae | sf_12 | 4169 |  | |
| Bacteria | SPAM | Unclassified | Unclassified | Unclassified | sf_1 | 705 | AJ519639.1 | uranium tailings soil clone Sh765B-AG-45 |
| Bacteria | Proteobacteria | Gammaproteobacteria | Enterobacteriales | Enterobacteriaceae | sf_6 | 646 | AY695840.1 | Opitutus sp. str. SA-9 |
| Bacteria | Bacteroidetes | Bacteroidetes | Bacteroidales | Unclassified | sf_15 | 5544 | AY188304.1 | marine? clone KD3-17 |
| Bacteria | Bacteroidetes | Sphingobacteria | Sphingobacteriales | Flexibacteraceae | sf_19 | 6124 | AB078054.1 | Flexibacter flexilis subsp. pelliculosus str. IFO 16028 subsp. |
| Bacteria | Proteobacteria | Gammaproteobacteria | Thiotrichales | Thiotrichaceae | sf_3 | 8741 | AF532771.1 | marine sediment clone Limfjorden L10 |
| Bacteria | Proteobacteria | Gammaproteobacteria | Pseudomonadales | Pseudomonadaceae | sf_1 | 9056 | AB037546.1 | Pseudomonas aeruginosa str. #47 |
| Bacteria | Proteobacteria | Gammaproteobacteria | Pseudomonadales | Pseudomonadaceae | sf_1 | 9295 |  | |
| Bacteria | Actinobacteria | Actinobacteria | Actinomycetales | Cellulomonadaceae | sf_1 | 1748 | Y18378.1 | Beutenbergia cavernosa str. DSM 12333 |
| Bacteria | Firmicutes | Clostridia | Clostridiales | Lachnospiraceae | sf_5 | 2825 | AF105403.1 | Butyrivibrio fibrisolvens str. LP1265 |
| Bacteria | Firmicutes | gut clone group | Unclassified | Unclassified | sf_1 | 4298 | AY207065.1 | human mouth clone P4PA_66 |
| Bacteria | Firmicutes | Clostridia | Clostridiales | Lachnospiraceae | sf_5 | 4514 | AB088983.2 | termite gut homogenate clone Rs-B34 bacterium |
| Bacteria | Synergistes | Unclassified | Unclassified | Unclassified | sf_3 | 353 | AY261810.1 | UASB reactor granular sludge clone PD-UASB-13 G+C |
| Bacteria | Acidobacteria | Acidobacteria | Acidobacteriales | Acidobacteriaceae | sf_14 | 541 | AJ519667.1 | uranium mill tailings soil sample clone GuBH2-AG-47 sp. |
| Bacteria | Bacteroidetes | Bacteroidetes | Bacteroidales | Prevotellaceae | sf_1 | 6152 | AF001768.1 | rumen clone RF37 |
| Bacteria | Bacteroidetes | Flavobacteria | Flavobacteriales | Flavobacteriaceae | sf_1 | 5942 |  | |
| Bacteria | Proteobacteria | Gammaproteobacteria | Xanthomonadales | Xanthomonadaceae | sf_3 | 9270 | AJ293463.1 | Stenotrophomonas rhizophila str. e-p10 |
| Bacteria | Proteobacteria | Gammaproteobacteria | Aeromonadales | Succinivibrionaceae | sf_1 | 8822 | AF497809.1 | Anaerobiospirillum sp. str. 3J102 |
| Bacteria | Proteobacteria | Epsilonproteobacteria | Campylobacterales | Campylobacteraceae | sf_3 | 10464 | AY135395.1 | Campylobacter sp. str. NO2B |
| Bacteria | Actinobacteria | Actinobacteria | Unclassified | Unclassified | sf_1 | 1367 |  | |
| Bacteria | Actinobacteria | Actinobacteria | Actinomycetales | Microbacteriaceae | sf_1 | 1437 | AJ575522.1 | freshwater clone SV1-16 |
| Bacteria | Firmicutes | Bacilli | Bacillales | Bacillaceae | sf_1 | 829 | AY191842.2 | Geobacillus sp. str. YMTC1049 |
| Bacteria | Firmicutes | Bacilli | Bacillales | Bacillaceae | sf_1 | 3439 | AF071856.1 | Bacillus siralis str. 171544 |
| Bacteria | Firmicutes | Bacilli | Bacillales | Caryophanaceae | sf_1 | 3285 | AJ491302.1 | Caryophanon latum str. DSM 14151 |
| Bacteria | Firmicutes | Bacilli | Lactobacillales | Lactobacillaceae | sf_1 | 3768 | Y19167.1 | Lactobacillus perolens str. L532 |
| Bacteria | Firmicutes | Bacilli | Lactobacillales | Enterococcaceae | sf_1 | 3298 | AJ301839.1 | Enterococcus saccharolyticus str. LMG 11427 |
| Bacteria | Firmicutes | Mollicutes | Anaeroplasmatales | Erysipelotrichaceae | sf_3 | 3952 | AB055907.1 | Erysipelothrix rhusiopathiae str. Pecs 56 |
| Bacteria | Firmicutes | Clostridia | Clostridiales | Lachnospiraceae | sf_5 | 4281 | AF332721.1 | granular sludge clone UASB_brew_B86 |
| Bacteria | Firmicutes | Clostridia | Clostridiales | Lachnospiraceae | sf_5 | 4331 | AF332720.1 | granular sludge clone UASB_brew_B84 |
| Bacteria | Firmicutes | Clostridia | Clostridiales | Lachnospiraceae | sf_5 | 4567 | AJ408989.1 | human colonic clone HuCB5 |
| Bacteria | Verrucomicrobia | Verrucomicrobiae | Verrucomicrobiales | Verrucomicrobia subdivision 7 | sf_1 | 446 | AY114322.1 | anoxic marine sediment clone LD1-PA34 |
| Bacteria | Proteobacteria | Gammaproteobacteria | Enterobacteriales | Enterobacteriaceae | sf_6 | 433 | AF523903.1 | coal effluent wetland clone RCP2-6 |
| Bacteria | Planctomycetes | Planctomycetacia | Planctomycetales | Planctomycetaceae | sf_3 | 4948 | BX294756.1 | anoxic basin clone CY0ARA027D01 |
| Bacteria | Proteobacteria | Betaproteobacteria | Unclassified | Unclassified | sf_3 | 8114 |  | |
| Bacteria | Proteobacteria | Gammaproteobacteria | Thiotrichales | Thiotrichaceae | sf_3 | 9015 | AF110274.1 | Beggiatoa alba str. B18LD; ATCC 33555 |
| Bacteria | Proteobacteria | Gammaproteobacteria | Pasteurellales | Pasteurellaceae | sf_1 | 8432 |  | |
| Bacteria | Actinobacteria | Actinobacteria | Actinomycetales | Micrococcaceae | sf_1 | 1593 | M23411.1 | Arthrobacter globiformis |
| Bacteria | Actinobacteria | Actinobacteria | Actinomycetales | Actinomycetaceae | sf_1 | 1684 | AJ428402.1 | Varibaculum cambriense str. CCUG 44998 |
| Bacteria | Actinobacteria | Actinobacteria | Actinomycetales | Micrococcaceae | sf_1 | 1966 | AY133106.1 | TCE-contaminated site clone ccspost2208 |
| Bacteria | Actinobacteria | Actinobacteria | Actinomycetales | Micrococcaceae | sf_1 | 1494 | X80748.1 | Arthrobacter agilis str. DSM 20550 |
| Bacteria | Chloroflexi | Chloroflexi-4 | Unclassified | Unclassified | sf_2 | 2344 | AF507700.1 | forest soil clone C083 |
| Bacteria | Firmicutes | Clostridia | Clostridiales | Lachnospiraceae | sf_5 | 3036 | AB088990.1 | termite gut homogenate clone Rs-F27 bacterium |
| Bacteria | Firmicutes | Clostridia | Clostridiales | Lachnospiraceae | sf_5 | 3059 | X89978.1 | Butyrivibrio fibrisolvens str. NCDO 2249 |
| Bacteria | Firmicutes | Bacilli | Bacillales | Bacillaceae | sf_1 | 3579 | AB020198.1 | Bacillus sp. str. TGS750 |
| Bacteria | Firmicutes | Bacilli | Bacillales | Bacillaceae | sf_1 | 3909 | D26185.1 | Bacillus subtilis subsp. Marburg str. 168 |
| Bacteria | Firmicutes | Mollicutes | Mycoplasmatales | Mycoplasmataceae | sf_1 | 3929 | AY191226.1 | Mycoplasma gypsbengalensis str. Gb-V33 |
| Bacteria | Firmicutes | Clostridia | Clostridiales | Clostridiaceae | sf_12 | 4278 | AF482434.1 | granular sludge clone R1p16 |
| Bacteria | Firmicutes | Clostridia | Clostridiales | Clostridiaceae | sf_12 | 4622 | AB100475.1 | termite gut clone Rs-L36 |
| Bacteria | Coprothermobacteria | Unclassified | Unclassified | Unclassified | sf_1 | 751 | AJ431258.1 | Coprothermobacter sp. str. Dex80-3 |
| Bacteria | Verrucomicrobia | Verrucomicrobiae | Verrucomicrobiales | Verrucomicrobia subdivision 5 | sf_1 | 629 | AY114328.1 | anoxic marine sediment clone LD1-PA50 |
| Bacteria | Bacteroidetes | Flavobacteria | Flavobacteriales | Flavobacteriaceae | sf_1 | 6252 | AF449259.1 | Riftia pachyptila's tube clone R103-B20 |
| Bacteria | Acidobacteria | Acidobacteria | Acidobacteriales | Acidobacteriaceae | sf_14 | 6421 | AJ292576.1 | PCB-polluted soil clone WD217 |
| Bacteria | Proteobacteria | Gammaproteobacteria | Legionellales | Coxiellaceae | sf_3 | 7893 | AJ252651.1 | agricultural soil clone SC-I-71 |
| Bacteria | Proteobacteria | Gammaproteobacteria | Oceanospirillales | Saccharospirillaceae | sf_1 | 8889 | AF452603.1 | hypersaline Mono Lake clone ML110J-5 |
| Bacteria | Proteobacteria | Gammaproteobacteria | Pseudomonadales | Pseudomonadaceae | sf_1 | 9005 | AJ278108.1 | Pseudomonas sp. str. KY |
| Bacteria | Proteobacteria | Deltaproteobacteria | Desulfovibrionales | Desulfovibrionaceae | sf_1 | 9709 | AB089104.1 | termite gut homogenate clone Rs-N31 proteobacterium |
| Bacteria | OP10 | Unclassified | Unclassified | Unclassified | sf_5 | 9782 | AY192275.1 | Rocky Mountain alpine soil clone S1a-1H |
| Bacteria | Actinobacteria | Actinobacteria | Actinomycetales | Unclassified | sf_4 | 1337 | AF234118.1 | Sturt arid-zone soil clone #0425-2M17 |
| Bacteria | Actinobacteria | Actinobacteria | Acidimicrobiales | Acidimicrobiaceae | sf_1 | 1360 | AF523917.1 | forested wetland clone RCP2-103 |
| Bacteria | Firmicutes | Bacilli | Bacillales | Paenibacillaceae | sf_1 | 3299 | AF378230.1 | Brevibacillus borstelensis str. LMG 15536 |
| Bacteria | Firmicutes | Bacilli | Bacillales | Bacillaceae | sf_1 | 3706 | AF302119.1 | Bacillus sonorensis str. NRRL B-23155 |
| Bacteria | Firmicutes | Bacilli | Bacillales | Staphylococcaceae | sf_1 | 3258 | D83358.1 | Staphylococcus auricularis str. MAFF911484 ATCC33753T |
| Bacteria | Firmicutes | Bacilli | Lactobacillales | Streptococcaceae | sf_1 | 3722 | NC_002662.1 | Lactococcus Il1403 subsp. lactis str. IL1403 |
| Bacteria | Firmicutes | Clostridia | Clostridiales | Clostridiaceae | sf_12 | 4357 | AF550610.1 | Lachnospiraceae bacterium 19gly4 |
| Bacteria | Firmicutes | Clostridia | Clostridiales | Clostridiaceae | sf_12 | 4475 | AB089035.1 | termite gut homogenate clone Rs-N02 bacterium |
| Bacteria | Firmicutes | Clostridia | Clostridiales | Lachnospiraceae | sf_5 | 4533 | AB088980.1 | termite gut homogenate clone Rs-N06 bacterium |
| Bacteria | Firmicutes | Clostridia | Clostridiales | Lachnospiraceae | sf_5 | 4540 | AB088968.1 | termite gut homogenate clone Rs-M18 bacterium |
| Bacteria | Bacteroidetes | Bacteroidetes | Bacteroidales | Bacteroidaceae | sf_12 | 5320 | M86695.1 | Bacteroides distasonis |
| Bacteria | Bacteroidetes | Bacteroidetes | Bacteroidales | Unclassified | sf_15 | 5820 | AY244902.1 | cow rumen clone BF24 |
| Bacteria | Proteobacteria | Alphaproteobacteria | Sphingomonadales | Sphingomonadaceae | sf_1 | 7036 | AY026916.1 | Lutibacterium anuloederans str. LC8 |
| Bacteria | Proteobacteria | Betaproteobacteria | Burkholderiales | Oxalobacteraceae | sf_1 | 7843 | U54470.1 | Massilia timonae timone |
| Bacteria | Proteobacteria | Gammaproteobacteria | Pseudomonadales | Moraxellaceae | sf_3 | 8838 | AJ748267.1 | Psychrobacter psychrophilus CMS 28 |
| Bacteria | Actinobacteria | Actinobacteria | Actinomycetales | Micrococcaceae | sf_1 | 1213 | X87756.1 | Kocuria roseus |
| Bacteria | Actinobacteria | Actinobacteria | Actinomycetales | Corynebacteriaceae | sf_1 | 1492 | AY677186.1 | Corynebacterium tuscaniae str. ISS-5309 |
| Bacteria | Firmicutes | Clostridia | Clostridiales | Lachnospiraceae | sf_5 | 2961 | AB088989.1 | termite gut homogenate clone Rs-F92 bacterium |
| Bacteria | Verrucomicrobia | Verrucomicrobiae | Verrucomicrobiales | Verrucomicrobia subdivision 5 | sf_1 | 533 | AY114330.1 | anoxic marine sediment clone LD1-PB12 |
| Bacteria | Firmicutes | Unclassified | Unclassified | Unclassified | sf_8 | 546 | AF282254.1 | Ferribacter thermoautotrophicus |
| Bacteria | Acidobacteria | Acidobacteria | Acidobacteriales | Acidobacteriaceae | sf_14 | 508 | AJ519382.1 | uranium mining waste pile clone JG37-AG-81 sp. |
| Bacteria | Acidobacteria | Acidobacteria | Acidobacteriales | Acidobacteriaceae | sf_16 | 6414 | AF529350.1 | PCE-contaminated site clone CLs73 |
| Bacteria | Proteobacteria | Alphaproteobacteria | Caulobacterales | Caulobacteraceae | sf_1 | 7436 | AJ227797.1 | Brevundimonas sp. str. FWC40 |
| Bacteria | Proteobacteria | Gammaproteobacteria | Thiotrichales | Thiotrichaceae | sf_3 | 8752 | AF110276.1 | Beggiatoa sp. str. MS-81-1c |
| Bacteria | Proteobacteria | Unclassified | Unclassified | Unclassified | sf_21 | 8509 |  | |
| Bacteria | Proteobacteria | Deltaproteobacteria | Myxococcales | Polyangiaceae | sf_3 | 10353 | AF234747.1 | sludge clone A9 |
| Bacteria | Proteobacteria | Deltaproteobacteria | Desulfobacterales | Unclassified | sf_3 | 9813 | AF420340.1 | hydrothermal sediment clone AF420340 |
| Bacteria | Actinobacteria | Actinobacteria | Actinomycetales | Micrococcaceae | sf_1 | 1610 | X86595.1 | Arthrobacter sp str. AC-51 |
| Bacteria | Actinobacteria | Actinobacteria | Actinomycetales | Micrococcaceae | sf_1 | 2019 | AF057289.1 | Micrococcus luteus str. HN2-11 |
| Bacteria | Actinobacteria | Actinobacteria | Actinomycetales | Corynebacteriaceae | sf_1 | 1820 | U87823.1 | Corynebacterium jeikeium str. ATCC 43734 |
| Bacteria | Firmicutes | Clostridia | Clostridiales | Lachnospiraceae | sf_5 | 2834 | U41168.1 | Butyrivibrio fibrisolvens str. OB156 |
| Bacteria | Firmicutes | Clostridia | Clostridiales | Peptostreptococcaceae | sf_5 | 2694 | AY134903.1 | oral periodontitis clone FX028 |
| Bacteria | Firmicutes | Clostridia | Clostridiales | Peptostreptococcaceae | sf_5 | 393 | AF542229.1 | Anaerococcus vaginalis str. CCUG 31349 |
| Bacteria | Firmicutes | Bacilli | Bacillales | Bacillaceae | sf_1 | 3675 | AY189750.1 | Bacillus mojavensis str. M-1 |
| Bacteria | Firmicutes | Bacilli | Bacillales | Staphylococcaceae | sf_1 | 3524 | L14326.1 | Gemella haemolysans |
| Bacteria | Firmicutes | Bacilli | Lactobacillales | Aerococcaceae | sf_1 | 3553 | Y17300.1 | Desemzia incerta str. DSM 20581 |
| Bacteria | Firmicutes | Bacilli | Lactobacillales | Enterococcaceae | sf_1 | 3713 | AF061009.1 | Enterococcus cecorum str. ATCC43198 |
| Bacteria | Firmicutes | Clostridia | Clostridiales | Clostridiaceae | sf_12 | 4369 | AB089032.1 | termite gut homogenate clone Rs-N73 bacterium |
| Bacteria | Unclassified | Unclassified | Unclassified | Unclassified | sf_106 | 243 | AF027097.1 | hot spring clone OPB25 |
| Bacteria | BRC1 | Unclassified | Unclassified | Unclassified | sf_1 | 5051 | AJ390454.1 | soil clone PBS-III-24 |
| Bacteria | marine group A | mgA-1 | Unclassified | Unclassified | sf_1 | 6408 | AACY01094130.1 | Sargasso Sea |
| Bacteria | Proteobacteria | Alphaproteobacteria | Unclassified | Unclassified | sf_6 | 7575 |  | |
| Bacteria | Proteobacteria | Betaproteobacteria | Burkholderiales | Oxalobacteraceae | sf_1 | 8034 | Y08845.1 | Janthinobacterium agaricidamnosum str. W1r3T |
| Bacteria | Proteobacteria | Deltaproteobacteria | Desulfobacterales | Desulfobacteraceae | sf_5 | 10319 | AY083017.1 | sulfate-reducing habitat clone SLM-CP-116 |
| Bacteria | Proteobacteria | Deltaproteobacteria | Unclassified | Unclassified | sf_9 | 9890 | AB089106.1 | termite gut homogenate clone Rs-K70 proteobacterium |
| Bacteria | Proteobacteria | Gammaproteobacteria | Enterobacteriales | Enterobacteriaceae | sf_1 | 1206 | AY265343.1 | Dermacentor variabilis symbiont |
| Bacteria | Firmicutes | Symbiobacteria | Symbiobacterales | Unclassified | sf_3 | 3508 | AF190460.1 | Symbiobacterium toebii str. SC-1 |
| Bacteria | Firmicutes | Bacilli | Bacillales | Staphylococcaceae | sf_1 | 3684 | S83569.1 | Staphylococcus sciuri |
| Bacteria | OP10 | CH21 cluster | Unclassified | Unclassified | sf_1 | 514 | AF368184.1 | sludge clone SBRA136 |
| Bacteria | Verrucomicrobia | Verrucomicrobiae | Verrucomicrobiales | Verrucomicrobia subdivision 7 | sf_1 | 559 | AY114314.1 | anoxic marine sediment clone LD1-PA20 |
| Bacteria | Verrucomicrobia | Unclassified | Unclassified | Unclassified | sf_5 | 686 | AF419674.1 | Guaymas Basin hydrothermal sediment clone a2b018 |
| Bacteria | OP3 | Unclassified | Unclassified | Unclassified | sf_2 | 349 | AJ390463.1 | soil clone PBS-25 |
| Bacteria | Bacteroidetes | Sphingobacteria | Sphingobacteriales | Flexibacteraceae | sf_19 | 5566 | AY264838.1 | Hongiella mannitolivorans str. IMSNU 14012 JC2050 |
| Bacteria | Proteobacteria | Alphaproteobacteria | Caulobacterales | Caulobacteraceae | sf_1 | 6904 | AB021414.1 | Brevundimonas vesicularis str. IAM 12105T |
| Bacteria | Proteobacteria | Alphaproteobacteria | Rickettsiales | Unclassified | sf_1 | 7156 | AB089097.1 | termite gut homogenate clone Rs-M62 proteobacterium |
| Bacteria | Proteobacteria | Betaproteobacteria | Burkholderiales | Oxalobacteraceae | sf_1 | 7845 | AB038368.1 | Diaphorina citri symbiont |
| Bacteria | NC10 | NC10-2 | Unclassified | Unclassified | sf_1 | 10254 | AJ519650.1 | uranium mill tailings soil sample clone Sh765B-TzT-35 |
| Bacteria | Proteobacteria | Epsilonproteobacteria | Campylobacterales | Helicobacteraceae | sf_3 | 10428 | AF034135.1 | Flexispira rappini FH 9702248 |
| Bacteria | Proteobacteria | Epsilonproteobacteria | Campylobacterales | Helicobacteraceae | sf_3 | 10520 | AJ249858.1 | Helicobacter sp. blood isolate 964 |
| Bacteria | Proteobacteria | Epsilonproteobacteria | Campylobacterales | Helicobacteraceae | sf_3 | 10548 | AF286052.1 | Helicobacter rappini W.Tee-Bat |
| Bacteria | Proteobacteria | Epsilonproteobacteria | Campylobacterales | Helicobacteraceae | sf_3 | 10562 | AF286053.1 | Helicobacter rappini W.Tee-Yu |
| Bacteria | Firmicutes | Clostridia | Clostridiales | Peptococc/Acidaminococc | sf_11 | 304 | AB003379.1 | Selenomonas ruminantium str.JCM6582 |
| Bacteria | Firmicutes | Bacilli | Lactobacillales | Enterococcaceae | sf_1 | 3318 | AF539705.1 | Enterococcus ratti str. ATCC 700914 |
| Bacteria | Bacteroidetes | Sphingobacteria | Sphingobacteriales | Unclassified | sf_3 | 6298 | AF445661.1 | travertine hot spring clone SM1C04 |
| Bacteria | Chlorobi | Chlorobia | Chlorobiales | Chlorobiaceae | sf_1 | 262 | Y18253.1 | Chlorobium ferrooxidans DSM 13031 str. KofoX |
| Bacteria | Proteobacteria | Gammaproteobacteria | Pasteurellales | Pasteurellaceae | sf_1 | 8409 | AF499896.1 | human colonic mucosal biopsy clone ABLCf1 |
| Bacteria | Proteobacteria | Deltaproteobacteria | Desulfovibrionales | Desulfovibrionaceae | sf_1 | 10016 | AB089110.1 | termite gut homogenate clone Rs-N35 proteobacterium |
| Bacteria | Firmicutes | Clostridia | Clostridiales | Lachnospiraceae | sf_5 | 2698 | AB088950.1 | termite gut homogenate clone Rs-B88 bacterium |
| Bacteria | Firmicutes | Clostridia | Clostridiales | Peptostreptococcaceae | sf_5 | 3080 | AB088986.1 | termite gut homogenate clone Rs-F43 bacterium |
| Bacteria | Firmicutes | Bacilli | Bacillales | Bacillaceae | sf_1 | 305 | M77488.1 | Bacillus thermoleovorans |
| Bacteria | Firmicutes | Bacilli | Lactobacillales | Enterococcaceae | sf_1 | 3382 |  | |
| Bacteria | Firmicutes | Unclassified | Unclassified | Unclassified | sf_8 | 4536 | AF507891.1 | Mono Lake at depth 35m station 6 July 2000 clone ML635J-14 G+C |
| Bacteria | Firmicutes | Clostridia | Clostridiales | Lachnospiraceae | sf_5 | 4511 | AF376218.1 | ckncm314-B7-17 clone |
| Bacteria | Firmicutes | Clostridia | Clostridiales | Clostridiaceae | sf_12 | 4566 | AF371796.1 | swine intestine clone p-2657-65A5 |
| Bacteria | Bacteroidetes | Bacteroidetes | Bacteroidales | Bacteroidaceae | sf_12 | 5256 | AB088925.1 | termite gut homogenate clone Rs-D38 bacterium |
| Bacteria | Bacteroidetes | Bacteroidetes | Bacteroidales | Prevotellaceae | sf_1 | 6047 | AY093462.1 | deep marine sediment clone MB-A2-107 |
| Bacteria | Proteobacteria | Alphaproteobacteria | Sphingomonadales | Sphingomonadaceae | sf_1 | 7440 | AF159257.2 | Sphingobium chungbukense str. DJ77 |
| Bacteria | Proteobacteria | Deltaproteobacteria | Myxococcales | Polyangiaceae | sf_3 | 10249 | AJ534629.1 | soil sample uranium mining waste pile near town Johanngeorgenstadt clone JG36-TzT-168 proteobacterium |
| Bacteria | Actinobacteria | Actinobacteria | Actinomycetales | Micrococcaceae | sf_1 | 1724 | X95483.1 | Rothia mucilaginosa str. DSM |
| Bacteria | Firmicutes | Clostridia | Clostridiales | Lachnospiraceae | sf_5 | 3042 | AF371584.1 | swine intestine clone p-2876-6C5 |
| Bacteria | Firmicutes | Bacilli | Lactobacillales | Unclassified | sf_1 | 3481 |  | |
| Bacteria | Chloroflexi | Anaerolineae | Unclassified | Unclassified | sf_9 | 727 | AF507692.1 | forest soil clone S0208 |
| Bacteria | Proteobacteria | Alphaproteobacteria | Caulobacterales | Caulobacteraceae | sf_1 | 6781 | AB023784.1 | Brevundimonas intermedia str. MBIC2712 ATCC15262 |
| Bacteria | Proteobacteria | Betaproteobacteria | Burkholderiales | Alcaligenaceae | sf_1 | 8094 | AF430122.1 | Alcaligenes sp. str. VKM B-2263 dcm6 |
| Bacteria | Actinobacteria | Actinobacteria | Bifidobacteriales | Bifidobacteriaceae | sf_1 | 1351 | AY174108.1 | Bifidobacterium psychraerophilum str. T16 |
| Bacteria | Firmicutes | Clostridia | Clostridiales | Peptococc/Acidaminococc | sf_11 | 710 | AF458222.1 | Centipeda periodontii str. HB-2 |
| Bacteria | Firmicutes | Clostridia | Clostridiales | Lachnospiraceae | sf_5 | 3076 | AY169415.1 | Clostridium nexile |
| Bacteria | Firmicutes | Clostridia | Clostridiales | Peptostreptococcaceae | sf_5 | 2805 | AY134904.1 | oral periodontitis clone FX033 |
| Bacteria | Firmicutes | Clostridia | Clostridiales | Clostridiaceae | sf_12 | 3077 | X76750.1 | Clostridium glycolicum str. DSM 1288 |
| Bacteria | Firmicutes | Clostridia | Clostridiales | Peptostreptococcaceae | sf_5 | 224 | AB109771.1 | Finegoldia magna str. ATCC 29328 |
| Bacteria | Firmicutes | Bacilli | Bacillales | Staphylococcaceae | sf_1 | 3628 | X66100.1 | Staphylococcus haemolyticus str. CCM2737 |
| Bacteria | Firmicutes | Bacilli | Lactobacillales | Lactobacillaceae | sf_1 | 3566 | X76329.1 | Lactobacillus pontis str. LTH 2587 |
| Bacteria | Firmicutes | Clostridia | Clostridiales | Clostridiaceae | sf_12 | 4300 | AB100493.1 | termite gut clone Rs-060 |
| Bacteria | Bacteroidetes | Bacteroidetes | Bacteroidales | Prevotellaceae | sf_1 | 5484 | AY134905.1 | oral periodontitis clone FX046 |
| Bacteria | Bacteroidetes | Flavobacteria | Flavobacteriales | Blattabacteriaceae | sf_1 | 5828 | Z35664.1 | Blattabacterium species |
| Bacteria | Proteobacteria | Gammaproteobacteria | Thiotrichales | Thiotrichaceae | sf_3 | 8703 | AF110275.1 | Beggiatoa sp. str. AA5A |
| Bacteria | Proteobacteria | Alphaproteobacteria | Unclassified | Unclassified | sf_6 | 8780 | AJ296549.1 | uranium mining mill tailing clone GR-296.II.89 GR-296.II.89 |
| Bacteria | Proteobacteria | Deltaproteobacteria | Desulfovibrionales | Desulfovibrionaceae | sf_1 | 10248 | AF418170.1 | Desulfovibrio giganteus str. DSM 4370 |
| Bacteria | Firmicutes | Bacilli | Bacillales | Bacillaceae | sf_1 | 3836 | AY682096.1 | Geobacillus stearothermophilus str. 46 |
| Bacteria | Firmicutes | Bacilli | Bacillales | Bacillaceae | sf_1 | 3831 | AY030328.1 | Bacillus licheniformis str. KL-068 |
| Bacteria | Firmicutes | Bacilli | Lactobacillales | Lactobacillaceae | sf_1 | 3634 | AJ417738.1 | Lactobacillus letivazi str. JCL3994 |
| Bacteria | Firmicutes | Bacilli | Lactobacillales | Enterococcaceae | sf_1 | 3598 | AJ301840.1 | Enterococcus solitarius str. DSM 5634 |
| Bacteria | Firmicutes | Bacilli | Lactobacillales | Streptococcaceae | sf_1 | 3313 | AY188352.1 | Streptococcus salivarius str. ATCC 7073 |
| Bacteria | Bacteroidetes | Bacteroidetes | Bacteroidales | Rikenellaceae | sf_5 | 5892 | AJ229217.1 | anoxic bulk soil flooded rice microcosm clone BSV73 |
| Bacteria | Bacteroidetes | Flavobacteria | Flavobacteriales | Flavobacteriaceae | sf_1 | 5971 | M62799.1 | Cytophaga uliginosa |
| Bacteria | Cyanobacteria | Cyanobacteria | Chloroplasts | Chloroplasts | sf_13 | 5000 | U67742.1 | Mitrastema yamamotoi |
| Bacteria | Proteobacteria | Gammaproteobacteria | SUP05 | Unclassified | sf_1 | 8605 | AF382104.1 | bacterioplankton clone ZA2525c |
| Bacteria | Proteobacteria | Epsilonproteobacteria | Campylobacterales | Helicobacteraceae | sf_3 | 10432 | AF449246.1 | Riftia pachyptila's tube clone R76-B51 |
| Bacteria | Firmicutes | Bacilli | Bacillales | Staphylococcaceae | sf_1 | 3284 |  | |
| Bacteria | Firmicutes | Bacilli | Lactobacillales | Enterococcaceae | sf_1 | 3261 | AJ301836.1 | Enterococcus mundtii str. LMG 10748 |
| Bacteria | Firmicutes | Clostridia | Clostridiales | Lachnospiraceae | sf_5 | 4510 | AB089034.1 | termite gut homogenate clone Rs-Q53 bacterium |
| Bacteria | Verrucomicrobia | Unclassified | Unclassified | Unclassified | sf_3 | 486 | AJ401113.1 | Elbe river clone DEV045 |
| Bacteria | Verrucomicrobia | Verrucomicrobiae | Verrucomicrobiales | Verrucomicrobia subdivision 5 | sf_1 | 530 | AY114334.1 | anoxic marine sediment clone LD1-PB20 |
| Bacteria | Verrucomicrobia | Verrucomicrobiae | Verrucomicrobiales | Unclassified | sf_3 | 792 | AB089122.1 | termite gut homogenate clone Rs-P07 bacterium |
| Bacteria | Bacteroidetes | Bacteroidetes | Bacteroidales | Porphyromonadaceae | sf_1 | 6012 | AJ400264.1 | mouse feces clone L11-6 |
| Bacteria | Bacteroidetes | Sphingobacteria | Sphingobacteriales | Flammeovirgaceae | sf_5 | 6084 | AB078078.1 | Microscilla arenaria str. IFO 15982 |
| Bacteria | Bacteroidetes | Sphingobacteria | Sphingobacteriales | Flexibacteraceae | sf_19 | 5372 |  | |
| Bacteria | Chloroflexi | Anaerolineae | Unclassified | Unclassified | sf_9 | 946 | AY216458.1 | temperate estuarine mud clone KM87 |
| Bacteria | Proteobacteria | Betaproteobacteria | Burkholderiales | Ralstoniaceae | sf_1 | 7778 | AJ539233.1 | Ralstonia insidiosa str. CCUG 46388 |
| Bacteria | Proteobacteria | Epsilonproteobacteria | Campylobacterales | Helicobacteraceae | sf_3 | 10462 | U96297.1 | Helicobacter rodentium str. MIT 96-1312 |
| Bacteria | Proteobacteria | Epsilonproteobacteria | Campylobacterales | Campylobacteraceae | sf_3 | 10456 | L06975.1 | Campylobacter showae |
| Bacteria | Actinobacteria | Actinobacteria | Rubrobacterales | Rubrobacteraceae | sf_1 | 1843 | AJ536866.1 | uranium mining waste pile soil sample clone JG30-KF-A23 |
| Bacteria | Firmicutes | Clostridia | Clostridiales | Peptococc/Acidaminococc | sf_11 | 39 | AF523919.1 | forested wetland clone RCP2-71 |
| Bacteria | Verrucomicrobia | Verrucomicrobiae | Verrucomicrobiales | Verrucomicrobia subdivision 7 | sf_1 | 760 | AF454310.2 | Mono lake clone ML316M-1 |
| Bacteria | Synergistes | Unclassified | Unclassified | Unclassified | sf_3 | 719 | AY207056.1 | Synergistes sp. P1 str. P4G_18 |
| Bacteria | Bacteroidetes | Flavobacteria | Flavobacteriales | Flavobacteriaceae | sf_1 | 5997 | M62797.1 | Flavobacterium aquatile |
| Bacteria | Planctomycetes | Planctomycetacia | Planctomycetales | Anammoxales | sf_2 | 4683 | BX294785.1 | anoxic basin clone CY0ARA028B09 |
| Bacteria | Proteobacteria | Gammaproteobacteria | Enterobacteriales | Enterobacteriaceae | sf_1 | 8236 | AF476110.1 | Vryburgia amaryllidis symbiont |
| Bacteria | Proteobacteria | Epsilonproteobacteria | Campylobacterales | Helicobacteraceae | sf_3 | 10444 | AB006148.1 | Helicobacter suncus str. Kaz-2 |
| Bacteria | Proteobacteria | Epsilonproteobacteria | Campylobacterales | Helicobacteraceae | sf_3 | 10552 | AF363062.1 | Helicobacter winghamensis str. NLEP 97-1611 |
| Bacteria | Firmicutes | Clostridia | Clostridiales | Clostridiaceae | sf_12 | 3021 | AF458779.1 | Clostridium caminithermale str. DVird3 |
| Bacteria | Firmicutes | Clostridia | Clostridiales | Clostridiaceae | sf_12 | 4364 | AF481208.1 | oral endodontic infection clone MCE3_9 |
| Bacteria | Natronoanaerobium | Unclassified | Unclassified | Unclassified | sf_1 | 4377 | AF507888.1 | Mono Lake at depth 35m station 6 July 2000 clone ML635J-65 G+C |
| Bacteria | Bacteroidetes | Bacteroidetes | Bacteroidales | Prevotellaceae | sf_1 | 5905 | AF371893.1 | swine intestine clone p-2443-18B5 |
| Bacteria | Proteobacteria | Alphaproteobacteria | Caulobacterales | Caulobacteraceae | sf_1 | 7359 | AJ227782.1 | Brevundimonas bacteroides str. CB7 |
| Bacteria | Proteobacteria | Alphaproteobacteria | Caulobacterales | Caulobacteraceae | sf_1 | 7366 | AJ227784.1 | Brevundimonas subvibrioides str. CB81 |
| Bacteria | Proteobacteria | Gammaproteobacteria | Pasteurellales | Pasteurellaceae | sf_1 | 9237 |  | |
| Bacteria | WS3 | Unclassified | Unclassified | Unclassified | sf_1 | 2537 | AY114325.1 | anoxic marine sediment clone LD1-PA39 |
| Bacteria | Firmicutes | Clostridia | Clostridiales | Peptococc/Acidaminococc | sf_11 | 242 | AJ493052.1 | Desulfosporosinus orientis str. DSMZ 7493 |
| Bacteria | Firmicutes | Bacilli | Bacillales | Bacillaceae | sf_1 | 3900 | X68416.1 | Bacillus licheniformis str. DSM 13 |
| Bacteria | Bacteroidetes | Bacteroidetes | Bacteroidales | Porphyromonadaceae | sf_1 | 5510 | AF524856.1 | sphagnum peat bog clone 26-4b2 |
| Bacteria | Chlorobi | Unclassified | Unclassified | Unclassified | sf_9 | 6146 | AF234699.1 | sludge clone A12b |
| Bacteria | Unclassified | Unclassified | Unclassified | Unclassified | sf_140 | 6355 |  | |
| Bacteria | Proteobacteria | Alphaproteobacteria | Caulobacterales | Caulobacteraceae | sf_1 | 6909 | X87274.1 | Brevundimonas diminuta str. DSM 1635 |
| Bacteria | Actinobacteria | Actinobacteria | Actinomycetales | Kineosporiaceae | sf_1 | 1961 | X77958.1 | Kineococcus aurantiacus str. IFO 15268 |
| Bacteria | Firmicutes | Clostridia | Unclassified | Unclassified | sf_3 | 2373 |  | |
| Bacteria | Firmicutes | Symbiobacteria | Symbiobacterales | Unclassified | sf_1 | 2388 | AF465653.1 | G+C Gram-positive clone YNPRH70A |
| Bacteria | Chloroflexi | Dehalococcoidetes | Unclassified | Unclassified | sf_1 | 2485 |  | |
| Bacteria | Firmicutes | Clostridia | Clostridiales | Peptostreptococcaceae | sf_5 | 2797 | AY167963.1 | Isolation and identification hyper-ammonia producing swine storage pits manure |
| Bacteria | Firmicutes | Bacilli | Bacillales | Bacillaceae | sf_1 | 3763 | AY672761.1 | Geobacillus stearothermophilus |
| Bacteria | Firmicutes | Bacilli | Lactobacillales | Streptococcaceae | sf_1 | 3629 | AF139600.1 | Streptococcus mutans str. UA96 |
| Bacteria | Verrucomicrobia | Verrucomicrobiae | Verrucomicrobiales | Verrucomicrobia subdivision 5 | sf_1 | 547 | AY114329.1 | anoxic marine sediment clone LD1-PB1 |
| Bacteria | Bacteroidetes | Unclassified | Unclassified | Unclassified | sf_4 | 5787 | AF507860.1 | Mono Lake at depth 35 m station 6 July 2000 clone ML635J-1 bacterium |
| Bacteria | Firmicutes | Bacilli | Lactobacillales | Enterococcaceae | sf_1 | 3881 | AJ301829.1 | Enterococcus dispar str. LMG 13521 |
| Bacteria | Firmicutes | Clostridia | Clostridiales | Clostridiaceae | sf_12 | 4272 | AB088965.1 | termite gut homogenate clone Rs-M34 bacterium |
| Bacteria | Firmicutes | Clostridia | Clostridiales | Clostridiaceae | sf_12 | 4524 | AB100476.1 | termite gut clone Rs-093 |
| Bacteria | Firmicutes | Clostridia | Clostridiales | Lachnospiraceae | sf_5 | 4613 | AB034003.1 | rumen clone 3C0d-3 |
| Bacteria | Acidobacteria | Acidobacteria | Acidobacteriales | Acidobacteriaceae | sf_6 | 6345 | AJ534634.1 | soil sample uranium mining waste pile near town Johanngeorgenstadt clone JG36-TzT-77 bacterium |
| Bacteria | Proteobacteria | Gammaproteobacteria | Alteromonadales | Alteromonadaceae | sf_1 | 8978 | AF468393.1 | Arctic sea ice ARK10108 |
| Bacteria | Lentisphaerae | Unclassified | Unclassified | Unclassified | sf_5 | 10027 | AJ431234.1 | Cytophaga sp. str. Dex80-43 |
| Bacteria | Lentisphaerae | Unclassified | Unclassified | Unclassified | sf_5 | 10330 | AF507900.1 | Mono lake clone ML635J-58 |
| Bacteria | Lentisphaerae | Unclassified | Unclassified | Unclassified | sf_5 | 9704 | AJ431235.1 | Cytophaga sp. str. Dex80-64 |
| Bacteria | Chloroflexi | Dehalococcoidetes | Unclassified | Unclassified | sf_1 | 2438 | AY093464.1 | deep marine sediment clone MB-A2-110 |
| Bacteria | Firmicutes | Clostridia | Clostridiales | Lachnospiraceae | sf_5 | 3218 | AB089000.1 | termite gut homogenate clone Rs-N53 |
| Bacteria | Firmicutes | Clostridia | Clostridiales | Clostridiaceae | sf_12 | 2764 |  | |
| Bacteria | Firmicutes | Bacilli | Lactobacillales | Enterococcaceae | sf_1 | 3433 | D88824.1 | Tetragenococcus muriaticus |
| Bacteria | Unclassified | Unclassified | Unclassified | Unclassified | sf_160 | 6456 |  | |
| Bacteria | Proteobacteria | Alphaproteobacteria | Unclassified | Unclassified | sf_6 | 7377 | AY192273.1 | Rocky Mountain alpine soil clone W2b-8C |
| Bacteria | Unclassified | Unclassified | Unclassified | Unclassified | sf_160 | 10012 |  | |
| Bacteria | Unclassified | Unclassified | Unclassified | Unclassified | sf_95 | 2545 | AB106352.1 | anaerobic sludge isolate str. JE |
| Bacteria | Firmicutes | Clostridia | Clostridiales | Peptostreptococcaceae | sf_5 | 2679 | AB062845.1 | termite gut homogenate clone BCf9-13 |
| Bacteria | OP10 | CH21 cluster | Unclassified | Unclassified | sf_1 | 326 | AY222300.1 | geothermal clone ST01-SN3H |
| Bacteria | Bacteroidetes | Bacteroidetes | Bacteroidales | Porphyromonadaceae | sf_1 | 5295 | AF371910.1 | swine intestine clone p-987-s962-5 |
| Bacteria | Bacteroidetes | Flavobacteria | Flavobacteriales | Unclassified | sf_3 | 5248 | AY274839.1 | Delaware River estuary clone 1G12 |
| Bacteria | Bacteroidetes | Sphingobacteria | Sphingobacteriales | Sphingobacteriaceae | sf_1 | 5913 | AF409002.1 | Sphingobacteriaceae str. Ellin160 |
| Bacteria | Cyanobacteria | Unclassified | Unclassified | Unclassified | sf_8 | 5206 |  | |
| Bacteria | Acidobacteria | Acidobacteria | Acidobacteriales | Acidobacteriaceae | sf_6 | 6423 | AF523979.1 | coal effluent wetland clone FW92 |
| Bacteria | Proteobacteria | Betaproteobacteria | Nitrosomonadales | Nitrosomonadaceae | sf_1 | 7789 |  | |
| Bacteria | Proteobacteria | Gammaproteobacteria | Legionellales | Unclassified | sf_1 | 9418 | AJ518784.1 | uranium mining waste pile clone JG37-AG-14 proteobacterium |
| Bacteria | Proteobacteria | Gammaproteobacteria | Pseudomonadales | Moraxellaceae | sf_3 | 8366 | AJ609556.1 | Psychrobacter frigidicola str. DSM 12411 |
| Bacteria | Firmicutes | Clostridia | Clostridiales | Unclassified | sf_17 | 2324 |  | |
| Bacteria | AD3 | Unclassified | Unclassified | Unclassified | sf_1 | 2338 | AJ536867.1 | uranium mining waste pile soil clone JG30-KF-C12 |
| Bacteria | Firmicutes | Clostridia | Clostridiales | Peptococc/Acidaminococc | sf_11 | 709 | AB017195.1 | Selenomonas ruminantium str.S20 |
| Bacteria | Firmicutes | Bacilli | Bacillales | Bacillaceae | sf_1 | 3827 | AF547209.1 | Bacillus acidogenesis str. 105-2 |
| Bacteria | Firmicutes | Bacilli | Bacillales | Halobacillaceae | sf_1 | 3344 | AY881246.1 | Halobacillus yeomjeoni str. MSS-402 |
| Bacteria | Firmicutes | Bacilli | Lactobacillales | Streptococcaceae | sf_1 | 3869 | AB104843.1 | Streptococcus equi subsp. zooepidemicus str. Tokyo1291 subsp. |
| Bacteria | Deinococcus-Thermus | Unclassified | Unclassified | Unclassified | sf_3 | 920 |  | |
| Bacteria | Verrucomicrobia | Unclassified | Unclassified | Unclassified | sf_4 | 288 | U60012.1 | Prosthecobacter dejongeii |
| Bacteria | Bacteroidetes | Bacteroidetes | Bacteroidales | Prevotellaceae | sf_1 | 6011 | AB185583.1 | rumen clone F24-B03 |
| Bacteria | Bacteroidetes | Bacteroidetes | Bacteroidales | Porphyromonadaceae | sf_1 | 5460 | AJ400267.2 | mouse feces clone F8 |
| Bacteria | Acidobacteria | Acidobacteria | Acidobacteriales | Acidobacteriaceae | sf_6 | 6359 | AF529322.1 | PCE-contaminated site clone CLi114 |
| Bacteria | Proteobacteria | Deltaproteobacteria | Bdellovibrionales | Unclassified | sf_1 | 7382 | AF355039.1 | marine clone Arctic95C-5 |
| Bacteria | Actinobacteria | Actinobacteria | Actinomycetales | Unclassified | sf_3 | 1243 | AB089076.1 | termite gut homogenate clone Rs-M95 bacterium |
| Bacteria | OP9/JS1 | OP9 | Unclassified | Unclassified | sf_1 | 969 | AJ306782.1 | DCP-dechlorinating consortium clone SHA-1 |
| Bacteria | Acidobacteria | Acidobacteria-5 | Unclassified | Unclassified | sf_1 | 523 | AF245036.1 | soil metagenomic library clone 17F9 |
| Bacteria | Proteobacteria | Gammaproteobacteria | Alteromonadales | Alteromonadaceae | sf_1 | 8970 | AJ295716.1 | Arctic seawater isolate str. R9879 |
| Bacteria | Proteobacteria | Epsilonproteobacteria | Campylobacterales | Helicobacteraceae | sf_3 | 10436 | AF297868.1 | Helicobacter aurati str. MIT 97-5075c |
| Bacteria | Proteobacteria | Epsilonproteobacteria | Campylobacterales | Unclassified | sf_1 | 10480 | AJ441204.1 | hydrothermal vent polychaete mucous clone P. palm C 84 |
| Bacteria | Firmicutes | Symbiobacteria | Symbiobacterales | Unclassified | sf_1 | 77 | AF391988.1 | thermal soil clone YNPFFP9 |
| Bacteria | OP3 | Unclassified | Unclassified | Unclassified | sf_4 | 628 | AY050598.1 | CB-contaminated groundwater clone GOUTB15 |
| Bacteria | Bacteroidetes | Bacteroidetes | Bacteroidales | Porphyromonadaceae | sf_1 | 5454 | AY643492.1 | Dysgonomonas wimpennyi str. ANFA2 |
| Bacteria | Bacteroidetes | Unclassified | Unclassified | Unclassified | sf_4 | 5785 | AF507862.1 | Mono Lake at depth 35 m station 6 July 2000 clone ML635J-56 |
| Bacteria | TM7 | TM7-3 | Unclassified | Unclassified | sf_1 | 8155 | AY134895.1 | oral periodontitis clone EW086 |
| Bacteria | Actinobacteria | Actinobacteria | Actinomycetales | Micrococcaceae | sf_1 | 1557 | X83408.1 | Arthrobacter oxydans str. DSM 20119 |
| Bacteria | Firmicutes | Clostridia | Clostridiales | Peptococc/Acidaminococc | sf_11 | 940 | X84006.1 | Veillonella dispar str. DSM 20735 |
| Bacteria | Firmicutes | Bacilli | Bacillales | Bacillaceae | sf_1 | 3328 | AJ224963.1 | Pseudobacillus carolinae |
| Bacteria | Firmicutes | Bacilli | Lactobacillales | Streptococcaceae | sf_1 | 3253 | AF349918.1 | derived cheese sample clone 32CR |
| Bacteria | Firmicutes | Bacilli | Lactobacillales | Streptococcaceae | sf_1 | 3588 | AY188350.1 | Streptococcus downei str. ATCC 33748 |
| Bacteria | Firmicutes | Mollicutes | Anaeroplasmatales | Erysipelotrichaceae | sf_3 | 768 |  | |
| Bacteria | Firmicutes | Catabacter | Unclassified | Unclassified | sf_4 | 4503 | AB088976.1 | termite gut homogenate clone Rs-H83 bacterium |
| Bacteria | Chloroflexi | Thermomicrobia | Unclassified | Unclassified | sf_1 | 1041 | AY250886.1 | Antarctic cryptoendolith clone FBP471 |
| Bacteria | Spirochaetes | Spirochaetes | Spirochaetales | Leptospiraceae | sf_3 | 6496 | NC_005823.1 | Leptospira interrogans serovar Copenhageni str. Fiocruz L1-130 |
| Bacteria | Proteobacteria | Gammaproteobacteria | Thiotrichales | Piscirickettsiaceae | sf_3 | 9291 | AF384373.1 | Methylophaga alcalica str. M39 |
| Bacteria | Proteobacteria | Deltaproteobacteria | Desulfovibrionales | Unclassified | sf_1 | 9828 | AB089109.1 | termite gut homogenate clone Rs-M89 proteobacterium |
| Bacteria | Proteobacteria | Epsilonproteobacteria | Campylobacterales | Unclassified | sf_1 | 10489 | AF299121.1 | S17sBac16 complete clone |
| Bacteria | Proteobacteria | Epsilonproteobacteria | Campylobacterales | Helicobacteraceae | sf_3 | 10467 |  | |
| Bacteria | Chloroflexi | Dehalococcoidetes | Unclassified | Unclassified | sf_1 | 2367 | AY093473.1 | deep marine sediment clone MB-B2-113 |
| Bacteria | Firmicutes | Clostridia | Clostridiales | Peptostreptococcaceae | sf_5 | 2714 | AB088954.2 | termite gut homogenate clone Rs-N27 bacterium |
| Bacteria | Firmicutes | Bacilli | Bacillales | Bacillaceae | sf_1 | 3612 | AB042060.1 | Bacillus schlegelii str. ATCC 43741T |
| Bacteria | Firmicutes | Clostridia | Clostridiales | Unclassified | sf_17 | 3476 |  | |
| Bacteria | Firmicutes | Bacilli | Lactobacillales | Enterococcaceae | sf_1 | 3288 | AY167946.1 | Isolation and identification hyper-ammonia producing swine storage pits manure |
| Bacteria | Bacteroidetes | Bacteroidetes | Bacteroidales | Unclassified | sf_15 | 5890 | AY218551.1 | penguin droppings sediments clone KD1-125 |
| Bacteria | Proteobacteria | Gammaproteobacteria | Legionellales | Unclassified | sf_3 | 8587 | AF526927.2 | Mars Odyssey Orbiter and encapsulation facility clone T5-3 |
| Bacteria | Proteobacteria | Epsilonproteobacteria | Campylobacterales | Helicobacteraceae | sf_3 | 10454 | L36143.1 | Helicobacter pullorum str. NCTC 12826 |
| Bacteria | Firmicutes | Desulfotomaculum | Unclassified | Unclassified | sf_1 | 2351 | Y11574.1 | Desulfotomaculum thermobenzoicum str. DSM 6193 |
| Bacteria | Firmicutes | Desulfotomaculum | Unclassified | Unclassified | sf_1 | 2443 | Y11573.1 | Desulfotomaculum thermoacetoxidans str. DSM 5813 |
| Bacteria | Firmicutes | Desulfotomaculum | Unclassified | Unclassified | sf_1 | 2490 | AY084078.1 | Desulfotomaculum solfataricum str. V21 |
| Bacteria | Firmicutes | Clostridia | Clostridiales | Lachnospiraceae | sf_5 | 2931 | AB088994.1 | termite gut homogenate clone Rs-G77 bacterium |
| Bacteria | Firmicutes | Bacilli | Bacillales | Alicyclobacillaceae | sf_1 | 3368 | AY529492.1 | geothermal site isolate str. G1 |
| Bacteria | Firmicutes | Bacilli | Lactobacillales | Streptococcaceae | sf_1 | 3250 | AF396920.1 | Streptococcus bovis str. B315 |
| Bacteria | Firmicutes | Bacilli | Lactobacillales | Streptococcaceae | sf_1 | 3906 | AF104114.1 | Streptococcus bovis str.ATCC 43143 |
| Bacteria | Firmicutes | Bacilli | Lactobacillales | Streptococcaceae | sf_1 | 3251 | AY188347.1 | Streptococcus cristatus str. ATCC 51100 |
| Bacteria | Firmicutes | Clostridia | Unclassified | Unclassified | sf_7 | 4216 |  | |
| Bacteria | Synergistes | Unclassified | Unclassified | Unclassified | sf_3 | 601 | AF229792.1 | terephthalate-degrading consortium clone TA19 |
| Bacteria | Planctomycetes | Planctomycetacia | Planctomycetales | Pirellulae | sf_3 | 4670 |  | |
| Bacteria | Proteobacteria | Betaproteobacteria | Burkholderiales | Burkholderiaceae | sf_1 | 7747 |  | |
| Bacteria | Proteobacteria | Gammaproteobacteria | Unclassified | Unclassified | sf_3 | 8339 | AY212729.1 | water 5 m downstream manure clone 35ds5 |
| Bacteria | Proteobacteria | Deltaproteobacteria | Desulfovibrionales | Desulfohalobiaceae | sf_1 | 9894 | AF524933.1 | Desulfonauticus submarinus str. 6N |
| Bacteria | Actinobacteria | Actinobacteria | Bifidobacteriales | Bifidobacteriaceae | sf_1 | 1444 | AY278612.1 | Bifidobacteriaceae genomosp. C1 |
| Bacteria | Firmicutes | Clostridia | Clostridiales | Peptostreptococcaceae | sf_5 | 58 | AF481225.1 | Peptostreptococcus sp. str. E3_32 |
| Bacteria | Firmicutes | Bacilli | Lactobacillales | Enterococcaceae | sf_1 | 3392 | Y17152.2 | Vagococcus lutrae str. m1134/97/1; CCUG 39187 |
| Bacteria | Firmicutes | Clostridia | Clostridiales | Unclassified | sf_17 | 4168 |  | |
| Bacteria | Firmicutes | Clostridia | Clostridiales | Clostridiaceae | sf_12 | 4502 |  | |
| Bacteria | Chloroflexi | Unclassified | Unclassified | Unclassified | sf_5 | 1051 | AY913277.1 | forest soil clone DUNssu055 (-2B) (OTU#087) |
| Bacteria | Verrucomicrobia | Verrucomicrobiae | Verrucomicrobiales | Xiphinematobacteraceae | sf_3 | 888 | AF217462.1 | Candidatus Xiphinematobacter brevicolli |
| Bacteria | BRC1 | Unclassified | Unclassified | Unclassified | sf_2 | 118 | AY218548.1 | penguin droppings sediments clone KD1-1 |
| Bacteria | Bacteroidetes | Bacteroidetes | Bacteroidales | Unclassified | sf_15 | 5573 | AB088930.1 | termite gut homogenate clone Rs-D44 bacterium |
| Bacteria | Cyanobacteria | Cyanobacteria | Plectonema | Unclassified | sf_1 | 5190 | AF091110.1 | Plectonema sp. str. F3 |
| Bacteria | Proteobacteria | Epsilonproteobacteria | Campylobacterales | Helicobacteraceae | sf_3 | 10385 |  | |
| Bacteria | Proteobacteria | Epsilonproteobacteria | Campylobacterales | Helicobacteraceae | sf_3 | 10442 | AF292378.1 | Helicobacter cetorum str. MIT 99-5656 |
| Bacteria | Proteobacteria | Epsilonproteobacteria | Campylobacterales | Helicobacteraceae | sf_3 | 10448 | U51870.1 | Helicobacter felis str. Dog-1 |
| Bacteria | Proteobacteria | Epsilonproteobacteria | Campylobacterales | Helicobacteraceae | sf_3 | 10451 | AF506779.1 | Helicobacter heilmannii str. C4S |
| Bacteria | Actinobacteria | Actinobacteria | Actinomycetales | Corynebacteriaceae | sf_1 | 1374 |  | |
| Bacteria | Firmicutes | Bacilli | Lactobacillales | Streptococcaceae | sf_1 | 3397 | Z94012.1 | Streptococcus macedonicus str. ACA-DC 206 LAB617 |
| Bacteria | Natronoanaerobium | Unclassified | Unclassified | Unclassified | sf_1 | 769 | AJ431345.1 | fjord ikaite column clone un-c23 |
| Bacteria | Chloroflexi | Anaerolineae | Chloroflexi-1a | Unclassified | sf_1 | 258 | AJ306793.1 | DCP-dechlorinating consortium clone SHD-14 |
| Bacteria | Cyanobacteria | Cyanobacteria | Chloroplasts | Chloroplasts | sf_5 | 4967 | AF534438.1 | Toolik Lake main station at 3 m depth clone TLM14 |
| Bacteria | Proteobacteria | Alphaproteobacteria | Sphingomonadales | Sphingomonadaceae | sf_1 | 6663 | AY554010.1 | Sphingopyxis flavimaris str. SW-151 |
| Bacteria | Proteobacteria | Gammaproteobacteria | Xanthomonadales | Xanthomonadaceae | sf_3 | 8689 | AB110496.1 | Dyemonas todaii str. XD10 |
| Bacteria | Proteobacteria | Gammaproteobacteria | Unclassified | Unclassified | sf_4 | 8855 |  | |
| Bacteria | Firmicutes | Bacilli | Lactobacillales | Streptococcaceae | sf_1 | 3290 | AY518677.1 | Streptococcus mitis str. Sm91 |
| Bacteria | Firmicutes | Mollicutes | Anaeroplasmatales | Erysipelotrichaceae | sf_3 | 3981 | AY128088.1 | phototrophic sludge clone PSB-M-3 |
| Bacteria | Bacteroidetes | Bacteroidetes | Bacteroidales | Unclassified | sf_15 | 5783 | AF507869.2 | Mono Lake at depth 35 m station 6 July 2000 clone ML635J-15 bacterium |
| Bacteria | Actinobacteria | Actinobacteria | Acidimicrobiales | Acidimicrobiaceae | sf_1 | 1749 | AY913475.1 | forest soil clone DUNssu275 (-3A) (OTU#188) |
| Bacteria | Firmicutes | Clostridia | Clostridiales | Lachnospiraceae | sf_5 | 3060 | AB089002.1 | termite gut homogenate clone Rs-B14 bacterium |
| Bacteria | Firmicutes | Bacilli | Lactobacillales | Streptococcaceae | sf_1 | 3753 | AF009482.1 | Streptococcus suis str. 8074 |
| Bacteria | Firmicutes | Bacilli | Lactobacillales | Streptococcaceae | sf_1 | 3287 | AF432137.1 | tongue dorsum scrapings clone FP015 |
| Bacteria | Firmicutes | Mollicutes | Anaeroplasmatales | Erysipelotrichaceae | sf_3 | 3965 | AY133091.1 | TCE-contaminated site clone ccslm238 |
| Bacteria | Chloroflexi | Anaerolineae | Unclassified | Unclassified | sf_9 | 205 | AJ532729.1 | uranium mining waste pile clone JG34-KF-221 |
| Bacteria | Firmicutes | Clostridia | Clostridiales | Lachnospiraceae | sf_5 | 2668 | AB088998.1 | termite gut homogenate clone Rs-G40 bacterium |
| Bacteria | Firmicutes | Clostridia | Clostridiales | Lachnospiraceae | sf_5 | 2994 | AB100463.1 | termite gut clone Rs-L15 |
| Bacteria | Firmicutes | Bacilli | Lactobacillales | Streptococcaceae | sf_1 | 3422 | X68418.1 | Streptococcus thermophilus str. DSM 20617 |
| Bacteria | Firmicutes | Bacilli | Lactobacillales | Streptococcaceae | sf_1 | 3685 | AF003931.1 | Streptococcus gordonii str. ATCC 10558 |
| Bacteria | Bacteroidetes | Sphingobacteria | Sphingobacteriales | Unclassified | sf_6 | 5439 | AF507859.1 | Mono Lake at depth 35 m station 6 July 2000 clone ML635J-40 bacterium |
| Bacteria | Bacteroidetes | Flavobacteria | Flavobacteriales | Flavobacteriaceae | sf_1 | 5352 | AY871820.2 | Riemerella anatipestifer str. H-2565 |
| Bacteria | Proteobacteria | Epsilonproteobacteria | Campylobacterales | Unclassified | sf_1 | 10530 | AY672531.1 | hydrothermal vent 9 degrees North East Rise Pacific Ocean clone CH5_6_BAC_16SrRNA_9N_EPR |
| Bacteria | Chloroflexi | Dehalococcoidetes | Unclassified | Unclassified | sf_1 | 2497 | AF524015.1 | forested wetland clone FW60 |
| Bacteria | Firmicutes | Bacilli | Lactobacillales | Streptococcaceae | sf_1 | 3446 | AY173079.1 | Streptococcus bovis str. HJ50 |
| Bacteria | Bacteroidetes | Bacteroidetes | Bacteroidales | Unclassified | sf_15 | 5874 | AJ441239.1 | hydrothermal vent polychaete mucous clone P. palm A 53 |
| Bacteria | Unclassified | Unclassified | Unclassified | Unclassified | sf_160 | 6430 |  | |
| Bacteria | Bacteroidetes | Sphingobacteria | Sphingobacteriales | Crenotrichaceae | sf_11 | 6249 | M58790.2 | Haliscomenobacter hydrossis |
| Bacteria | Chloroflexi | Anaerolineae | Unclassified | Unclassified | sf_9 | 375 | AF507690.1 | forest soil clone C043 |
| Bacteria | Proteobacteria | Gammaproteobacteria | Alteromonadales | Alteromonadaceae | sf_1 | 9324 | AF316891.1 | Pseudoalteromonas ruthenica str. KMM300 |
| Bacteria | Proteobacteria | Epsilonproteobacteria | Campylobacterales | Campylobacteraceae | sf_3 | 10538 | L14624.1 | Arcobacter cryaerophilus |
| Bacteria | Proteobacteria | Epsilonproteobacteria | Campylobacterales | Campylobacteraceae | sf_3 | 10447 | Y13671.1 | Sulfurospirillum deleyianum str. Spirillum 5175 |
| Bacteria | Actinobacteria | Actinobacteria | Actinomycetales | Unclassified | sf_3 | 1405 | X80744.1 | Arthrobacter ureafaciens str. DSM 20126 |
| Bacteria | Firmicutes | Bacilli | Lactobacillales | Streptococcaceae | sf_1 | 3499 | AF104676.1 | Streptococcus constellatus str. ATCC27823 |
| Bacteria | Thermodesulfobacteria | Thermodesulfobacteria | Thermodesulfobacteriales | Thermodesulfobacteriaceae | sf_1 | 667 | AF411013.1 | Geothermobacterium ferrireducens |
| Bacteria | Bacteroidetes | KSA1 | Unclassified | Unclassified | sf_1 | 5951 | AF449785.1 | CFB group clone ML615J-4 |
| Bacteria | Unclassified | Unclassified | Unclassified | Unclassified | sf_160 | 226 |  | |
| Bacteria | Firmicutes | gut clone group | Unclassified | Unclassified | sf_1 | 4616 | AB185532.1 | rumen clone F23-C12 |
| Bacteria | Chloroflexi | Unclassified | Unclassified | Unclassified | sf_2 | 818 |  | |
| Bacteria | Acidobacteria | Acidobacteria | Acidobacteriales | Acidobacteriaceae | sf_6 | 6362 | Y07646.1 | grassland soil clone DA052 |
| Bacteria | Actinobacteria | Actinobacteria | Actinomycetales | Unclassified | sf_3 | 1687 | AJ626896.1 | Jonesia quinghaiensis str. DSM 15701 |
| Bacteria | Firmicutes | Catabacter | Unclassified | Unclassified | sf_1 | 4293 | AB089008.1 | termite gut homogenate clone Rs-Q01 bacterium |
| Bacteria | marine group A | mgA-2 | Unclassified | Unclassified | sf_1 | 6344 | AF382142.1 | bacterioplankton clone ZA3648c |
| Bacteria | Firmicutes | Clostridia | Clostridiales | Peptococc/Acidaminococc | sf_11 | 992 | AJ229198.1 | anoxic bulk soil flooded rice microcosm clone BSV43 clone |
| Bacteria | Cyanobacteria | Cyanobacteria | Chloroplasts | Chloroplasts | sf_5 | 5147 | X82156.1 | Emiliania huxleyi str. Plymouth Marine Laborator PML 92 |
| ^a^ S-F, Sub-family; ^b^ TID, Taxon identifier^; c^ , Representative species for taxon. | | | | | | | | |
