# Supplementary material for: Airway Microbiota and Pathogen Abundance in Age-Stratified Cystic Fibrosis Patients
Source: PLoS One. 2010 Jun 23;5(6):e11044. doi: 10.1371/journal.pone.0011044 (PMC2890402; doi:10.1371/journal.pone.0011044)
Supplement: Table S4 — Patient demographics. (0.09 MB DOC) [file pone.0011044.s004.doc]

| **Table S4. Patient demographics** | | | | | |
| --- | --- | --- | --- | --- | --- |
| **Study ID** | **FEV1** | **Severity** | **Age** | **Gender** | **Mutation** |
| CF16S_01 | n/a | n/a | 25 | M | dF508/dF508 |
| CF16S_03 | 46 | Moderate | 43 | M | dF508/6542X |
| CF16S_05 | 68 | Moderate | 25 | F | dF508/R1066C |
| CF16S_06 | 61 | Moderate | 37 | M | 3849+10kbC/3849+10kbC |
| CF16S_07 | 52 | Moderate | 26 | M | G524X/Unknown |
| CF16S_09 | 60 | Moderate | 26 | F | G551D/Unknown |
| CF16S_11 | 107 | Normal | 24 | M | dF508/dF508 |
| CF16S_13 | 75 | Mild | 30 | M | dF508/W1282X |
| CF16S_14 | 91 | Normal | 22 | M | dF508/dF508 |
| CF16S_17 | 55 | Moderate | 23 | F | dF508/dF508 |
| CF16S_18 | 47 | Moderate | 31 | M | dF508/Unknown |
| CF16S_19 | 82 | Mild | 33 | F | dF508/dF508 |
| CF16S_22 | 39 | Severe | 29 | F | Unknown/Unknown |
| CF16S_24 | 43 | Moderate | 29 | M | G542X/W1282X |
| CF16S_25 | 80 | Mild | 72 | M | G542X/Unknown |
| CF16S_26 | 84 | Mild | 35 | F | dF508/3659delC |
| CF16S_31 | 37 | Severe | 56 | M | dF508/dF508 |
| CF16S_32 | 92 | Normal | 24 | F | dF508/G551D |
| CF16S_33 | 78 | Mild | 29 | M | dF508/dF508 |
| CF16S_36 | 59 | Moderate | 39 | F | dF508/G542X |
| CF16S_37 | 32 | Severe | 30 | M | dF508/dF508 |
| CF16S_42 | 41 | Moderate | 20 | F | dF508/dF508 |
| CF16S_44 | 46 | Moderate | 21 | F | R553X/Unknown |
| CF16S_45 | 57 | Moderate | 58 | M | A455E/Uknown |
| CF16S_46 | 52 | Moderate | 53 | M | G542X/ 3849+10kbC |
| CF16S_47 | 43 | Moderate | 63 | M | 2789+5/dF508 |
| CF16S_48 | 74 | Moderate | 29 | M | dF508/Unknown |
| CF16S_49 | 98 | Normal | 29 | M | Unknown/Unknown |
| CF16S_50 | 69 | Moderate | 33 | F | dF508/dF508 |
| CF16S_51 | 30 | Severe | 40 | M | dF508/Unknown |
| CF16S_53 | 67 | Moderate | 26 | F | dF508/M1101R |
| CF16S_56 | 84 | Mild | 23 | M | dF508/W1282X |
| CF16S_57 | 93 | Normal | 23 | F | dF508/dF508 |
| CF16S_58 | 90 | Normal | 20 | M | dF508/dF508 |
| CFP16S_01 | n/a | n/a | 3 | M | dF508/dF508 |
| CFP16S_02 | n/a | n/a | 2 | F | dF508/R1162X |
| CFP16S_04 | n/a | n/a | 0.92 | F | dF508/dF508 |
| CFP16S_05 | 114.6 | Normal | 8 | F | dF508/Unknown |
| CFP16S_06 | n/a | n/a | 0.93 | F | Unknown/Unknown |
| CFP16S_07 | 126.3 | Normal | 11 | F | dF508/A455E |
| CFP16S_08 | n/a | n/a | 0.77 | M | dF508/dF508 |
| CFP16S_09 | 75.01 | Moderate | 6 | F | dF508/dF508 |
| CFP16S_10 | 111.11 | Normal | 8 | F | dF508/R1066C |
| CFP16S_11 | 68.95 | Moderate | 11 | F | dF508/384910kbC |
| CFP16S_12 | n/a | n/a | 2 | M | dF508/dF508 |
| CFP16S_14 | 43.7 | Moderate | 6 | F | S549N/181116kbAG |
| CFP16S_15 | 34.01 | Severe | 17 | F | dF508/dF508 |
| CFP16S_16 | 95.56 | Normal | 17 | M | df508/W1282X |
| CFP16S_18 | n/a | n/a | 5 | M | dF508/Unknown |
| CFP16S_19 | 104.76 | Normal | 10 | M | dF508/dF508 |
| CFP16S_20 | n/a | n/a | 5 | F | Unknown/Unknown |
| CFP16S_23 | 80.8 | Mild | 8 | F | dF508/dF508 |
| CFP16S_25 | n/a | n/a | 19 | F | df508/S466XTGA |
| n/a, Not available | | | | | |
