# Supplementary figures and images for: Airway Microbiota and Pathogen Abundance in Age-Stratified Cystic Fibrosis Patients
Source: PLoS One. 2010 Jun 23;5(6):e11044. doi: 10.1371/journal.pone.0011044 (PMC2890402; doi:10.1371/journal.pone.0011044)

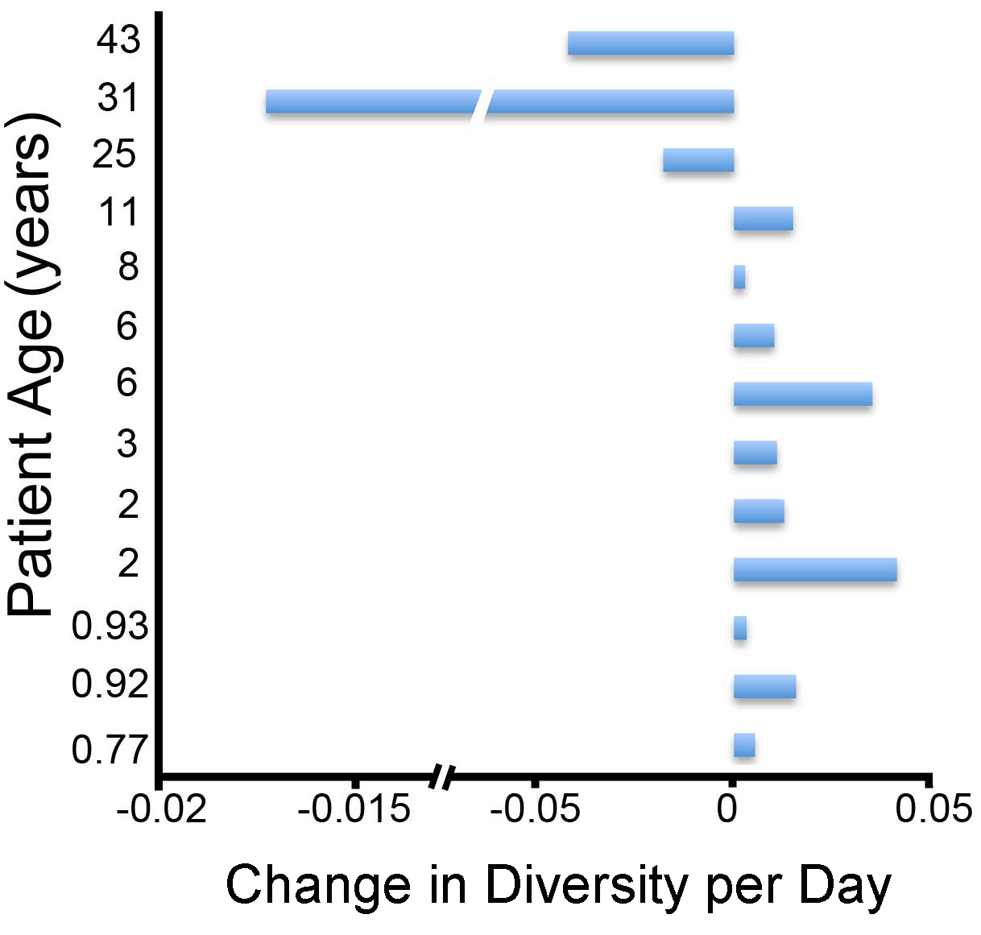

Supplement: Figure S1 — Change in bacterial community diversity over time. Change in diversity, normalized to length of time between sample collection points was calculated for CF patients ranging in age from 9 months to 43 years old and illustrates an initial net increase in diversity (per day) in younger patients in comparison with a decrease in diversity in older patients. (0.18 MB TIF) [file pone.0011044.s005.tif]
